# Electrochemical Synthesis of Organic Polysulfides from Disulfides by Sulfur Insertion from S_8_ and an Unexpected Solvent Effect on the Product Distribution

**DOI:** 10.1002/chem.202101023

**Published:** 2021-06-01

**Authors:** Jan Fährmann, Gerhard Hilt

**Affiliations:** ^1^ Institut für Chemie Universität Oldenburg Carl-von-Ossietzky-Straße 9–11 26111 Oldenburg Germany

**Keywords:** electrosynthesis, polysulfides, sulfur, solvent effects, trisulfides

## Abstract

An electrochemical synthesis of organic polysulfides through sulfur insertion from elemental sulfur to disulfides or thiols is introduced. The highly economic, low‐sensitive and low‐priced reaction gives a mixture of polysulfides, whose distribution can be influenced by the addition of different amounts of carbon disulfide as co‐solvent. To describe the variable distribution function of the polysulfides, a novel parameter, the “absorbance average sulfur amount in polysulfides” (SAP) was introduced and defined on the basis of the “number average molar mass” used in polymer chemistry. Various organic polysulfides were synthesized with variable volume fractions of carbon disulfide, and the yield of each polysulfide was determined by quantitative ^13^C NMR. Moreover, by using two symmetrical disulfides or a disulfide and a thiol as starting materials, a mixture of symmetrical and asymmetrical polysulfides could be obtained.

## Graphical abstract

**Electrifying Sulfur**! The electrolysis of organic disulfides in dichloromethane led to the formation of organic polysulfides with a high incorporation of additional sulfur atoms. HPLC/HRMS analysis of the reaction mixture could identify products up to undecasulfides. When CS_2_ was used as co‐solvent the distribution of the organic polysulfide mixture was altered in favor of the lower polysulfides, such as tri‐ and tetrasulfides.

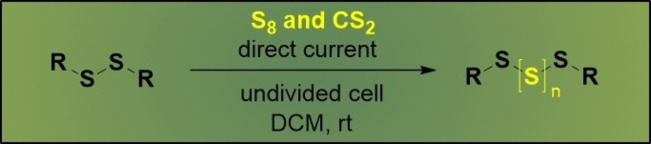

## Introduction

Organosulfides play an important role in various parts of everyday life. Thiols flavor food like onion, cheese and garlic.^[1]^ Amino acids like cysteine contain thiols to crosslink peptides forming disulfides, such as keratin in human hair.^[2]^ Common stinkhorn's (*Phallus impudicus*) intense smell is produced by dimethyl disulfide and dimethyl trisulfide (DMTS).^[3]^ Even higher polysulfides can be found in nature, lenthionine from shiitake mushrooms serves as an example.^[4]^ A high level of interest is currently given to the inorganic lithium‐sulfur (Li−S) batteries. Due to their excellent specific capacity and energy density, Li−S batteries may play an important role in global energy transition, specifically in electric cars and portable device‐research.^[5]^ Recent studies from qian and wen showed that the addition of organic trisulfides like DMTS to Li−S batteries “enhances the sulfur utilization rate and facilitates capacity performance”.^[6]^ In the battery, the trisulfide reacts via sulfur insertion to organic polysulfides, which then form the soluble lithium organo‐polysulfanes and insoluble lithium sulfide – the driver for an enhanced sulfur utilization rate.[^6^, ^7^]

**Scheme 1:**
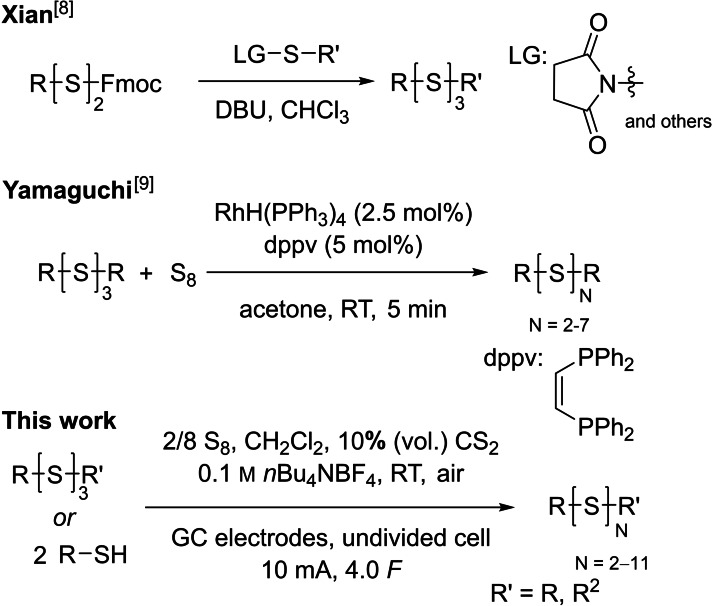
Previous work in organic polysulfide synthesis.

Several publications of the past few years deal with the synthesis of organic polysulfides. In 2018, xian et al. reported a new route for the synthesis of asymmetrical trisulfides.^[8]^ The reaction of nucleophilic 9‐fluorenylmethyl (Fmoc) disulfides with electrophilic *S*‐succinimide derivatives generates the desired mixed trisulfides (Scheme 1). Several asymmetrical trisulfides could be synthesized in high yields using this method. However, the reaction is laborious and not highly atom‐economic. For example, the desired reactants need to be synthesized from thiols before the coupling can be performed. Also, this synthetic route is limited to the formation of trisulfides. The use of elemental sulfur in organic synthesis is rare – especially in polysulfide synthesis. One single approach was presented by yamaguchi et al. in 2005. They discovered that a rhodium‐catalyzed reaction exchanges sulfur atoms between elemental sulfur and organic disulfides for the synthesis of a range of organic polysulfides.^[9]^ Thereby, organo‐polysulfides (up to heptasulfides were detected) could be synthesized in a short reaction time (5 min). However, the expensive transition metal rhodium, albeit used in catalytic amounts, is needed. The electro‐organic synthesis is currently in the focus of many scientists due to its mild, efficient and mostly low‐priced reaction setup.^[10]^ An easy way for the electrochemical synthesis of asymmetrical disulfides was reported last year from our group. Using alternating current electrolysis, we were able to obtain a statistical distribution of disulfides, starting from two or more symmetrical disulfides.^[11]^ This fast and highly atom‐economic process produced only disulfides and no trisulfides or higher organic polysulfides were found.

In this article, we present an electrochemical approach for the synthesis of organic trisulfides and higher sulfides from their corresponding disulfides or thiols. We developed an easy, robust and low‐priced method without the need for any catalysts or additives to generate organic polysulfides.

## Results and Discussion

### Optimization

**Figure 1:**
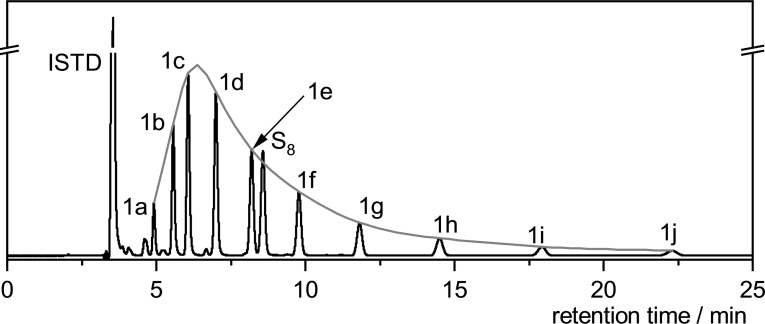
Representative RP‐HPLC‐UV chromatogram of the crude reaction mixture **1 a**–**1 j** at 248 nm with methanol as eluent. Benzophenone was used as internal standard (ISTD).

When we initially investigated the electrochemical organo‐polysulfide synthesis, we adopted the reaction conditions reported for the disulfide metathesis reaction,^[11]^ using acetonitrile as solvent, tetrabutylammonium tetrafluoroborate (*n*Bu_4_NBF_4_) as supporting electrolyte and platinum electrodes in an undivided cell. Two atom‐equivalents of sulfur‐atoms (=2.0 [S])^[12]^ were added to di‐*n*‐butyl disulfide (DBDS, **1 a**) and the pH neutral solution was electrolyzed at 10 mA constant current under non‐inert conditions (Table 1, entry 1). Monitoring the reaction progress by reversed‐phase HPLC with UV‐detector (RP‐HPLC‐UV) indicated 68 % conversion of the disulfide **1 a**, 85 % conversion of sulfur and the formation of polysulfides up to the undecasulfide **1 j** in an acidic product solution. A representative chromatogram is shown in Figure 1. The determination of the yield from HPLC‐UV analysis is challenging due to the unknown absorption coefficients of the polysulfides. Separation of the polysulfide mixture is not possible via simple silica‐gel chromatography due to their very similar polarity and a preparative HPLC was not available for us at that time. Also, the detection of polysulfides from a sulfur‐chain length of four or more by GC‐FID could not be achieved. Luckily, the distribution of polysulfides was not affected by different reaction conditions – a circumstance applying to the synthesis route from yamaguchi as well.^[9]^ That means, the polysulfide ratio was the same for all optimization experiments, while their absolute yield changed. Thereby the conversion of the starting material and the yield of a single polysulfide could be used for the optimization of the reaction conditions. Since the starting material **1 a** and trisulfide **1 b** can be detected by GC‐FID, we chose this fast and precise method for the optimization. Therefore, it is important to keep in mind that the yield of the trisulfide does not reflect the overall yield of the polysulfides, it rather serves as an indicator for a higher/lower yield in the polysulfide formation **1 b**–**1 j**.

**Table 1:** Optimization experiments.

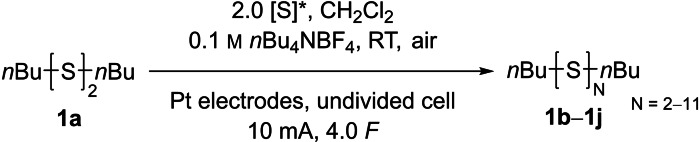			
	Changes from initial conditions	Conversion of **1 a** ^[a]^	Yield of **1 b** ^[a]^
1	acetonitrile as solvent	68 %	27 %
2	none	71 %	31 %
3	*n*Bu_4_NPF_6_ as electrolyte	71 %	24 %
4	*n*Bu_4_NBr as electrolyte	69 %	28 %
5	*n*Bu_4_NOTos as electrolyte	0 %	0 %
6	Et_4_NClO_4_ as electrolyte	96 %	7 %
7	0.05 m *n*Bu_4_NBF_4_	72 %	28 %
8	0.30 m *n*Bu_4_NBF_4_	67 %	27 %
9	0.50 m *n*Bu_4_NBF_4_	62 %	23 %
10	stainless steel electrodes	0 %	0 %
11	Ni electrodes	34 %	0 %
12	Cu electrodes	40 %	0 %
13	graphite electrodes	71 %	30 %
**14**	**GC electrodes**	**74 %**	**35 %**
15	5 mA	71 %	31 %
16	20 mA	70 %	30 %
17	40 mA	78 %	18 %
18	0 °C	73 %	35 %
19	35 °C	74 %	30 %
20	quasi‐divided cell	69 %	23 %
21	divided cell (anolyte)^[b]^	75 %	10 %
22	divided cell (catholyte)^[b]^	13 %	0 %
23	inert atmosphere	74 %	35 %
24	as entry 23+anhydrous solvent	70 %	29 %
25	as entry 24+2.0 equiv. H_2_O	74 %	35 %
26	1.0 mmol scale^[c]^	76 %	36 %
27	0.0 *F* (160 min stirring)	0 %	0 %
28	6.5 *F*	77 %	35 %

Unless otherwise stated, 0.25 mmol disulfide **1 a** (1.0 equiv.) were used in a total volume of 10 mL. Dimensions of the electrodes 35x10x0.5 mm, immersion depth of the electrodes 1.5 cm, electrode spacing 1.5 cm. Changes from highlighted entries were taken on following experiments. *2 [S]=2/8 S_8_. [a] Determined by GC‐FID analysis of the crude reaction mixture with *n*‐dodecane as internal standard. [b] A 0.3 m electrolyte concentration was used. [c] Total volume was 20 mL.

Because the solubility of sulfur in acetonitrile is rather low, we varied the solvent at the beginning of the reaction optimization. We tested various solvents (tetrahydrofuran, dimethoxyethane, acetone, dimethyl sulfoxide, dimethylformamide, ethanol, propan‐2‐ol and dichloromethane). Of all tested solvents, only dichloromethane was also suitable for the polysulfide synthesis. In all other solvents, the sulfur remained insoluble during the electrolysis or side reactions with the solvent took place. We decided to use CH_2_Cl_2_ as a significantly better sulfur‐solubilizing solvent than acetonitrile for the further investigations (entry 2). Thereby the conversion of the starting material increased slightly to 71 %. In the following, we varied the electrolyte and its concentration in solution (entries 2–9). Formation of the polysulfides **1 b**–**1 j** remained high when *n*Bu_4_NPF_6_ was applied as supporting electrolyte (71 % conversion, 24 % yield of **1 b**, entry 3). Using *n*Bu_4_NBr as a low‐priced supporting electrolyte is a good alternative to the BF_4_ salt, as the formation of the trisulfide remained high (28 % of **1 b**, entry 4). While the application of *n*Bu_4_NClO_4_ resulted in decomposition of the starting material (entry 5), as no by‐products were detected by GC‐FID or HPLC‐UV, the use of *n*Et_4_NOTs led to a complete collapse of disulfide conversion (entry 6). Due to the good results and high conductivity, we decided to use *n*Bu_4_NBF_4_ as the electrolyte of choice, but *n*Bu_4_NBr is also recommended as less expensive alternative. The concentration of the supporting electrolyte can be varied between 0.05 and 0.3 m without significant reduction of product formation (entries 7‐9). An electrolyte concentration of 0.5 m is accompanied with a slight decrease of product formation (62 % conversion of **1 a** to 23 % **1 b**, entry 9). For the best results and a high conductivity, we decided to hold on to the initial electrolyte concentration of 0.1 m.

When we varied the electrode materials, stainless steel did not give any conversion of the starting material (entry 10) and nickel as well as copper electrodes gave a low conversion, however, no polysulfides were observed by GC‐FID and HPLC‐UV (entries 11 and 12). Thus, we assumed decomposition of the disulfide when using these electrodes. On the other hand, graphite electrodes (see the Supporting Information) proved to be a good alternative to platinum electrodes, whose results in conversion and yield were almost identical (entry 13). When glassy‐carbon (GC) electrodes were used, 35 % trisulfide **1 b** were formed at a conversion of 74 % **1 a**, so we decided to use GC electrodes for further optimization.

The applied current was varied between 5 and 40 mA (entries 15–17). Further increase above 40 mA caused too much evolution of heat, leading to evaporation of the CH_2_Cl_2_ and collapse of the reaction. A low current between 5 and 20 mA did not affect the conversion at all (entries 15 and 16), however a high current of 40 mA led to decomposition of the starting material (78 % conversion of **1 a**, 18 % yield of **1 b**, entry 17). For a reasonably fast product formation and a high yield, we decided to keep the applied current at 10 mA, but for an even faster reaction 20 mA can be applied without the risk of significant loss of polysulfide products.

Decreasing the reaction temperature to 0 °C does not have an impact on the polysulfide synthesis (entry 18), but increasing it to 35 °C reduced the formation of **1 b** to 30 % (entry 19). In order to keep the setup as simple as possible, we decided to perform further electrolysis at ambient temperature (ca. 21 °C). Entry 20 shows that varying the cell design does not improve the formation of organic polysulfides. When the reaction was performed in a quasi‐divided cell^[13]^ the yield of trisulfide **1 b** dropped to 23 %. To maintain a high conductivity in a divided cell, an electrolyte concentration of 0.3 m was necessary. In the anode compartment only 10 % trisulfide were detected (entry 21), while in the cathode compartment no organic polysulfides were formed (entry 22). Accordingly, the simplest, and, with respect to the efficiency of the reaction, the best design is an undivided cell. The optimization experiments were carried out under non‐inert conditions with undried solvents and supporting electrolytes. In entry 23 we demonstrate that an inert atmosphere did not improve the polysulfide synthesis, additionally working under anhydrous conditions even lowered the yield of the trisulfide **1 b** to 29 % (entry 24). When two equivalents of water were added to the anhydrous solvent (entry 25) identical results were obtained to those for electrolysis under non‐inert conditions, thus implying that small amounts of water are useful for this reaction.

The reaction could be performed on a larger scale; for example, the scale could be quadrupled while doubling the amount of solvent (entry 26) without loss of efficiency for the formation of organic polysulfides. The higher concentration and amount of substrates, associated with a longer reaction time, were well tolerated. Finally, in entry 27 we set the standard conditions without applying any current. After 160 min of stirring (= electrolysis time at standard conditions), no conversion of the starting material and no organic polysulfides were observed, which in conclusion proves that electrochemical current is needed to perform this reaction.

**Figure 2:**
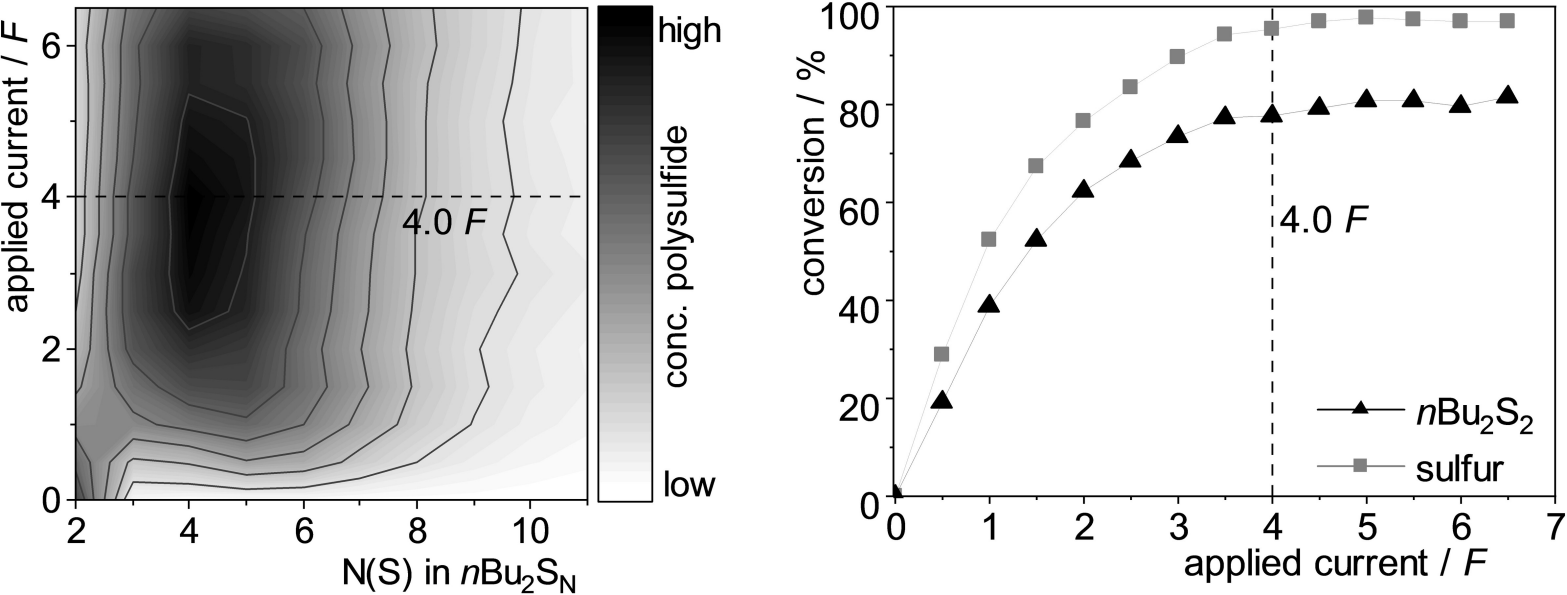
Graphical presentations of (left) the relative amount of organic polysulfides formed as determined by HPLC‐UV and (right) conversion of the starting materials **1 a** and elemental sulfur depending on the applied current.

Figure 2 (left) portrays the relative distribution of the organic polysulfides **1 a**–**1 j** depending on the amount of applied current. As can be seen, the maximum amount of polysulfides is reached after 4.0 *F*. Figure 2 (right) confirms a plateau in conversion of disulfide **1 a** and sulfur at 4.0 *F* and further electrolysis of up to 6.5 *F* did not change the polysulfide distribution mixture. Only a very slight decrease in the polysulfide yield was observed due to decomposition while the polysulfide distribution remained identical (Figure 2 left, Table 1, entry 28). A possible explanation for the need of 4.0 *F* of current is discussed in the mechanistic studies part.

### Solvent effect on the polysulfide distribution

During preliminary tests, we observed a correlation between the amount of sulfur added to the reaction and the polysulfide distribution. Additionally, the volume fraction of added carbon disulfide (***CAUTION**! CS_2_ should be handled with care*) also affected the distribution. A manual adjustment of distributions from polysulfide synthesis with elemental sulfur has not been reported so far. Consequently, we decided to investigate the solvent effect of CS_2_ on the polysulfide distribution in more detail. For their analysis, we referenced the HPLC‐UV integral of each experiment to an internal standard (benzophenone), a representative HPLC‐UV chromatogram is shown in Figure 1. With this, we were able to determine the conversion of sulfur and disulfide **1 a** as well as the referenced HPLC‐UV integrals of organic polysulfides **1 b**–**1 j** (Figure 1). To set up a mathematical comparison of the integral ratios, we introduced the “absorbance average sulfur amount in polysulfides at 248 nm” (SAP_248_) analogous to the “number average molar mass” used in polymer chemistry,[^14^, ^15^] given as:

(1) $${\mathrm{SAP}}_{248}=\frac{\sum_{i=1}^{\infty }n_{i,248}\cdot N_{i}\left(S\right)}{\sum_{i=1}^{\infty }n_{i,248}}$$

$n_{i,\;248}:$ referenced HPLC‐integral of polysulfide i at 248 nm $N_{i}\left(S\right):$ number of sulfur equivalents in polysulfide i

  

This value allows the comparison of experiments with different polysulfide distributions. Similar to the “number average molar mass”, the SAP reflects the position of the maximum of polysulfides’ distribution function. But, the SAP is depending on the absorption coefficient‐relations of the polysulfides. Therefore, the SAP is suitable for comparison within one sulfide species but does not give information about the actual favored sulfide or absolute yield. Additionally, we calculated the dispersity *Đ* as applied in polymer chemistry (see the Supporting Information for the calculation).[^15^, ^16^] The dispersity is given as a unified number and is always ≥ 1. A dispersity of 1.0 describes a uniform distribution, and therefore a single sulfide, for example, 100 % trisulfide formation. The higher the dispersity, the more unspecific a single sulfide is formed.

**Figure 3:**
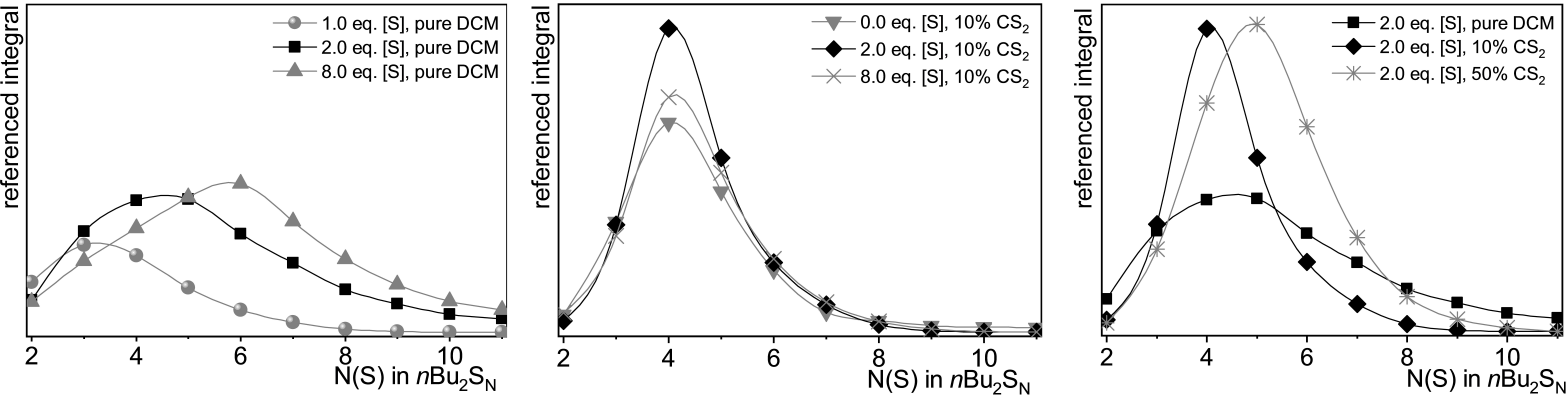
Graphical presentation of the referenced HPLC‐UV integrals showing dependence on left: the amount of sulfur added in pure CH_2_Cl_2_, centre: the amount of sulfur amount added in CH_2_Cl_2_ with 10 % (vol.) CS_2_, and right: the amount of CS_2_. See Table 2 for further information and the Supporting Information for all graphs (colourized).

Two atom‐equivalents of sulfur (2.0 [S]), as used in the optimization experiments, gave the highest conversion rate of the disulfide **1 a** (74 %) and an almost quantitative conversion of sulfur (95 %). The SAP was 5.34 at a dispersity of 1.13 (Table 2, entry 4). Using up to four atom‐equivalents of sulfur did not affect the conversion of the starting materials (entries 5–6). The SAP increased slightly up to 5.46, while the absolute consumption of sulfur stayed the same and excess sulfur simply remained in solution. The yield and distribution of polysulfides was almost identical between two and four atom‐equivalents of sulfur, further increase of the sulfur atom‐equivalents to eight equivalents, however, shifted the SAP to 5.95 (Figure 3 left, entry 7). Another indicator for this is shown by the absolute conversion of sulfur, which increased by 17 % towards two atom‐equivalents of sulfur. Reducing the amount of sulfur to one atom‐equivalent led to a sulfur‐deficiency, resulting in a 20 % lower conversion of the disulfide, accompanying with a huge shift of the SAP towards lower polysulfides (SAP 3.83, entry 3). Further sulfur‐deficiency (0.5 atom equivalents) comes along with an even lower SAP of 3.29 (entry 2). As expected, no polysulfides were formed at all without the addition of any sulfur (entry 1). In summary, the average number of sulfur atoms in polysulfides can be influenced by the amount of added sulfur atom‐equivalents. The dispersion for all these experiments is constant – but it is relatively high at 1.13 to 1.15. This is visualized by a broad maximum in Figure 3, left.

**Table 2:** Dependence of the polysulfide distribution (SAP and dispersity *Đ*) on the sulfur amount added to the reaction solution and volume fraction of CS_2_.

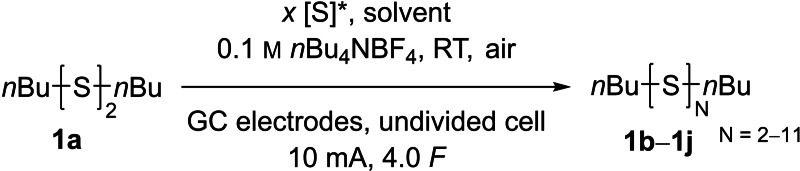					
	Conditions	Conversion of		SAP_248_	*Đ*
		**1 a**	[S]		
1	0.0 [S], pure CH_2_Cl_2_	46 %	–	–	–
2	0.5 [S], pure CH_2_Cl_2_	44 %	98 %	3.29	1.21
3	1.0 [S], pure CH_2_Cl_2_	54 %	98 %	3.83	1.13
4	2.0 [S], pure CH_2_Cl_2_	74 %	95 %	5.34	1.13
5	3.0 [S], pure CH_2_Cl_2_	74 %	63 %	5.41	1.14
6	4.0 [S], pure CH_2_Cl_2_	73 %	47 %	5.46	1.15
7	8.0 [S], pure CH_2_Cl_2_	72 %	28 %	5.95	1.13
8	0.0 [S], 10 % (vol.) CS_2_	84 %	−	4.55	1.11
9	1.0 [S], 10 % (vol.) CS_2_	84 %	98 %	4.47	1.06
10	2.0 [S], 10 % (vol.) CS_2_	84 %	91 %	4.43	1.06
11	4.0 [S], 10 % (vol.) CS_2_	86 %	45 %	4.46	1.06
12	8.0 [S], 10 % (vol.) CS_2_	80 %	18 %	4.52	1.08
13	2.0 [S], 2 % (vol.) CS_2_	85 %	73 %	4.41	1.07
14	2.0 [S], 25 % (vol.) CS_2_	88 %	90 %	4.86	1.07
15	2.0 [S], 50 % (vol.) CS_2_	93 %	90 %	5.15	1.07
16	entry 10+no current	0 %	0 %	–	–

Unless otherwise stated, 0.25 mmol disulfide **1 a** (1.0 equiv.) were used in a total volume of 10 mL. Dimensions of the electrodes 35×10×0.5 mm, immersion depth of the electrodes 1.5 cm, electrode spacing 1.5 cm. See Figure 3 for a graphical presentation of the referenced integral. *x [S]=*x*/8 S_8_. [a] Determined by HPLC‐UV analysis at 248 nm of the crude reaction mixture with benzophenone as internal standard.

When a solvent‐mixture of CH_2_Cl_2_/CS_2_ with 10 % (vol.) is used with two atom‐equivalents of sulfur, the conversion of the starting material increased to 86 % (entry 10). Moreover, the SAP decreases to 4.43, and the dispersity is considerably reduced to 1.06, thus giving a significantly narrower distribution of polysulfides. This finding is impressively visualized in Figure 3. For this 10 : 1 mixture, varying the atom‐equivalents of sulfur between one to eight did not affect the dispersity, which stayed constant between 1.06 to 1.08 (entries 9–12). Different to the experiments in pure CH_2_Cl_2_, the SAP is also quiet constant between 4.43–4.52. Only the absolute yield of the polysulfides changed clearly (see Figure 3). The maximum yield of polysulfides is between two to four atom‐equivalents sulfur (entries 10/11) as it was the same as for the experiments in pure CH_2_Cl_2_. Interestingly, a relatively high conversion of disulfide **1 a** to polysulfides was observed even without addition of any sulfur (entry 8). It seems that CS_2_ does not only act as a pure solvent, but unfortunately we could not clarify its exact role in this reaction as cyclic voltammetry and other spectroscopic methods did not give meaningful results in this respect. To affect the SAP when using a CH_2_Cl_2_/CS_2_ mixture, the amount of added CS_2_ was varied. Again, the dispersity remains the same over a wide range. Between a proportion from 2 % (vol.) to 50 % (vol.) the dispersity was constant at 1.07, displaying an extreme narrow distribution (entries 13–15). The SAP however depended highly on the solvent ratio. It appears that the higher the amount of CS_2_ the higher the SAP (Figure 3, right). While the SAP was 4.41 at 2 % (vol.) CS_2_, it rose gradually up to 5.15 at 50 % (vol.) CS_2_ solvent content. The latter conditions gave the highest conversion of the disulfide of all experiments (93 %, entry 15) due to its narrow dispersity at high polysulfides.

### Substrate scope

Due to the strong dependence of the polysulfide distribution on the solvent mixture, we decided to continue with two different reaction setups. In setup **A**, two atom‐equivalents of sulfur were used in pure CH_2_Cl_2_ as solvent. As shown in Table 2, a wide distribution of polysulfides was expected for these reactions. In setup **B**, 10 % (vol.) CS_2_ were added, promising a noticeably narrower dispersity and probably a higher conversion of the starting material. For the investigation of the substrate scope, we determined the absolute yield of the organic polysulfides by integration of the signals of all separated *α*‐carbons (R−[S]_N_−CH_2_‐signals) in quantitative ^13^C NMR using an internal standard. Unseparated polysulfides were summed up under “further” polysulfides. For disulfide **1 a**, we already performed extensive investigations in Table 2. However, only relative results were given, depending on the absorption coefficients of the polysulfides. In ^13^C NMR, the quantitative amount of the polysulfide mixture **1 b**–**1 j** was detected (Table 3, entry 1), which confirms that no decomposition reactions occurred during electrolysis. Also, the conversion of the disulfide **1 a** (determined by ^13^C NMR) matched the conversion calculated from integration of the HPLC and GC signals (74 % HPLC and GC, 76 % NMR in setup **A**; 84 % and 81 % in setup **B**). The absolute yield of the trisulfide **1 b** was also consistent to the GC results within an acceptable margin of error (35 % GC, 30 % NMR). Overall, a good comparability between these analytical methods was determined.

**Table 3:** Substrate scope.

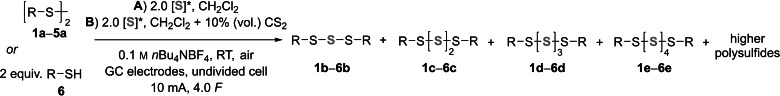												
	Substrate		Setup	Conversion of			Yield of^[b]^					Sum
				[S]^[a]^	**1 a**–**5 a**/**6** ^[b]^		**1 b**–**6 b**	**1 c**–**6 c**	**1 d**–**6 d**	**1 e**–**6 e**	*further* ^[c]^	
1	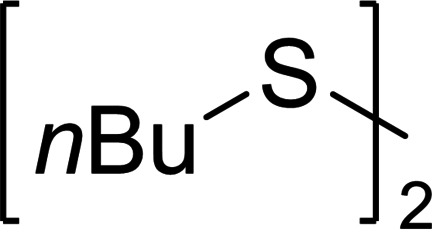	**1 a**	**A**	95 %	77 %		30 %	22 %	8 %	4 %	12 %	77 %
			**B**	94 %	81 %		41 %	24 %	3 %	2 %	8 %	78 %
												
2	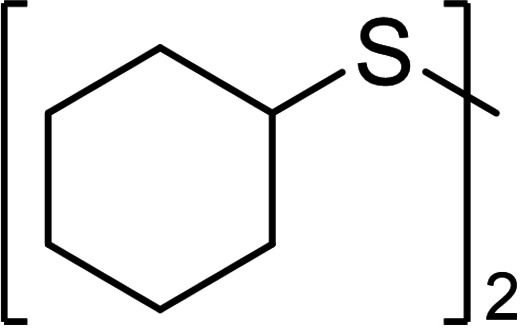	**2 a**	**A**	63 %	72 %		9 %	28 %	21 %	9 %	–	67 %
			**B**	70 %	83 %		17 %	35 %	22 %	5 %	–	79 %
												
3	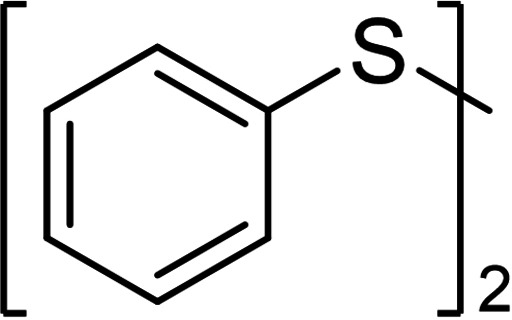	**3 a**	**A**	99 %	29 %		10 %	3 %	–	–	–	13 %
			**B**	52 %	36 %		24 %	8 %	2 %	–	–	34 %
												
4	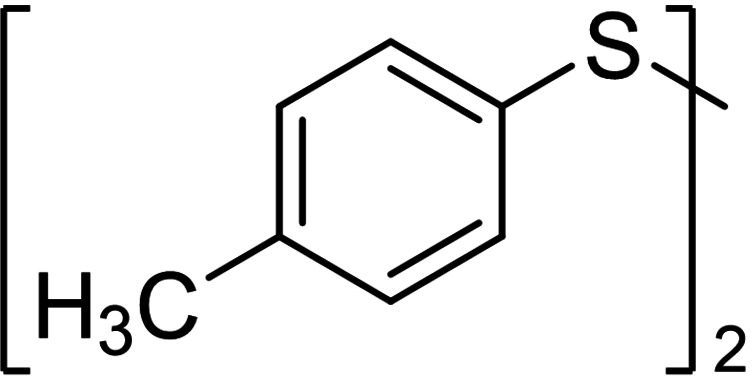	**4 a**	**A**	50 %	49 %		28 %	12 %	4 %	2 %	–	46 %
			**B**	49 %	52 %		28 %	13 %	5 %	3 %	–	49 %
												
5	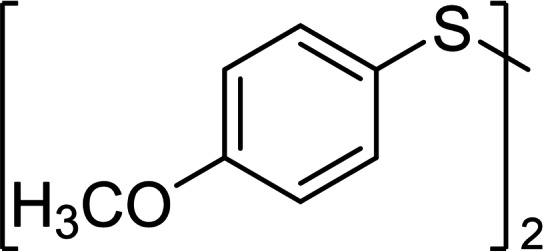	**5 a**	**A**	85 %	62 %		32 %	10 %	4 %	1 %	–	47 %
			**B**	27 %	62 %		34 %	12 %	6 %	4 %	–	56 %
												
6	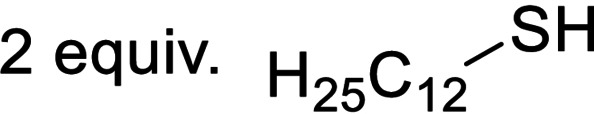	**6**	**A**	89 %	80 %^[e]^	34 % **6 a** ^[f]^	22 %	17 %	n.r.^[c]^	n.r.^[c]^	6 %	79 %
			**B**	94 %	95 %^[e]^	27 % **6 a** ^[f]^	45 %	19 %	n.r.^[c]^	n.r.^[c]^	4 %	95 %

Unless otherwise stated, 0.25 mmol disulfide **1 a**–**5 a** (1.0 equiv.) were used in a total volume of 10 mL. Dimensions of the electrodes 35×10×0.5 mm, immersion depth of the electrodes 1.5 cm, electrode spacing 1.5 cm. *2 [S]=2/8 S_8_. [a] Determined by HPLC‐UV analysis at 248 nm of the crude reaction mixture with benzophenone as internal standard. [b] Determined via quantitative ^13^C NMR by integration of the *α*‐carbons (R−[S]_N_−CH_2_‐signals) with benzophenone as internal standard. [c] The integral of unseparated signals in ^13^C NMR were summed up under “further”. Entries are marked as n.r. (= not resolved). [d] Two equivalents of the thiol were used in relation to the amount of disulfide under standard conditions. [e] Conversion of the thiol was determined, yield of the disulfide is given on the right. [f] **6 a**=di‐*n*‐dodecyldisulfide.

When **1 a** was used as starting material, the trisulfide **1 b** was the most populated species, followed by the corresponding tetrasulfide **1 c** (22 %, Table 3, entry 1). The SAP in Table 2 illustrates a population‐shift towards lower sulfides when 10 % vol. CS_2_ were added as co‐solvent. In fact, we observed a higher yield of trisulfide **1 b** (41 %) and lowered yields of the high sulfides **1 d**–**1 j** by the same ratio. For dicyclohexyl disulfide (**2 a**), a similar behavior compared to the *n*‐butyl derivate **1 a** was observed. A high conversion of **2 a** led to a wide polysulfide distribution in setup **A** (entry 2). Different to the di‐*n*‐butyl disulfide (**1 a**), the tetrasulfide **2 c** is the most populated species with 28 % followed by the pentasulfide **2 d** (21 %) and no higher polysulfides (*N*>4) were observed. Again, the conversion of the starting material increased in setup **B** (83 %). An increased yield of the trisulfide **2 b** was observed to an absolute yield of 17 %. For the tetrasulfide **2 c**, the same increase in yield was observed. Both substrates confirm a high response of dialkyl disulfides to the electrochemical polysulfide synthesis and a positive effect of CS_2_ as co‐solvent in the reaction.

Aromatic disulfides **3 a**–**5 a** showed in general a lower conversion compared to the aliphatic disulfides **1 a** and **2 a**. In pure CH_2_Cl_2_, diphenyl disulfide (**3 a**) formed 10 % trisulfide **3 b**, 3 % tetrasulfide **3 c** and no higher sulfides (entry 3, setup **A**). Also, diphenyl disulfide (**3 a**) seemed to undergo decomposition reactions due to the loss of 16 % of the polysulfide mixture, most likely caused by deposits which were observed on the cathode over the course of the electrolysis. Inexplicably, the conversion of elemental sulfur in this experiment was quantitative. When CS_2_ was added as co‐solvent in setup **B**, the decomposition of **3 a** was prevented completely. The yield of every polysulfide doubled (**3 b** 24 %, **3 c** 8 %) and the conversion of sulfur adjusted to 52 %. The electron‐rich aromatic disulfide **4 a** converted in moderate 50 % resulting in 28 % yield of trisulfide **4 b**, 13 % of tetrasulfide **4 c** and small amounts of further polysulfides. The 4‐methoxy‐substituted derivate **5 b** showed a similar distribution as the *p*‐tolyl sulfides **4 a**–**4 e** but a slightly higher conversion of the disulfide (62 %, entry 4). The distribution of both electron‐rich diaryl disulfides **3 a** and **4 a** did not change when CS_2_ was added as co‐solvent. The conversion of the disulfide **3 a**/**4 a** as well as the yields of the polysulfides **3 b**–**3 e**/**4 b**–**4 e** remained nearly stable.

In addition to disulfides, thiols can be used as starting materials for the synthesis of polysulfides as well (entry 6). For the nonvolatile thiol *n*‐dodecanethiol (**6**) also applied an improved formation of the polysulfides when CS_2_ was added as co‐solvent. When the reaction conditions of setup **B** were used, nearly full conversion of the starting material was observed after 4.0 *F*, giving a high yield of low polysulfides (45 % **6 b**, 19 % **6 c**) alongside the corresponding disulfide **6 a**.

### Synthesis of asymmetrical polysulfides

**Scheme 2:**
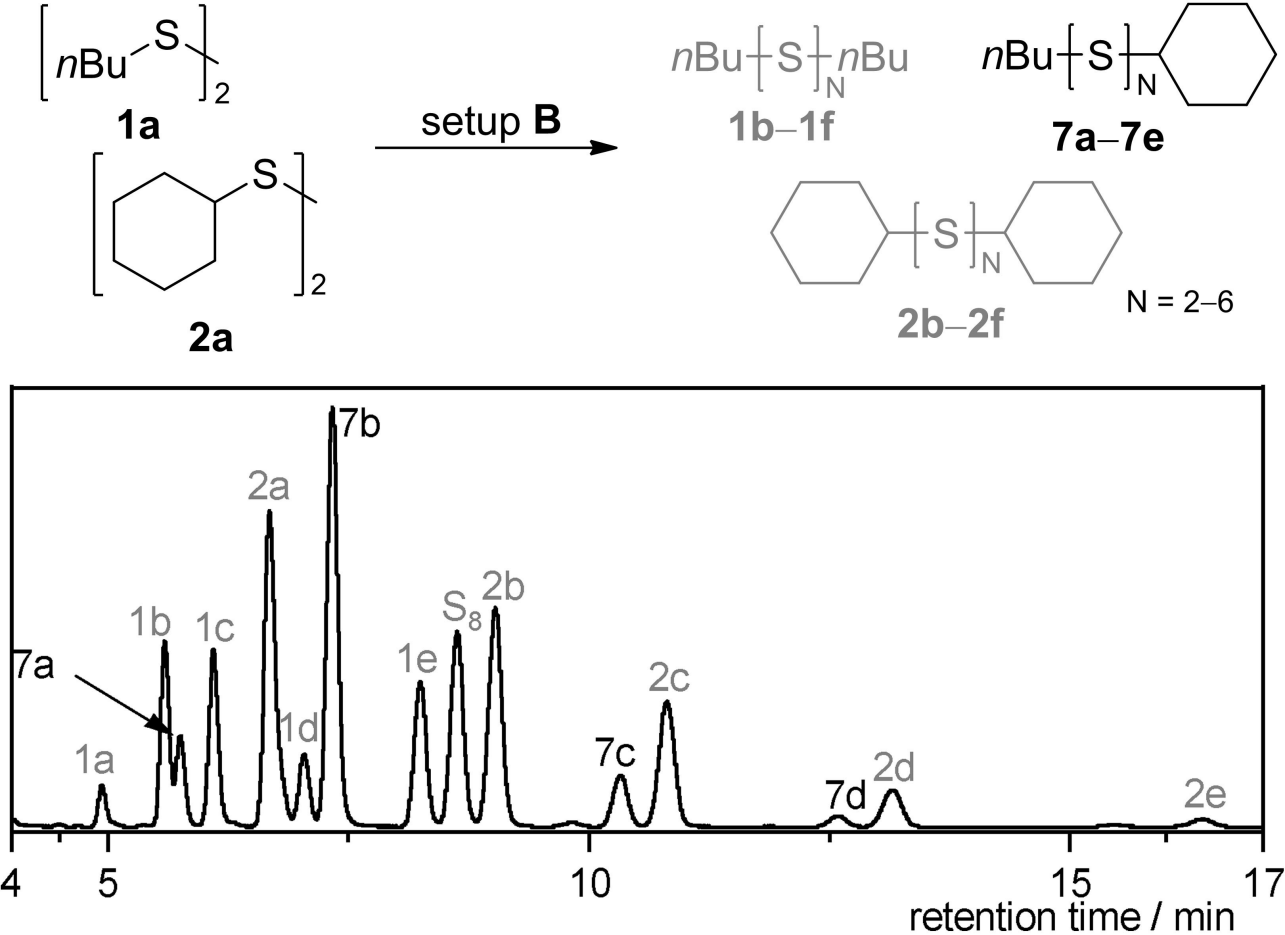
RP‐HPLC‐UV chromatogram of the crude reaction mixture **7 a**–**7 e** with **1 a**–**1 f** and **2 a**–**2 f** at 248 nm with methanol as eluent.

As the formation of polysulfides is possible from thiols, an electrochemical cleavage of the sulfur‐sulfur bond is plausible. The electrocatalytic synthesis of mixed disulfides, reported from our group last year, illustrated such a sulfur‐sulfur bond metathesis.^[11]^ To prove the possibility of mixed polysulfide‐formation for this reaction, we electrolyzed 0.5 atom‐equivalents of **1 a** and **2 a** each (in relation to the symmetrical polysulfide synthesis) at reaction setup **B**. The corresponding HPLC chromatogram is shown in Scheme 2. An assignment of the signals in ^13^C NMR could not be achieved due to the large number of signals and the determination of the yields was therefore not possible. However, secure identification of the mixed polysulfides was possible by HRMS.

**Scheme 3:**
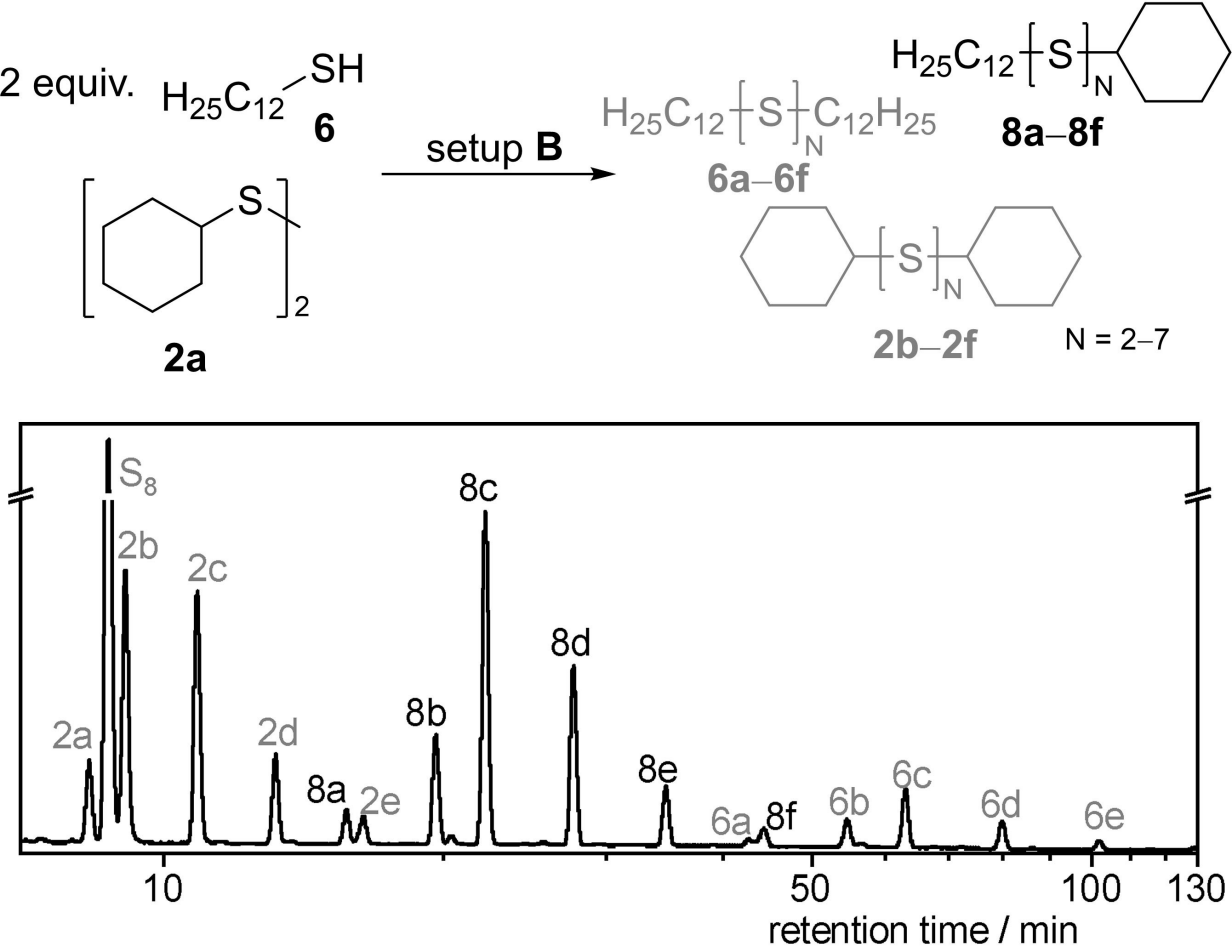
RP‐HPLC‐UV chromatogram of the crude reaction mixture **8 a**–**8 e** with **2 a**–**2 f** and **6 a**–**6 f** at 248 nm with methanol as eluent.

In the next step, we added 1.0 equivalent of thiol **6** to 0.5 equivalents of disulfide **2 a**. Again, we assigned each signal of the HPLC‐chromatogram in Scheme 3. Large amounts of the mixed polysulfides **8 a**–**8 f** were supposed and again confirmed by HRMS. These experiments showed that the formation of mixed polysulfides can be accomplished by mixing either two disulfides or a disulfide and a thiol and applying 4.0 *F* of electricity to form a large number of symmetrical and asymmetrical organic polysulfides. On the one hand, these results confirm a S−S bond cleavage according to our previous study,^[11]^ for this reaction. On the other hand, this might be useful to enlarge the application of polysulfides in dynamic libraries since many products can be synthesized from few starting materials in a single reaction.

### Mechanistic studies

**Scheme 4:**
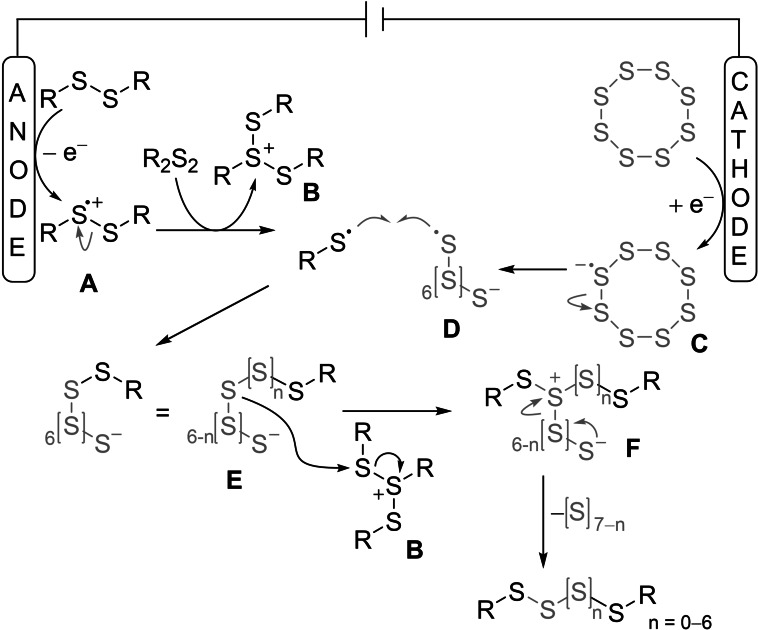
Proposed reaction mechanism.

Mechanistic studies were performed using cyclic voltammetry (see the Supporting Information), but only a few meaningful results could be obtained. However, several hints to a possible mechanism were discovered over time. Experimental studies from Table 1 confirm that the reaction can take place in the anode compartment of a divided cell, but not in the cathode compartment (entry 21/22). Hence, oxidation of the disulfide is plausible. Subsequent cleavage of the S−S bond of **A** resulting in the formation of a thiol‐radical (RS^.^) and a thiol‐cation, which is stabilized by another disulfide molecule to form intermediate **B** is plausible (Scheme 4).^[17]^ As the reaction takes place under anodic conditions only, reductive activation of sulfur seems not to be absolutely necessary. However, polysulfides were formed preferably in an undivided cell in a much higher extent as in a quasidivided cell^[13]^ with a platinum wire as anode (Table 1, entry 20). When a solution of sulfur only was electrolyzed, brownish streaks were observed at the cathode. According to these observations, we propose that the activation of elemental sulfur at the cathode takes place under formation of radical anion **C**. Then, recombination of the thiol‐radical and the sulfur‐radical in **D** may be possible in solution under formation of intermediate **E**. In the next step, we propose the sulfur‐sulfur bond formation of **E** with **B** to one sulfur atom within the sulfur‐chain in **F**. This step determines the length of the latter polysulfide chain, as all sulfur atoms between the organic substituents will be included in the polysulfide. The elemental sulfur‐side chain, attached to the polysulfide‐structure, donates an electron pair and eliminates under reversible formation of the desired polysulfide.

We assume that this reaction runs in a dynamic equilibrium that is reached after 4.0 *F* applied current. As shown in Figure 2, polysulfides of all sulfur chain lengths were formed from beginning of the reaction. After 4.0 *F*, the conversion of the disulfide reached its maximum causing a stagnancy in the sulfur conversion and the polysulfide formation. But, according to the previous study,^[11]^ we assume a permanent electrochemical S−S polysulfide‐bond metathesis. At the beginning of the reaction, splitting of a disulfide is highly probable. But the larger polysulfide quantities are formed, the higher the probability of a S−S‐bond cleavage from a higher sulfide. In theory, catalytic amounts of current are sufficient, because no changes in oxidation states take place at the disulfide and sulfur. However, in practice further electrolysis is necessary due to the constant S−S‐bond metathesis of already formed polysulfides. Finally, after 4.0 *F*, the disulfide conversion reaches its maximum so that further electrolysis only causes the S−S‐bond cleavage and recombination of polysulfides, whose dynamic equilibrium is already reached.

Unfortunately, we were not able to pin down the solvent effect of CS_2_ in this reaction because no useful results were be obtained from cyclic voltammograms with carbon disulfide as a co‐solvent (see the Supporting Information).

## Conclusion

In this article, the first electrochemical synthesis of polysulfides (up to undecasulfides) from disulfides or thiols with elemental sulfur has been introduced. During the optimization, the reaction proved to be very robust, as it can be performed in different solvents with a variety of supporting electrolytes and electrodes under a wide range of reaction conditions. Moreover, the reaction is highly tolerant of the sulfur atom‐equivalents added, as excess sulfur simply remains in solution to a certain degree. The reaction does not need any catalyst and can be performed in air with low‐priced materials, which makes it highly economic.

We observed an interesting solvent effect that affected the polysulfide distribution when CS_2_ was added as co‐solvent. To describe the change of the polysulfide distribution, we introduced the term “absorbance average sulfur amount in polysulfides at 248 nm” (SAP_248_), analogous to the “number average molar mass” used in polymer chemistry [Eq. (1)]. Additionally, we calculated the dispersity, *Đ*, of the polysulfide mixture. It turned out that the addition of CS_2_ gave a significantly narrower polysulfide distribution. The preferred emerging species can be adjusted by the volume fraction of added CS_2_ to some extent (Table 2, Figure 3).

We therefore established two different reaction setups for the investigation of different substrates, without any CS_2_ (setup **A**) and with 10 % (vol.) CS_2_ (setup **B**). Aliphatic disulfides responded well to both setups. However, setup **B** increased the conversion of the disulfides to >80 %. In addition, the yield of the preferably formed sulfide (di‐*n*‐butyl trisulfide (**1 b**)) rose to >40 %. The conversion of the aliphatic thiol **6** was nearly quantitative and gave 45 % of the trisulfide **6 b** with reaction setup **B**. Aromatic disulfides showed, in general, a lower conversion than aliphatic disulfides (30–60 %). Unfortunately, their conversion and polysulfide distribution could not be affected by the addition of CS_2_. In addition to the synthesis of symmetrical polysulfides, we proved that the synthesis of asymmetrical polysulfides from two symmetrical disulfides or a disulfide and a thiol was also possible.

Overall, this electrochemical reaction widens the field of organic polysulfide synthesis and is a useful addition to the expensive, but fast, rhodium‐catalyzed synthesis reported by yamaguchi.^[9]^ This work demonstrates a new way of sulfur activation and lays the foundation for potential further work in this research field. As a direct application, dynamic polysulfide libraries with many products can be created from a single reaction by the use of two or more symmetrical disulfides or thiols as starting materials. We found an interesting effect of co‐solvent CS_2_ on the polysulfide distribution that raises new questions for mechanistic investigations and might be useful for lithium‐sulfur battery research.

## Experimental Section

**General procedure for the electrochemical polysulfide synthesis**: An undivided cell was charged with elemental sulfur (0.0625 mmol, 2.0 equiv. [S]), organic disulfide (0.25 mmol, 1.0 equiv.) or thiol (0.5 mmol, 2.0 equiv.) and tetrabutylammonium tetrafluoroborate (1.0 mmol) in dichloromethane (setup **A**=10 mL, setup **B**=9 mL+1 mL CS_2_). After complete dissolution of the sulfur, the electrolysis at glassy carbon electrodes (1.5 cm^2^) was performed at 10 mA constant current until 4.0 *F* were passed through the solution (160 min). The reaction mixture was filtered through aluminium oxide (neutral), with dichloromethane as solvent. Then, benzophenone was added as internal standard for the determination of the polysulfide distribution in quantitative ^13^C NMR or HPLC‐UV analysis. The solvent was removed under reduced pressure in a fume hood (due to the possibility of CS_2_ being given off) to obtain the pure polysulfide mixture.

## Conflict of interest

The authors declare no conflict of interest.

## Supporting information

As a service to our authors and readers, this journal provides supporting information supplied by the authors. Such materials are peer reviewed and may be re‐organized for online delivery, but are not copy‐edited or typeset. Technical support issues arising from supporting information (other than missing files) should be addressed to the authors.

Supporting Information
