# Supplementary material for: Electrochemical Synthesis of Organic Polysulfides from Disulfides by Sulfur Insertion from S8 and an Unexpected Solvent Effect on the Product Distribution
Source: Chemistry. 2021 Jun 1;27(43):11141–9. doi: 10.1002/chem.202101023 (PMC8453886; doi:10.1002/chem.202101023)
Supplement: Supplementary file 1 — Supporting Information [file CHEM-27-11141-s001.pdf]

Chemistry–A European Journal

Supporting Information

Electrochemical Synthesis of Organic Polysulfides from Disulfides by Sulfur Insertion from S_8 and an Unexpected Solvent Effect on the Product Distribution

Jan Fährmann and Gerhard Hilt*

Content

1 General information 1
2 Experimental Section 3
2.1 Spectroscopic data of synthesized compounds 3
2.2 NMR spectra 7
2.2.1 Disulfides and starting materials 7
2.2.2 Polysulfides 11
2.2.3 Quantitative ^{13}C NMR spectra 17
2.3 HPLC-UV Chromatogramms 22
2.3.1 Disulfides and starting materials 22
2.3.2 Polysulfides 26
2.4 UV-Vis spectra 33
2.4.1 Disulfides and starting material 33
2.5 GC-MS chromatogramms of mixed polysulfides 37
2.6 Cyclic Voltammetry 38
3 Calculation of sulfur amount in polysulfides (SAP_{248}) and dispersity (*Đ*) 40
3.1 Definition of SAP248 and dispersity Đ 40
3.2 Original Data for SAP and *Đ*-calculation 41
3.3 Colorized graphs for the polysulfide distributions 42

1 General information

All reactions were carried out under atmospheric conditions. The used solvents were purchased in high purity (HPLC grade). Commercially available chemicals were used without further purification. Electrolysis were executed in undivided under constant current (drawing/photo see Figure S1). Therefore, a laboratory power supply from *Aim TTi MX100T Triple Output Multi-Range DC Power Supply* (35 V, 3 A) was used. As electrode materials platinum plates (dimensions 35 mm x 10 mm x 0.5 mm, purity min. 99.95%), glassy carbon plates (dimensions 35 mm x 10 mm x 0.5 mm), carbon roving (6 k, 400 tex), copper (Cu-DHP according to ENCW024A, dimensions 35 mm x 10 mm x 0.5 mm) and stainless steel (material number 1.4571 according to EN10027-2, dimensions 35 mm x 10 mm x 0.5 mm) were utilised.

NMR spectra were recorded on a *Bruker Avance III* spectrometer utilising pre-set pulse programs. The measurements were performed at room temperature. The chemical shifts are given in parts per million (ppm). Calibration was done by referring to the residual solvent signal ($CDCl_3$: ^{1}H NMR 7.26 ppm, ^{13}C NMR 77.16 ppm) in relation to tetramethylsilane. Quantitative ^{13}C NMR spectra were measured with a relaxation time of 30 s per scan.

HPLC-UV spectra were recorded on an *Agilent 1220 infinity II* with a reverse phase (EC-C18) column *poroshell 120* (4.6 mm x 150 mm, 2.7 µm particle size). A UV-Vis detector was used at a wavelength of 248 nm. As mobile phase, methanol at a flow rate of 0.5 mL/min was used.

HR-MS (High resolution mass spectra) were recorded on a *Thermo Scientific DFS spectrometer* using electron ionization (EI) at an energy of 40 eV.

GC-MS measurements were performed on a *Shimadzu GCMS-QP2020*. As capillary column an *Optima 5 HT* from *Macherey-Nagel* was used (length 30 m, inner diameter 0.25 mm, film thickness 0.25 µm). The ionisation was accomplished by electron impact (EI) with an energy of 70 eV.

GC-FID measurements were carried out on a *Shimadzu GC-2010 Plus* gas chromatograph with an *Optima 5 MS* capillary column from *Macherey-Nagel* (length 15 m, inner diameter 0.25 mm, film thickness 0.25 µm).

CV (Cyclic voltammetry) measurements were carried out on a *BAS C3* cell stand and a *BAS 100* electrochemical analyzer using a glassy carbon disk working electrode (2.0 mm diameter) and platinum wire counter electrode (0.5 mm diameter). Potentials were referred to a saturated Ag/AgCl (3 M NaCl) reference electrode.

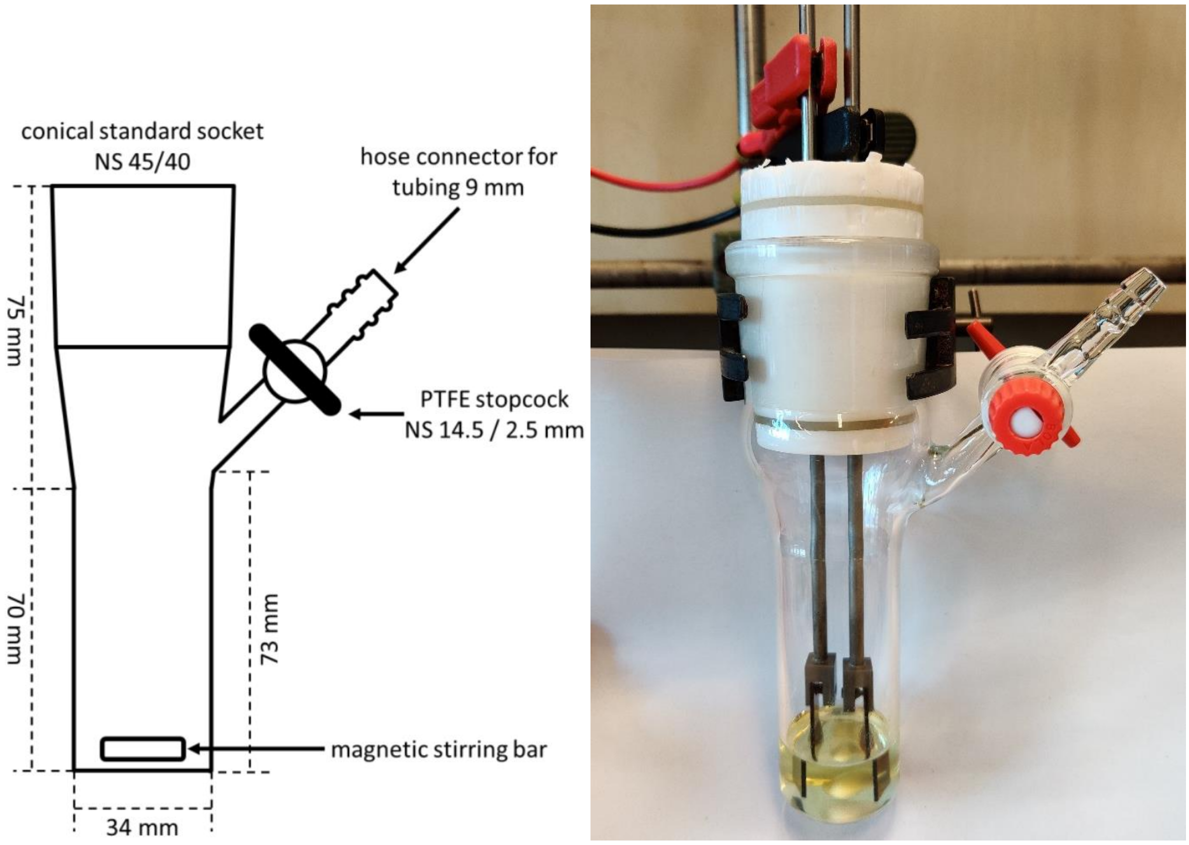

Figure S1: (left) Sketch of the undivided cells that were used. (right) Photo of the electrochemical cell-setup.

2 Experimental Section

2.1 Spectroscopic data of synthesized compounds

Di-*n*-butyl polysulfides (1b–1j)

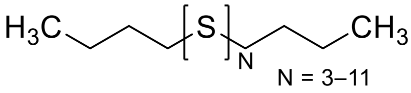

1b–1j

^{1}H NMR (500 MHz, $CDCl_3$): δ = 0.89–0.99 (m, 6H), 1.38–1.51 (m, 4H), 1.62–1.80 (m, 4H), 2.67–3.03 (m, 4H) ppm.

^{13}C NMR (126 MHz, $CDCl_3$): δ = 13.8 (m, 2C), 21.7–21.8 (m, 2C), 31.1–31.5 (m, 2C), 38.7–39.8 (m, 2C) ppm.

HR-MS (EI, 40 eV):
m/z calcd for $C_8H_{18}S_2$ (**1b**) 178.0844, found 178.0844;
m/z calcd for $C_8H_{18}S_3$ (**1c**) 210.0565, found 210.0574;
m/z calcd for $C_8H_{18}S_4$ (**1d**) 242.0286, found 242.0296;
m/z calcd for $C_8H_{18}S_5$ (**1e**) 274.0007, found 273.9996;
m/z calcd for $C_8H_{18}S_6$ (**1f**) 305.9719, found 305.9727;
m/z calcd for $C_8H_{18}S_7$ (**1g**) 337.9448, found 337.9454;
m/z calcd for $C_8H_{18}S_8$ (**1h**) 369.9169, found 369.9168;
m/z calcd for $C_8H_{18}S_9$: (**1i**) 401.8889, found 401.8880.

HPLC-UV (methanol, 0.5 mL/min, 248 nm) ret. time = 5.56 (**1b**), 6.06 (**1c**), 7.00 (**1d**), 8.19 (**1e**), 9.78 (**1f**), 11.81 (**1g**), 14.51 (**1h**), 17.93 (**1i**), 22.33 (**1j**) min.

Dicyclohexyl polysulfides (2b–2f)

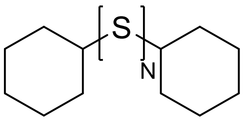

2b–2f N = 3–7

^{1}H NMR (500 MHz, $CDCl_3$): δ = 1.20–1.51 (m, 10H), 1.57–67 (m, 2H), 1.75–1.86 (m, 4H), 2.01–2.16 (m, 4H), 2.64–3.15 (m, 2H) ppm.

^{13}C NMR (126 MHz, $CDCl_3$): δ = 25.7–26.2 (m, 6C), 32.8–33.0 (m, 4C), 50.1–50.9 (m, 2C) ppm.

HR-MS (EI, 40 eV):
m/z calcd for $C_{12}H_{22}S_3$ (**2b**) 262.0878, found 262.0883;
m/z calcd for $C_{12}H_{22}S_4$ (**2c**) 294.0599, found 294.0602;
m/z calcd for $C_{12}H_{22}S_5$ (**2d**) 326.0320, found 326.0330;

m/z calcd for $C_{12}H_{22}S_6$ (**2e**) 358.0040, found 358.0038;
m/z calcd for $C_{12}H_{22}S_7$ (**2f**) 389.9761, found 389.9770;
m/z calcd for $C_{12}H_{22}S_8$ (**2g**) 421.9482, found 421.9489.

HPLC-UV (methanol, 0.5 mL/min, 248 nm) ret. time = 8.94 (**2b**), 10.69 (**2c**), 13.00 (**2d**), 16.18 (**2e**), 20.10 (**2f**) min.

Diphenyl polysulfides (3b–3d)

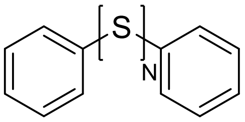

3b–3d N = 3–5

¹H NMR (500 MHz, $CDCl_3$): δ = 7.11–7.24 (m, 6H), 7.39–7.47 (m, 4H) ppm.

¹³C NMR (126 MHz, $CDCl_3$): δ = 127.3–130.6 (m, 10C), 136.5–137.2 (m, 2C) ppm.

HR-MS (EI, 40 eV):
m/z calcd for $C_{12}H_9S_3$ (**3b**-H) 249.9939, found 249.9939;
m/z calcd for $C_{12}H_9S_4$ (**3c**-H) 281.9660, found 281.9652;
m/z calcd for $C_{12}H_9S_5$ (**3d**-H) 313.9381, found 313.9387.

HPLC-UV (methanol, 0.5 mL/min, 248 nm) ret. time = 4.81 (**3b**), 5.36 (**3c**), 5.98 (**3d**) min.

Di-*p*-tolyl polysulfides (4b–4g)

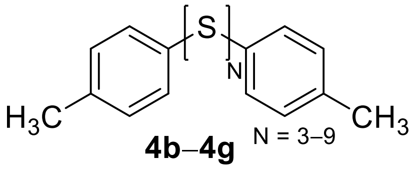

4b–4g N = 3–9

¹H NMR (500 MHz, $CDCl_3$): δ = 2.13–2.43 (m, 6H), 7.09–7.23 (m, 4H), 7.37–7.58 (m, 4H) ppm.

¹³C NMR (126 MHz, $CDCl_3$): δ = 21.2–21.4 (m, 2C), 128.5–131.5 (m, 8C), 132.7–134.23 (m, 2H), 137.4–139.4 (m, 2C) ppm.

HR-MS (EI, 40 eV):
m/z calcd for $C_{14}H_{14}S_3$ (**4b**) 278.0252, found 278.0257;
m/z calcd for $C_{14}H_{14}S_4$ (**4c**) 309.9973, found 309.9978;
m/z calcd for $C_{14}H_{14}S_5$ (**4d**) 341.9694, found 341.9698;
m/z calcd for $C_{14}H_{14}S_6$ (**4e**) 373.9414, found 373.9416.

HPLC-UV (methanol, 0.5 mL/min, 248 nm) ret. time = 5.72 (**4b**), 6.53 (**4c**), 7.45 (**4d**), 8.89 (**4e**), 10.64 (**4f**), 12.96 (**4g**) min.

Di-*p*-methoxyphenyl polysulfides (5b–5f)

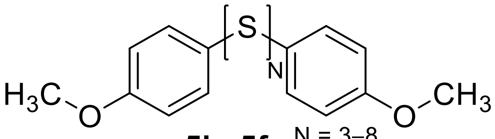

5b–5f N = 3–8

^{1}H NMR (500 MHz, $CDCl_3$): δ = 3.76–3.86 (m, 6H), 6.77–6.94 (m, 4H), 7.36–7.61 (m, 4H) ppm.

^{13}C NMR (126 MHz, $CDCl_3$): δ = 55.4 (2C), 114.8–115.0 (m, 4C), 127.0–128.6 (m, 2C), 132.7–134.5 (m, 4C), 160.0–161.0 (m, 2C) ppm.

HR-MS (EI, 40 eV):
m/z calcd for $C_{14}H_{14}O_2S_3$ (**5b**) 310.0150, found 310.0140;
m/z calcd for $C_{14}H_{14}O_2S_4$ (**5c**) 341.9871, found 341.9864;
m/z calcd for $C_{14}H_{14}O_2S_5$ (**5d**) 373.9592, found 373.9590;
m/z calcd for $C_{14}H_{14}O_2S_6$ (**5e**) 405.9313, found 405.9317.

HPLC-UV (methanol, 0.5 mL/min, 248 nm) ret. time = 4.52 (**5b**), 4.96 (**5c**), 5.51 (**5d**), 6.34 (**5e**), 7.25 (**5f**) min.

Di-*n*-dodecyl polysulfides (6a–6f)

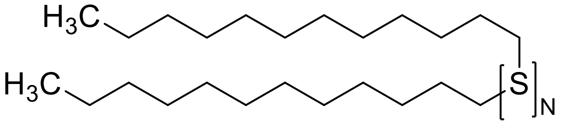

6a–6f N = 2–8

^{1}H NMR (500 MHz, $CDCl_3$): δ = 0.89 (t, *J* = 6.8 Hz, 6H), 1.21–1.35 (m, 32H), 1.35–1.46 (m, 4H), 1.62–1.81 (m, 4H), 2.64–3.02 (m, 4H) ppm.

^{13}C NMR (126 MHz, $CDCl_3$): δ = 14.3 (2C), 22.8 (2C), 32.0 (2C),28.6–29.8 (m, 16H), 39.1–40.2 (2C) ppm.

HR-MS (EI, 40 eV):
m/z calcd for $C_{24}H_{50}S_2$ (**6a**) 402.3348, found 402.3353;
m/z calcd for $C_{24}H_{50}S_3$ (**6b**) 434.3069, found 434.3074;
m/z calcd for $C_{24}H_{50}S_4$ (**6c**) 466.2790, found 466.2800;
m/z calcd for $C_{24}H_{50}S_5$ (**6d**) 498.2511, found 498.2507;
m/z calcd for $C_{24}H_{50}S_6$ (**6e**) 530.2231, found 530.2238;

HPLC-UV (methanol, 0.5 mL/min, 248 nm) ret. time = 42.69 (**6a**), 54.67 (**6b**), 63.07 (**6c**), 80.41 (**6d**), 102.11 (**6e**), 131.32 (**6f**) min.

_n_-Butylcyclohexyl polysulfides (7a–7e) (mixture with 1a–1f and 2a–2f)

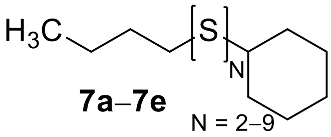

7a–7e

^{1}H NMR (500 MHz, $CDCl_3$): δ = 0.89–0.98 (m, 3H), 1.22–1.50 (m, 7H), 1.59–1.85 (m, 5H), 2.00–2.14 (m, 2H), 2.65–2.76 (m, 1H), 2.84–3.14 (m, 2H) ppm.

^{13}C NMR (126 MHz, $CDCl_3$): δ = 13.7–13.8 (m, 1C), 21.7–21.8 (m, 1C), 25.7–26.3 (m, 3C), 31.0–31.4 (m, 1C), 21.7–33.0 (m, 2C), 38.7–40.1 (m, 1C), 49.7–50.8 (m, 1C) ppm.

HR-MS (EI, 40 eV):
m/z calcd for $C_{10}H_{20}S_3$ (**7b**) 236.0722, found 236.0718;
m/z calcd for $C_{10}H_{20}S_4$ (**7c**) 268.0442, found 268.0440;
m/z calcd for $C_{10}H_{20}S_5$ (**7d**) 300.0163, found 300.0163;
m/z calcd for $C_{10}H_{20}S_6$ (**7e**) 331.9884, found 331.9890;
m/z calcd for $C_{10}H_{20}S_7$ (**7f**) 363.9604, found 363.9611;
m/z calcd for $C_{10}H_{20}S_8$ (**7g**) 395.9325, found 395.9319.

HPLC-UV (methanol, 0.5 mL/min, 248 nm) ret. time = 5.85 (**7a**), 7.43 (**7b**), 10.42 (**7c**), 12.70 (**7d**), 15.52 (**7e**) min.

Cyclohexyl-_n_-dodecyl polysulfides (8a–8f) (mixture with 1a–1f and 6a–6f)

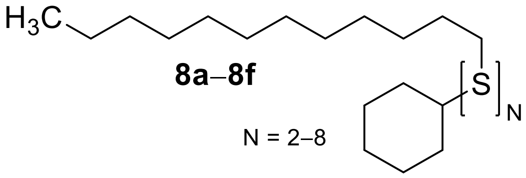

8a–8f

N = 2–8

^{1}H NMR (500 MHz, $CDCl_3$): δ = 0.88 (t, J = 6.8 Hz, 3H), 1.22–1.48 (m, 23H), 1.60–1.85 (m, 5H), 2.00–2.14 (m, 2H), 2.65–2.71 (m, 1H), 2.84–3.09 (m, 2H) ppm.

^{13}C NMR (126 MHz, $CDCl_3$): δ = 14.2 (1C), 22.8 (1C), 25.8–26.2 (m, 3C), 28.6–29.8 (m, 9C), 32.1–33.1 (2C), 39.1–40.5 (1C), 49.7–50.9 (1C) ppm.

HR-MS (EI, 40 eV):
m/z calcd for $C_{18}H_{36}S_2$ (**8a**) 316.2253, found 316.2252;
m/z calcd for $C_{18}H_{36}S_3$ (**8b**) 348.1974, found 348.1966;
m/z calcd for $C_{18}H_{36}S_4$ (**8c**) 380.1697, found 380.1699;
m/z calcd for $C_{18}H_{36}S_5$ (**8d**) 412.1415, found 412.1416;

HPLC-UV (methanol, 0.5 mL/min, 248 nm) ret. time = 15.72 (**8a**), 19.64 (**8b**), 22.19 (**8c**), 27.61 (**8d**), 34.76 (**8e**), 44.37 (**8f**) min.

2.2 NMR spectra

2.2.1 Disulfides and starting materials

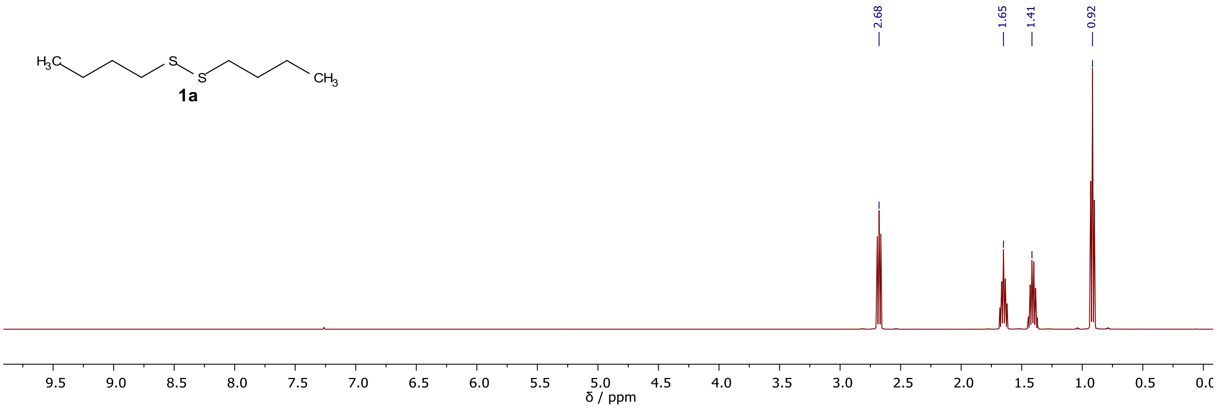

Figure S2: ^{1}H NMR of di-*n*-butyl disulfide (**1a**) in $CDCl_3$ at 500 MHz and rt.

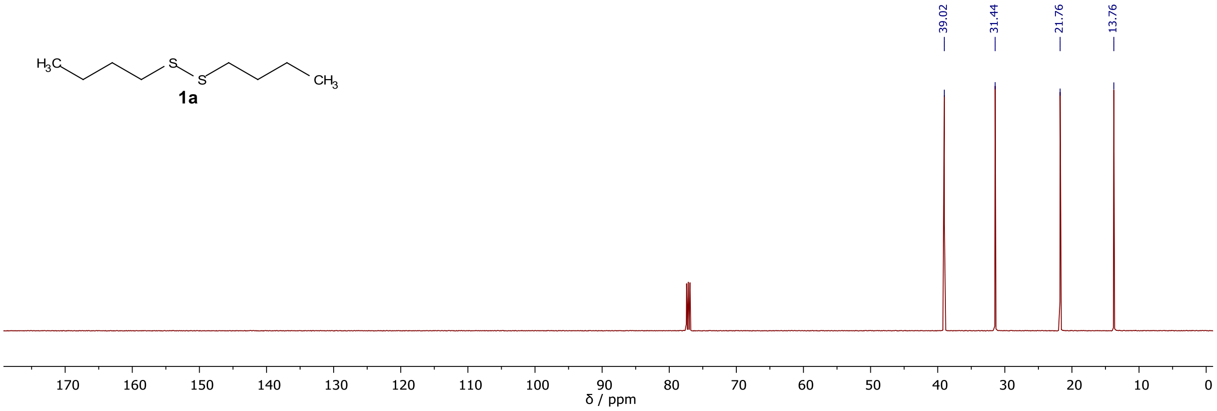

Figure S3: ^{13}C NMR of di-*n*-butyl disulfide (**1a**) in $CDCl_3$ at 126 MHz and rt.

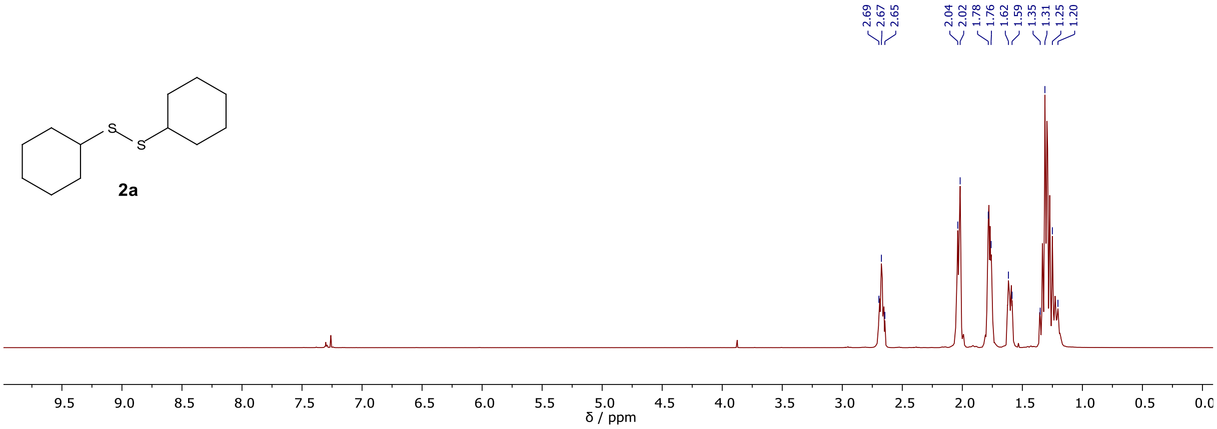

Figure S4: ^{1}H NMR of dicyclohexyl disulfide (**2a**) in $CDCl_3$ at 500 MHz and rt.

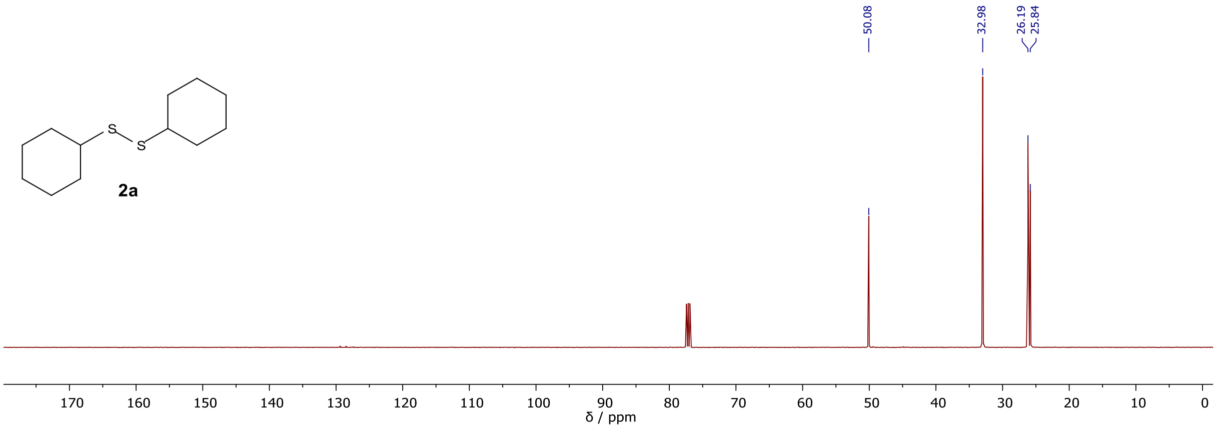

Figure S5: ^{13}C NMR of dicyclohexyl disulfide (**2a**) in $CDCl_3$ at 126 MHz and rt.

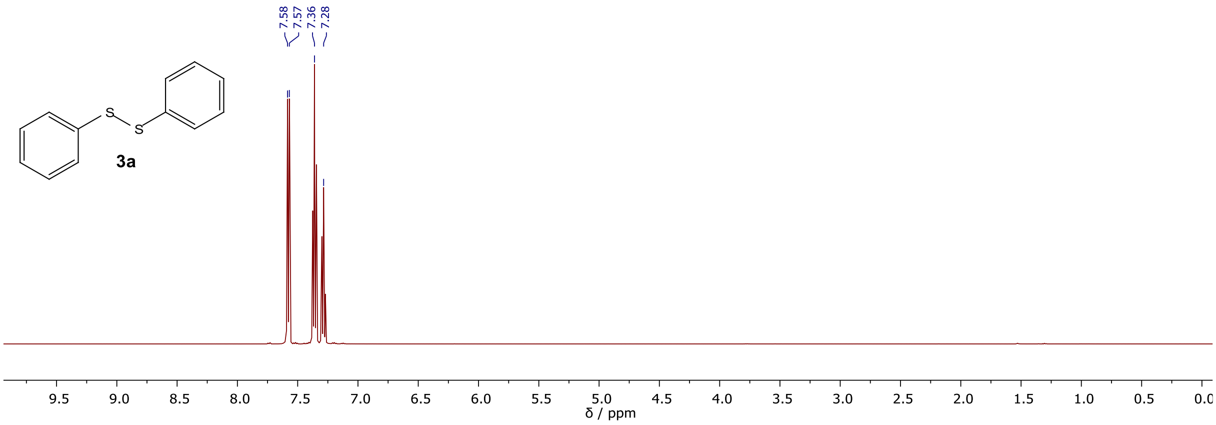

Figure S6: ^{1}H NMR of diphenyl disulfide (**3a**) in $CDCl_3$ at 500 MHz and rt.

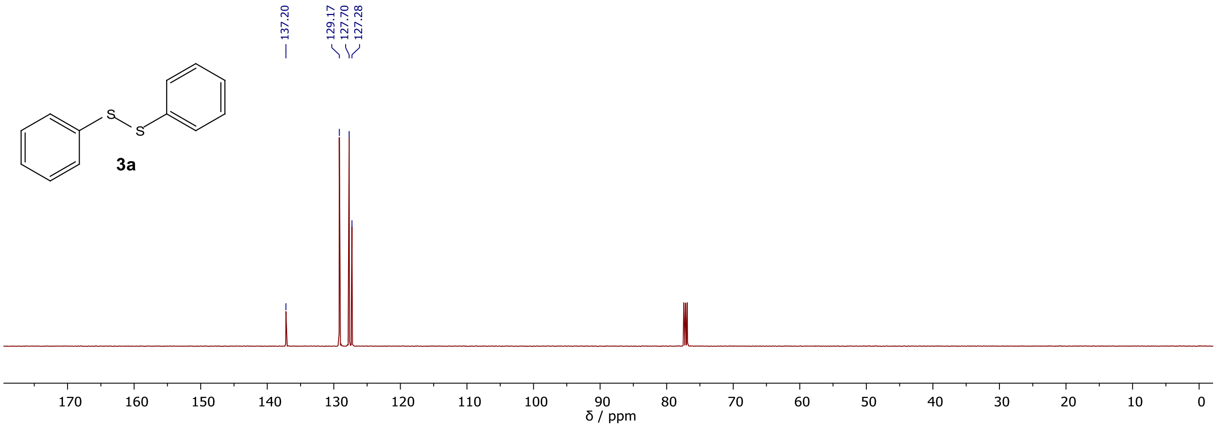

Figure S7: ^{13}C NMR of diphenyl disulfide (**3a**) in $CDCl_3$ at 126 MHz and rt.

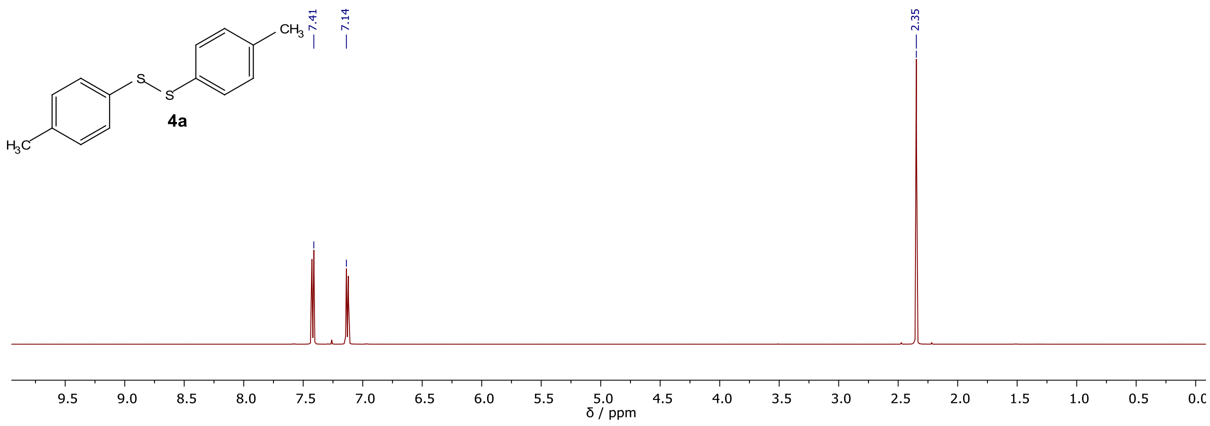

Figure S8: ^{1}H NMR of di-*p*-tolyl disulfide (**4a**) in $CDCl_3$ at 500 MHz and rt.

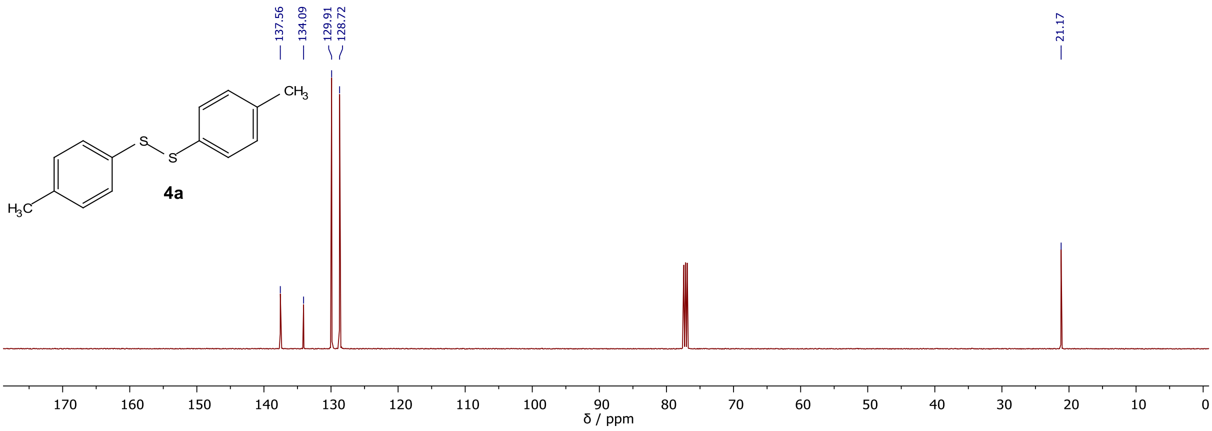

Figure S9: ^{13}C NMR of di-*p*-tolyl disulfide (**4a**) in $CDCl_3$ at 126 MHz and rt.

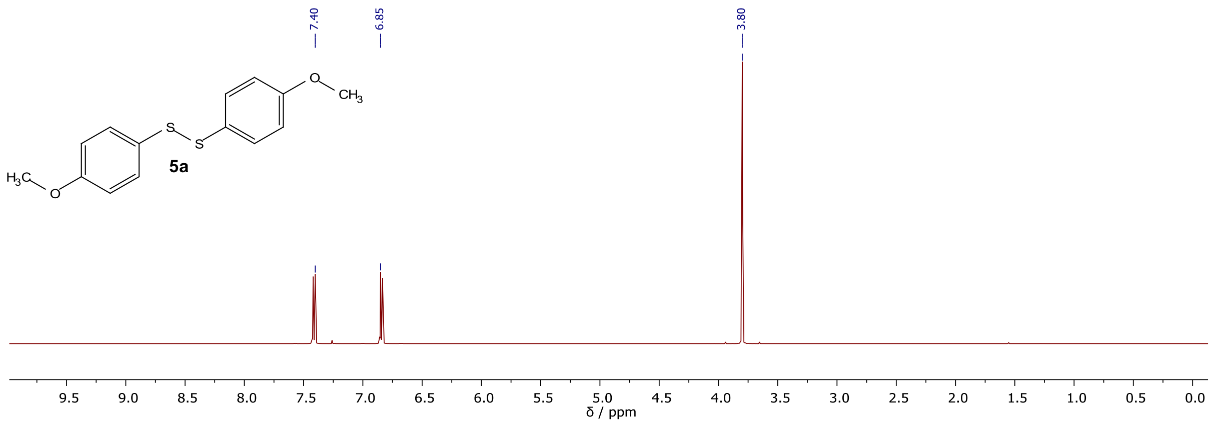

Figure S10: ^{1}H NMR of di-*p*-methoxyphenyl disulfide (**5a**) in $CDCl_3$ at 500 MHz and rt.

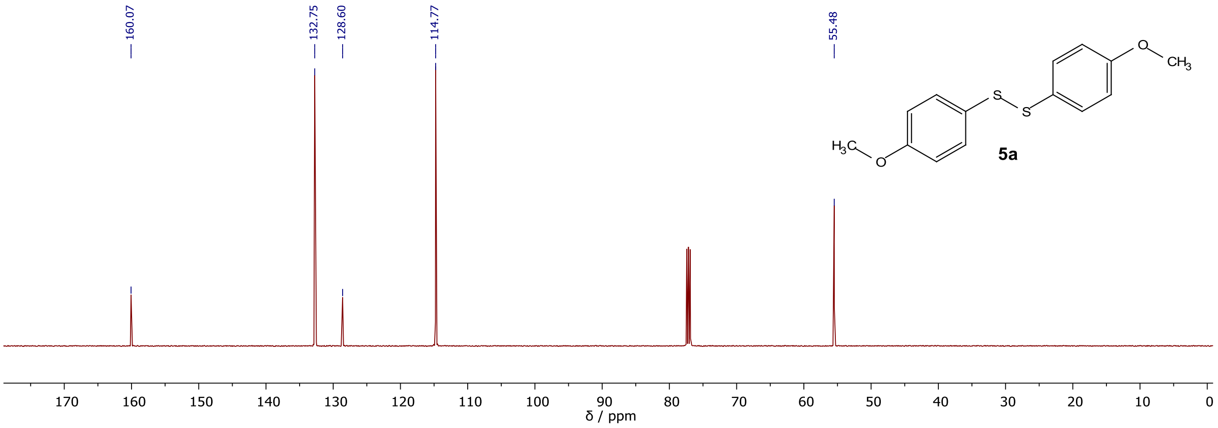

Figure S11: ^{13}C NMR of di-*p*-methoxyphenyl disulfide (**5a**) in $CDCl_3$ at 126 MHz and rt.

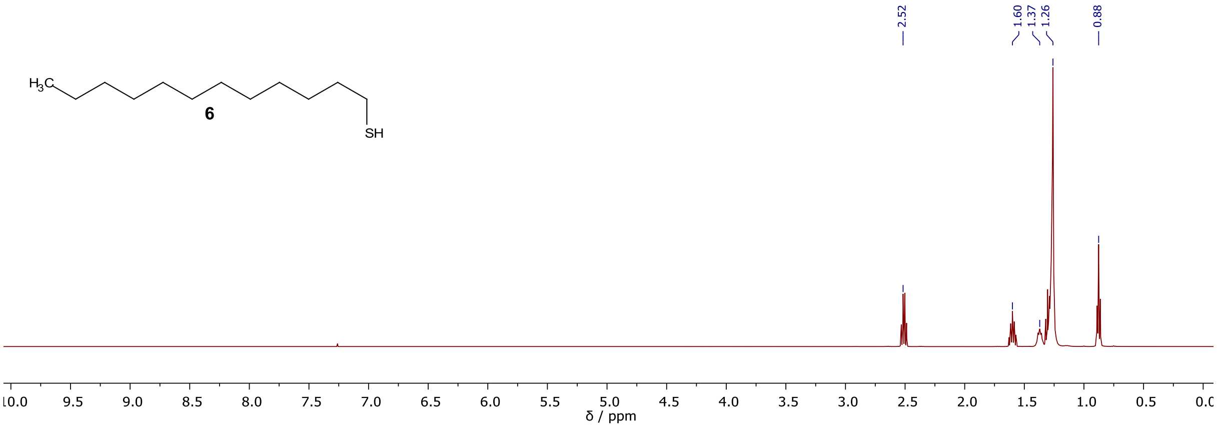

Figure S12: ^{1}H NMR of *n*-dodecylthiol (**6**) in $CDCl_3$ at 500 MHz and rt.

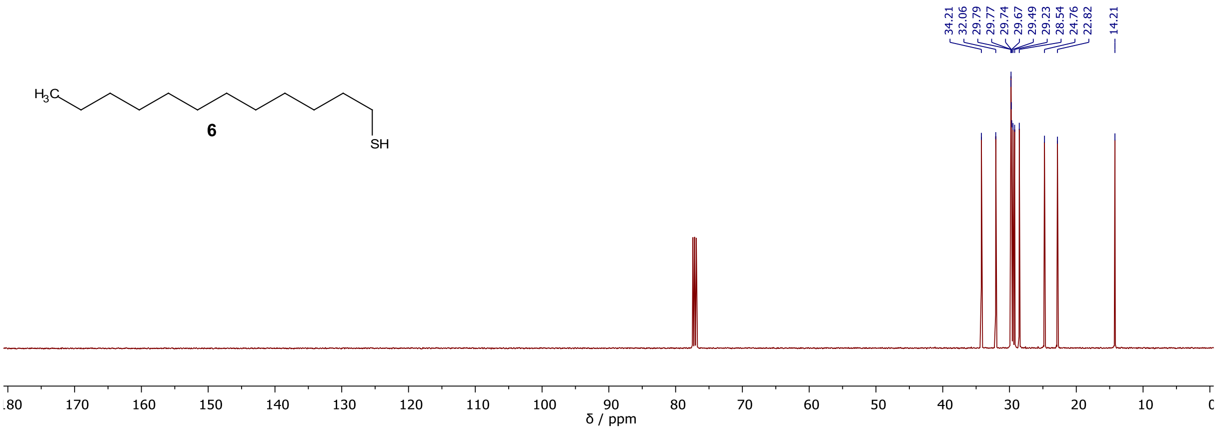

Figure S13: ^{13}C NMR of *n*-dodecylthiol (**6**) in $CDCl_3$ at 126 MHz and rt.

2.2.2 Polysulfides

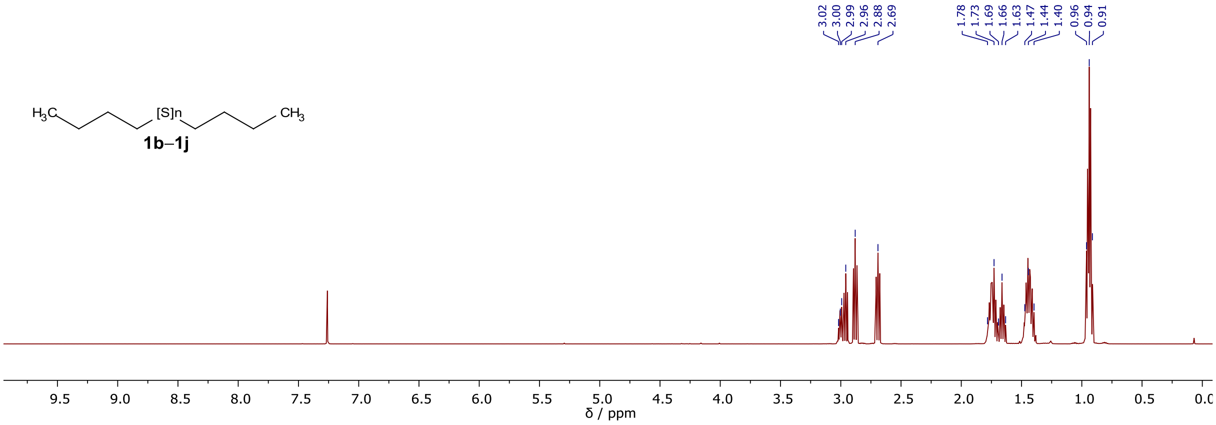

Figure S14: ^{1}H NMR of the di-*n*-butyl polysulfide mixture (**1b–1j**) in $CDCl_3$ at 500 MHz and rt.

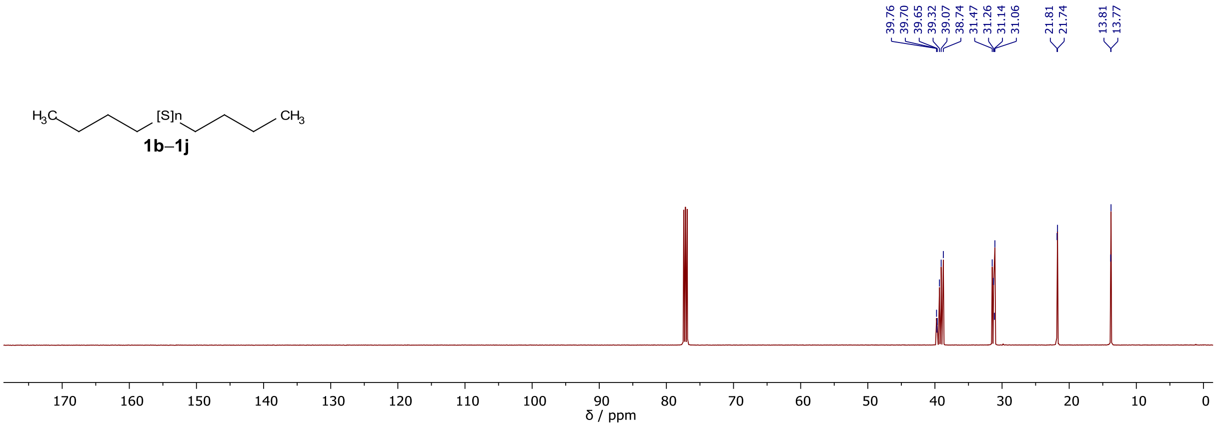

Figure S15: ^{13}C NMR of the di-*n*-butyl polysulfide mixture (**1b–1j**) in $CDCl_3$ at 126 MHz and rt.

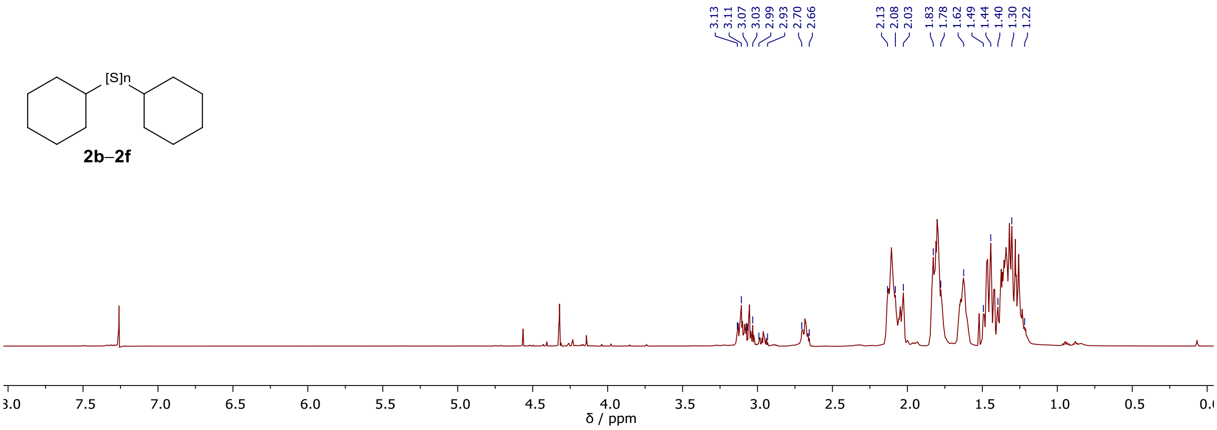

Figure S16: ^{1}H NMR of the dicyclohexyl polysulfide mixture (**2b–2f**) in $CDCl_3$ at 500 MHz and rt.

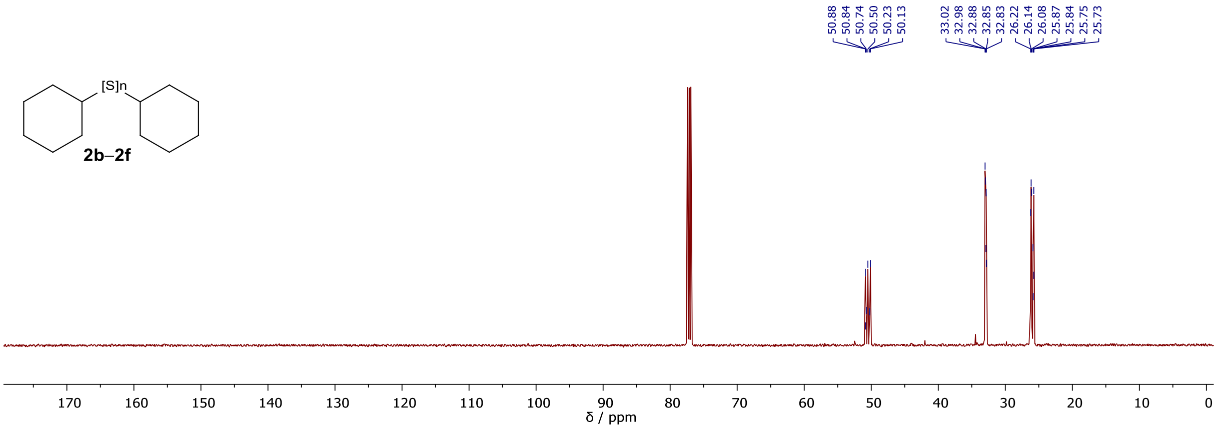

Figure S17: ^{13}C NMR of the dicyclohexyl polysulfide mixture (**2b**–**2f**) in $CDCl_3$ at 126 MHz and rt.

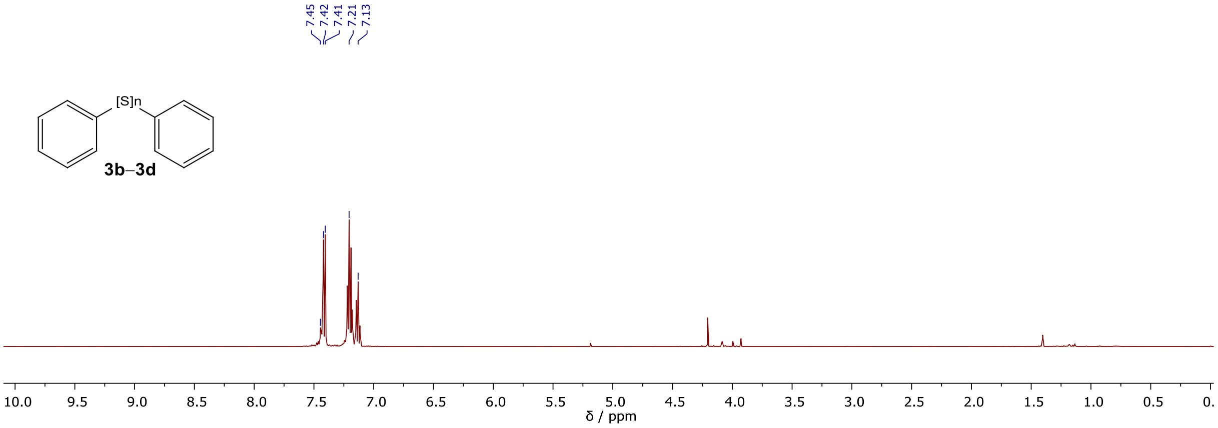

Figure S18: ^{1}H NMR of the diphenyl polysulfide mixture (**3b**–**3d**) in $CDCl_3$ at 500 MHz and rt.

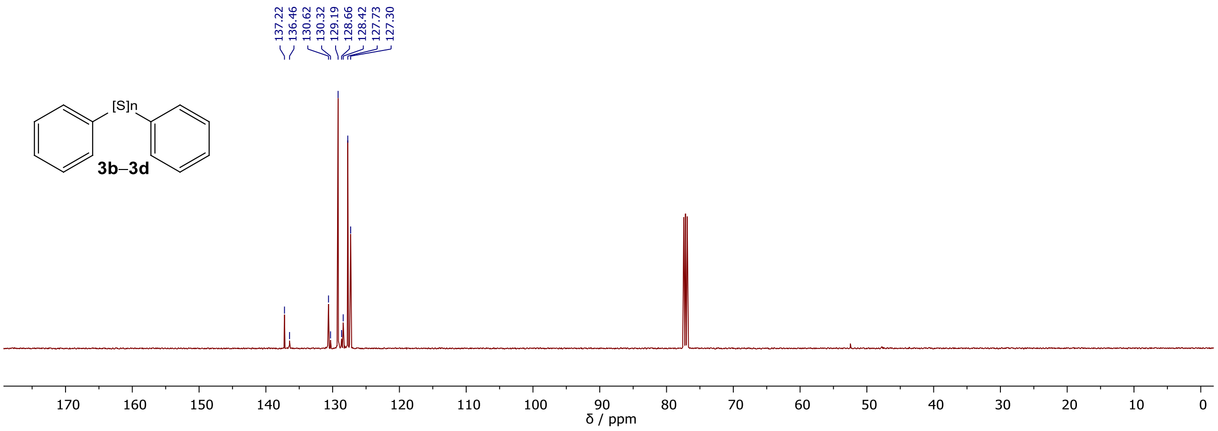

Figure S19: ^{13}C NMR of the diphenyl polysulfide mixture (**3b**–**3d**) in $CDCl_3$ at 126 MHz and rt.

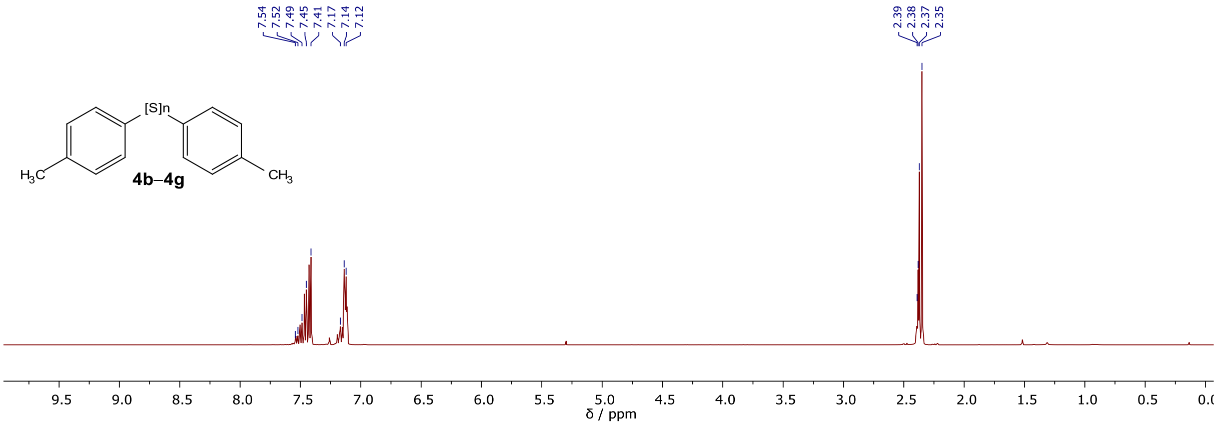

Figure S20: ^{1}H NMR of the di-*p*-tolyl polysulfide mixture (**4b–4g**) in $CDCl_3$ at 500 MHz and rt.

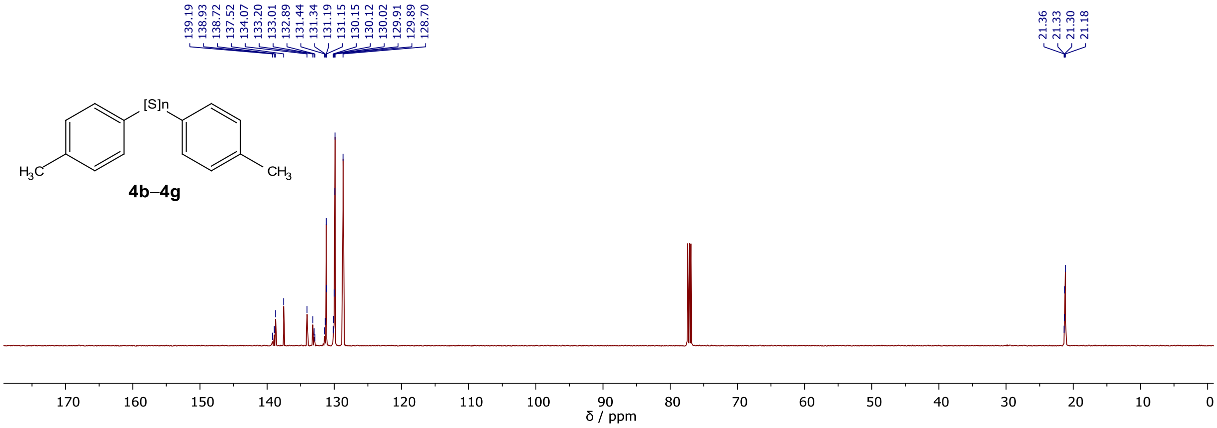

Figure S21: ^{13}C NMR of the di-*p*-tolyl polysulfide mixture (**4b–4g**) in $CDCl_3$ at 126 MHz and rt.

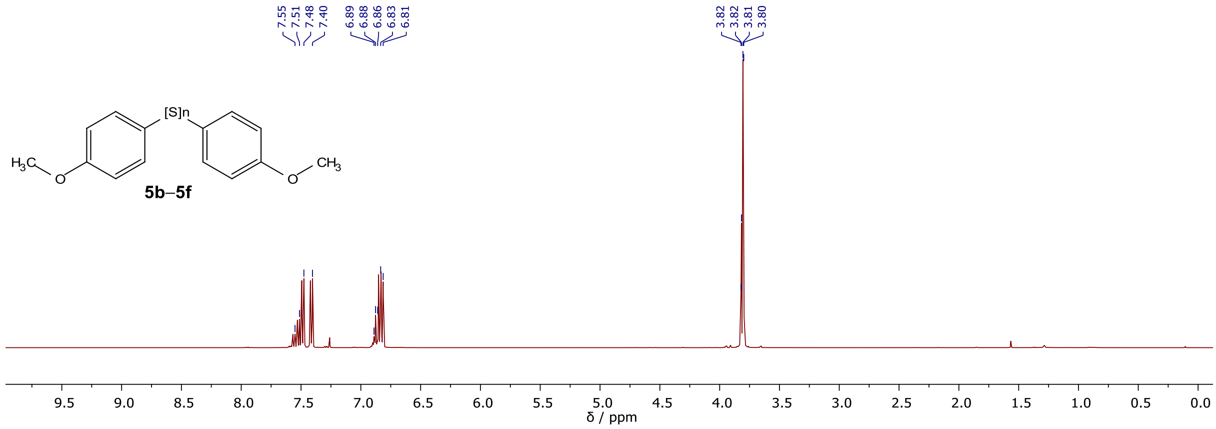

Figure S22: ^{1}H NMR of the di-*p*-methoxyphenyl polysulfide mixture (**5b–5f**) in $CDCl_3$ at 500 MHz and rt.

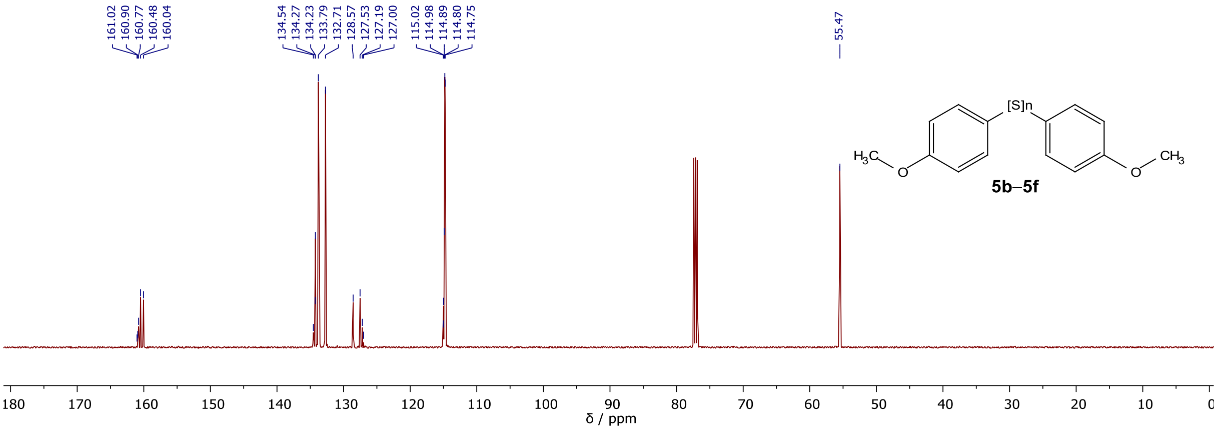

Figure S23: ^{13}C NMR of the di-*p*-methoxyphenyl polysulfide mixture (**5b**–**5f**) in $CDCl_3$ at 126 MHz and rt.

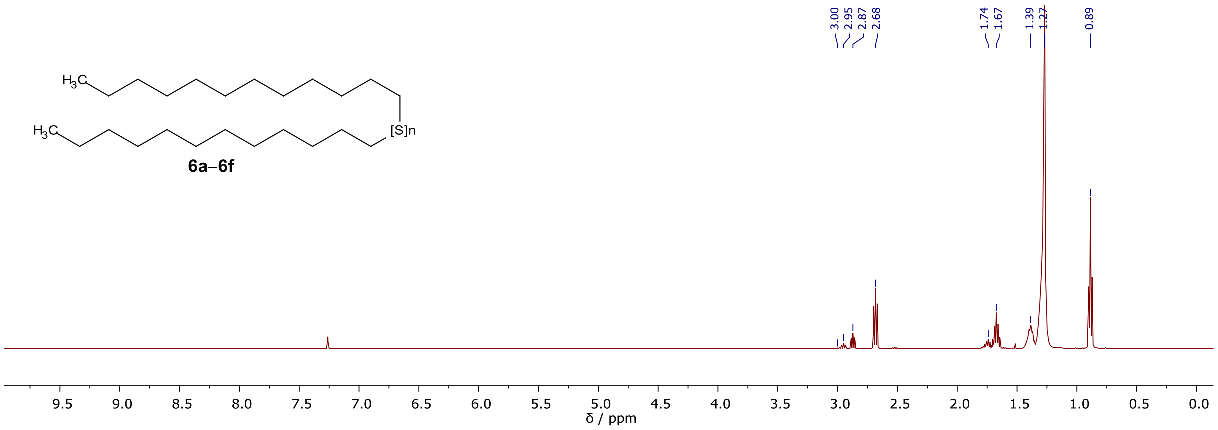

Figure S24: ^{1}H NMR of the di-*n*-dodecyl polysulfide mixture (**6a**–**6f**) in $CDCl_3$ at 500 MHz and rt.

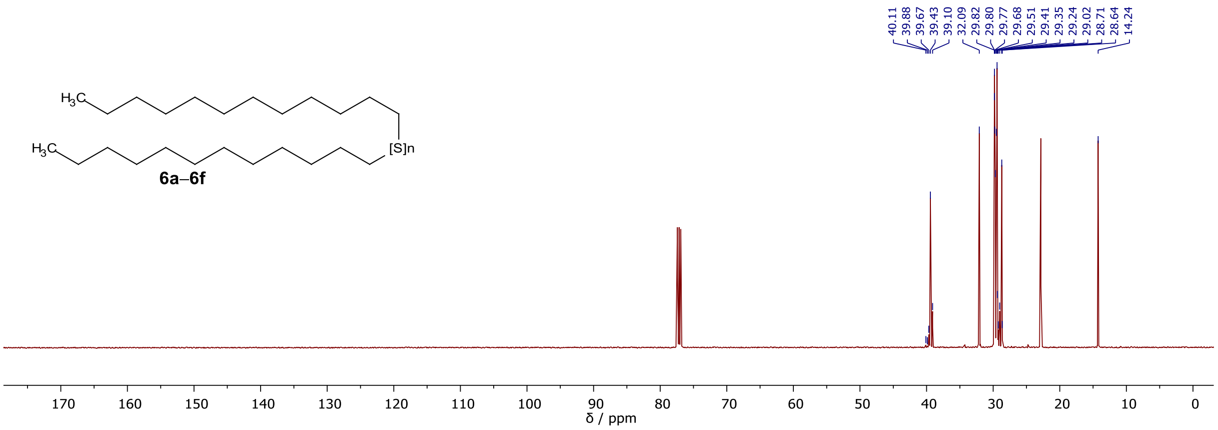

Figure S25: ^{13}C NMR of the di-*n*-dodecyl polysulfide mixture (**6a**–**6f**) in $CDCl_3$ at 126 MHz and rt.

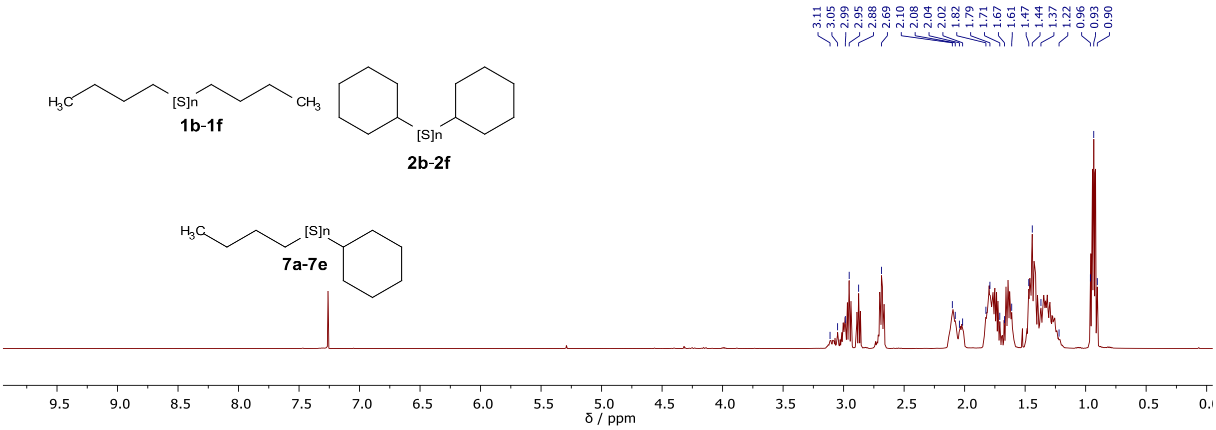

Figure S26: ^{1}H NMR of the di-*n*-butyl polysulfides (**1b**–**1f**), the dicyclohexyl polysulfides (**2b**–**2f**) and the *n*-butylcyclohexyl polysulfides (**7a**–**7e**) in $CDCl_3$ at 500 MHz and rt.

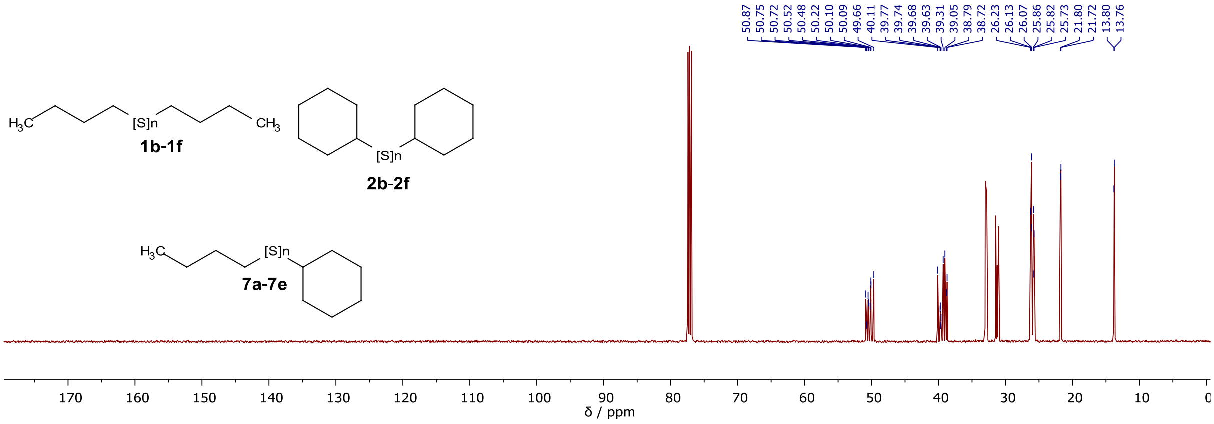

Figure S27: ^{13}C NMR of the di-*n*-butyl polysulfides (**1b**–**1f**), the dicyclohexyl polysulfides (**2b**–**2f**) and the *n*-butylcyclohexyl polysulfides (**7a**–**7e**) in $CDCl_3$ at 126 MHz and rt.

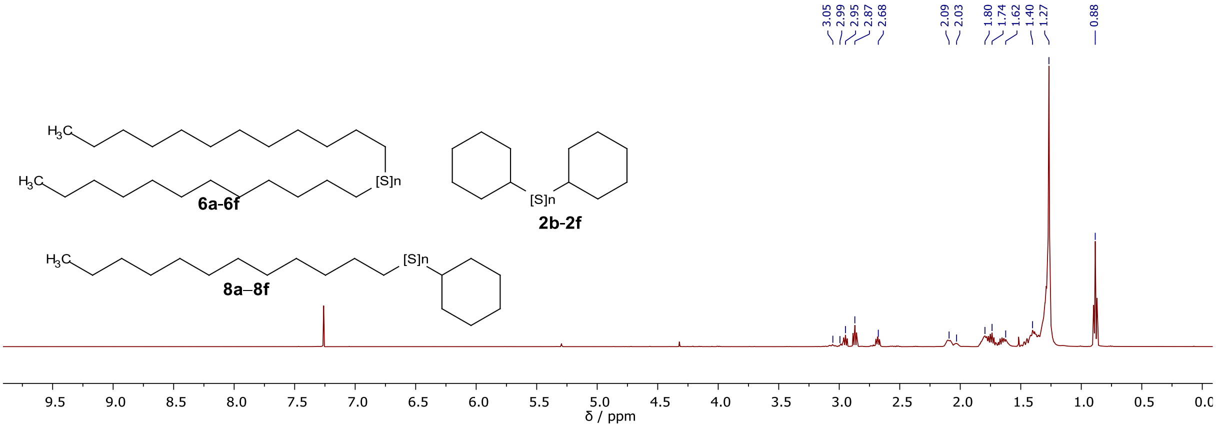

Figure S28: ^{1}H NMR of di-*n*-dodecyl polysulfides (**6b**–**6f**), the dicyclohexyl polysulfides (**2b**–**2f**) and the cyclohexyl-*n*-dodecyl polysulfides (**8a**–**8f**) in $CDCl_3$ at 500 MHz and rt.

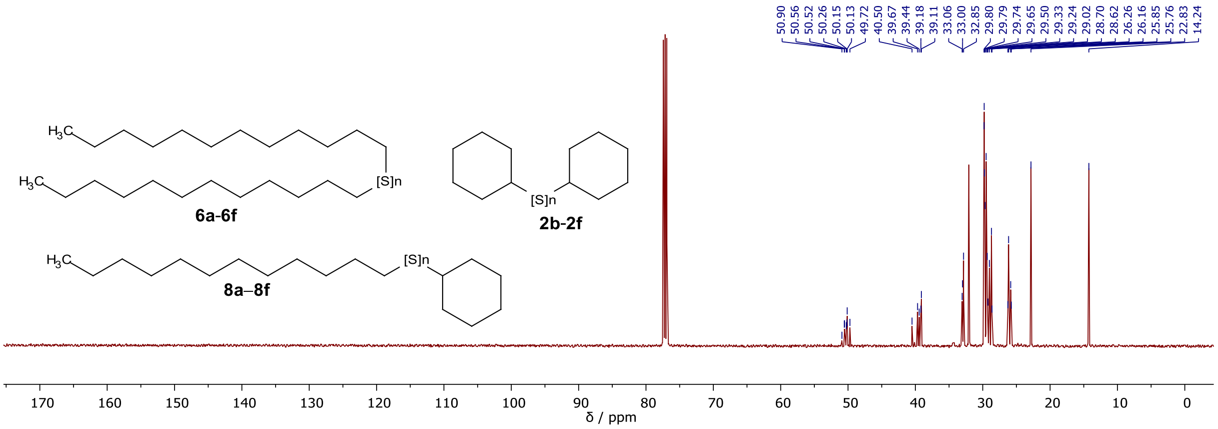

Figure S29: ^{13}C NMR of the di-*n*-dodecyl polysulfides (**6b**–**6f**), the dicyclohexyl polysulfides (**2b**–**2f**) and the cyclohexyl-*n*-dodecyl polysulfides (**8a**–**8f**) in $CDCl_3$ at 126 MHz and rt.

2.2.3 Quantitative ^{13}C NMR spectra

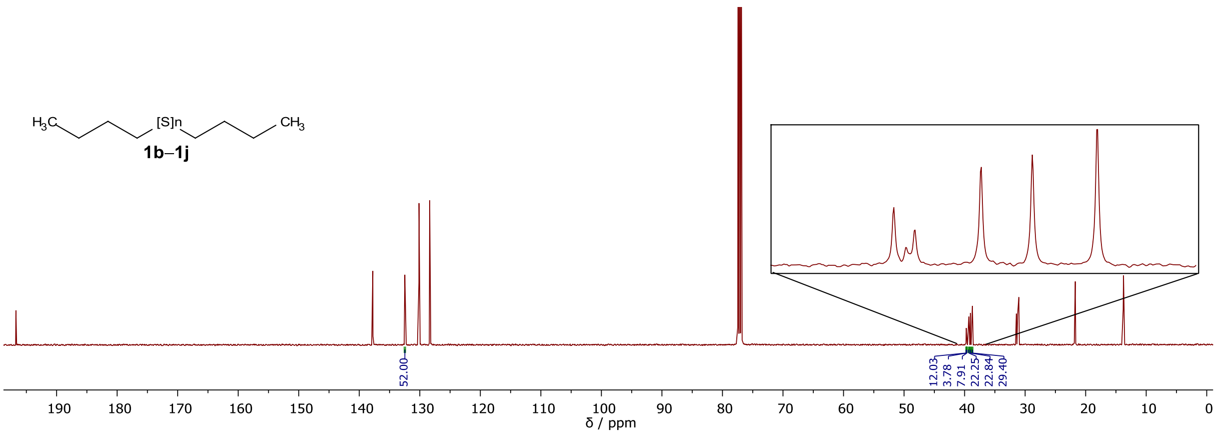

Figure S30: Quantitative ^{13}C NMR spectrum of the di-*n*-butyl polysulfides (**1b**–**1j**) from setup **A** (solvent DCM) at 126 MHz and rt with benzophenone as internal standard. The integral of benzophenone is set the way that the integral of each polysulfide represents its absolute yield.

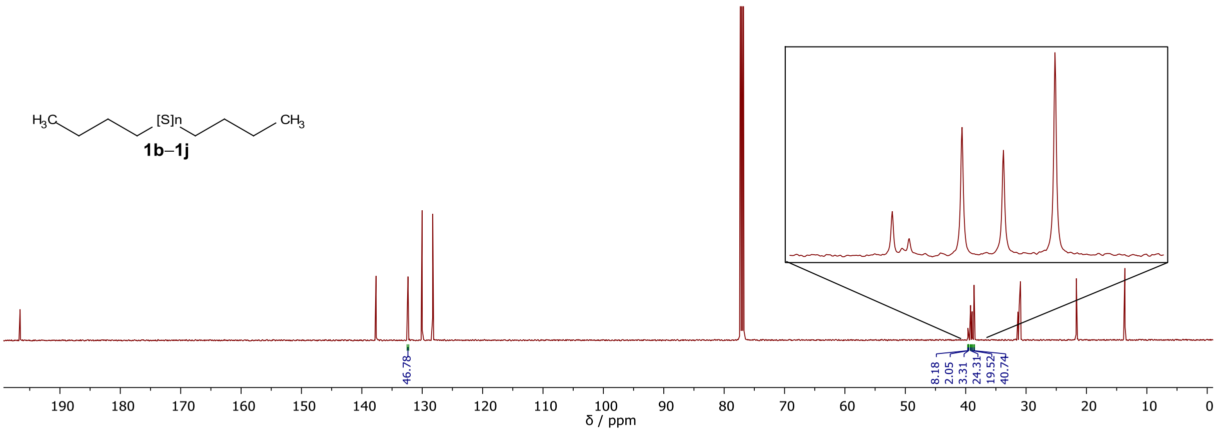

Figure S31: Quantitative ^{13}C NMR spectrum of the di-*n*-butyl polysulfides (**1b**–**1j**) from setup **B** (solvent DCM + 10% (vol.) CS_2) at 126 MHz and rt with benzophenone as internal standard. The integral of benzophenone is set the way that the integral of each polysulfide represents its absolute yield.

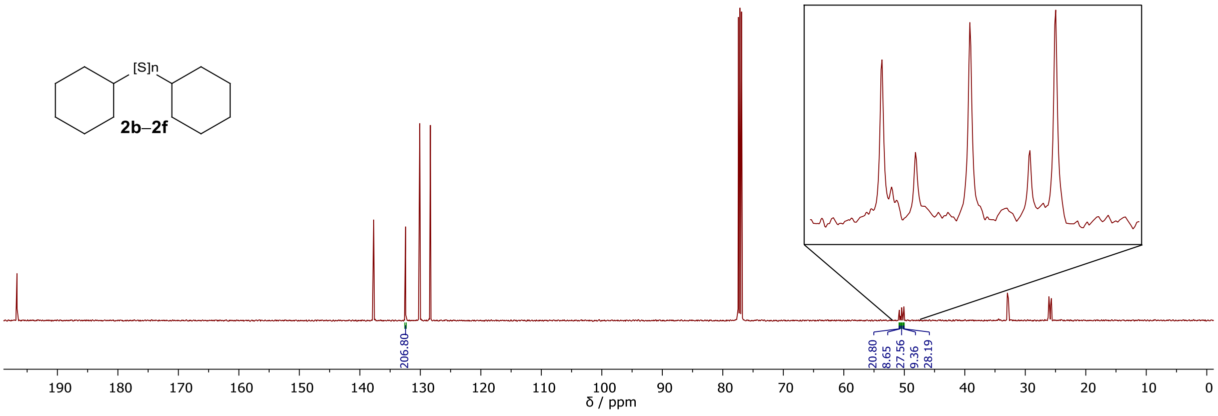

Figure S32: Quantitative ^{13}C NMR spectrum of the dicyclohexyl polysulfides (**2b**–**2f**) from setup **A** (solvent DCM) at 126 MHz and rt with benzophenone as internal standard. The integral of benzophenone is set the way that the integral of each polysulfide represents its absolute yield.

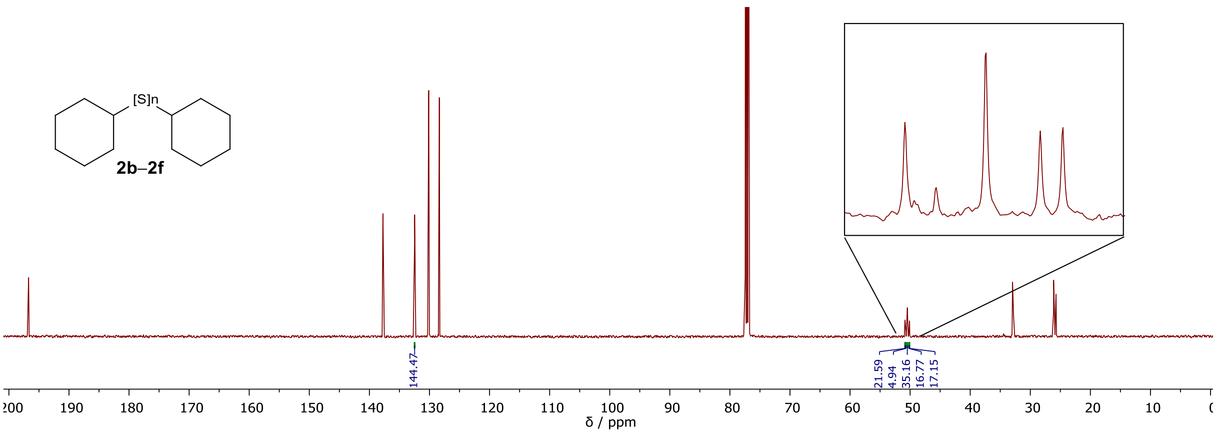

Figure S33: Quantitative ^{13}C NMR spectrum of the dicyclohexyl polysulfides (**2b**–**2f**) from setup **B** (solvent DCM + 10% (vol.) CS_2) at 126 MHz and rt with benzophenone as internal standard. The integral of benzophenone is set the way that the integral of each polysulfide represents its absolute yield.

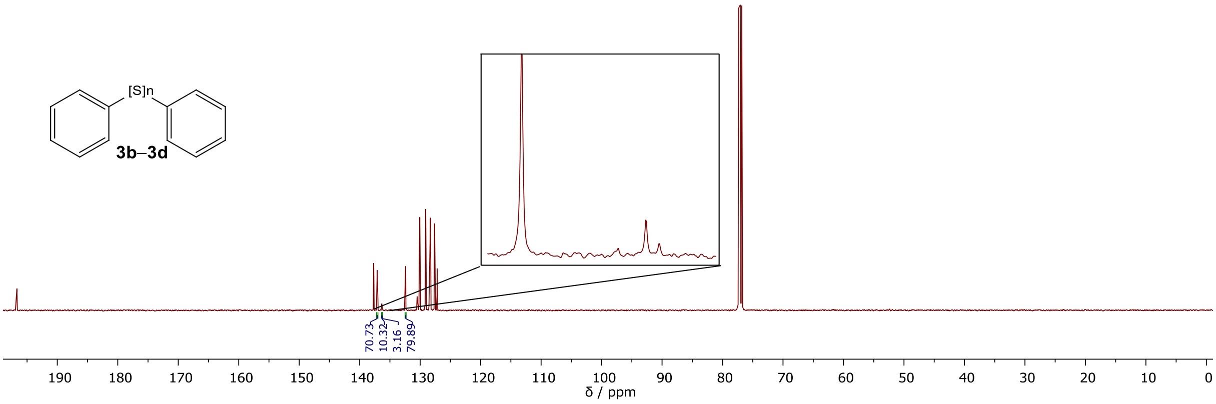

Figure S34: Quantitative ^{13}C NMR spectrum of the diphenyl polysulfides (**3b**–**3d**) from setup **A** (solvent DCM) at 126 MHz and rt with benzophenone as internal standard. The integral of benzophenone is set the way that the integral of each polysulfide represents its absolute yield.

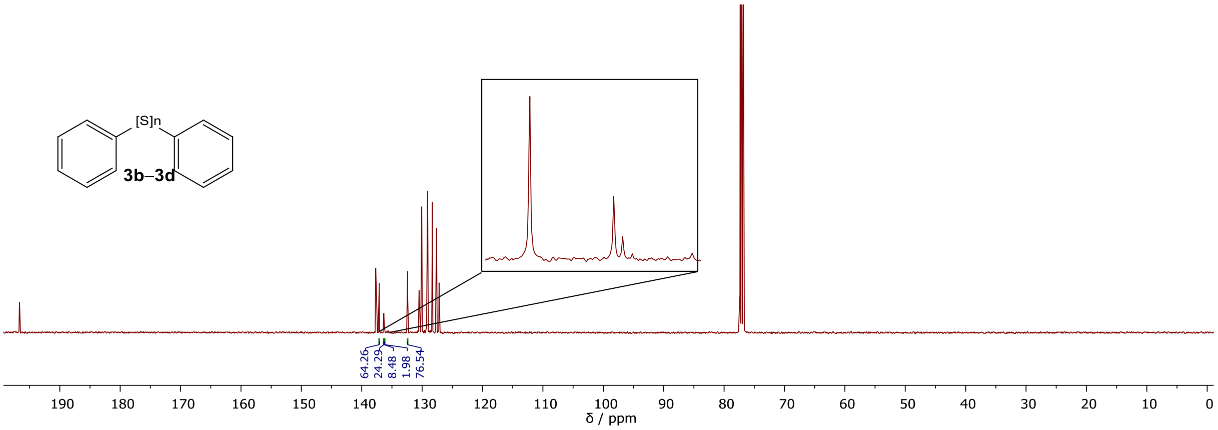

Figure S35: Quantitative ^{13}C NMR spectrum of the diphenyl polysulfides (**3b–3d**) from setup **B** (solvent DCM + 10% (vol.) CS_2) at 126 MHz and rt with benzophenone as internal standard. The integral of benzophenone is set the way that the integral of each polysulfide represents its absolute yield.

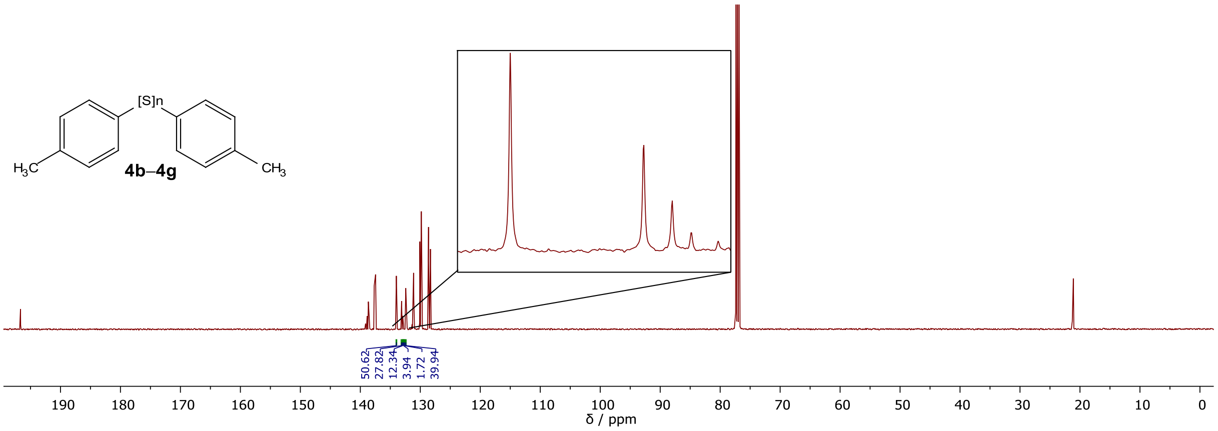

Figure S36: Quantitative ^{13}C NMR spectrum of the di-*p*-tolyl polysulfides (**4b–4g**) from setup **A** (solvent DCM) at 126 MHz and rt with benzophenone as internal standard. The integral of benzophenone is set the way that the integral of each polysulfide represents its absolute yield.

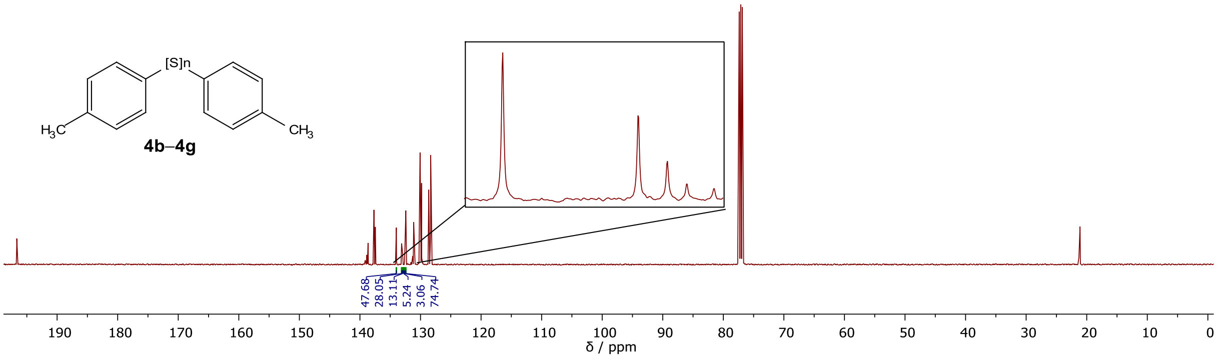

Figure S37: Quantitative ^{13}C NMR spectrum of the di-*p*-tolyl polysulfides (**4b–4g**) from setup **B** (solvent DCM + 10% (vol.) CS_2) at 126 MHz and rt with benzophenone as internal standard. The integral of benzophenone is set the way that the integral of each polysulfide represents its absolute yield.

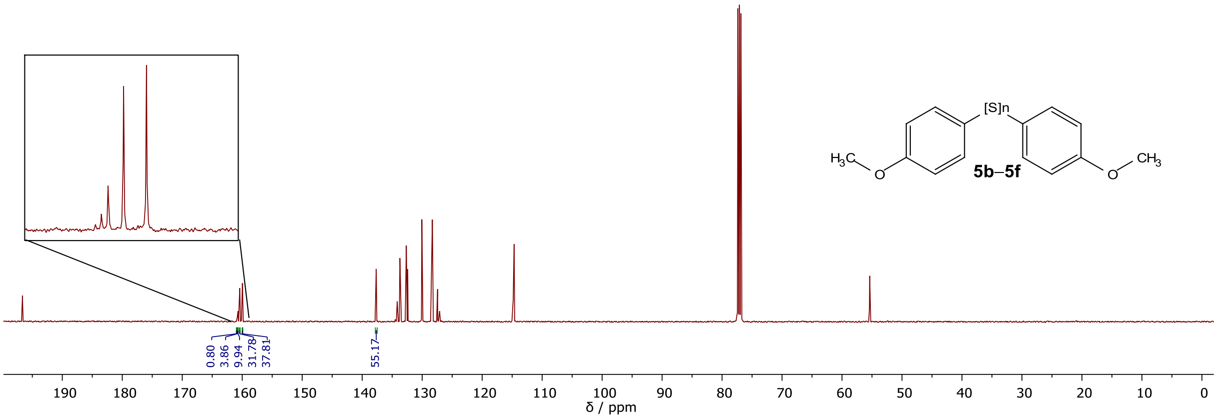

Figure S38: Quantitative ^{13}C NMR spectrum of the di-*p*-methoxyphenyl polysulfides (**5b**–**5f**) from setup **A** (solvent DCM) at 126 MHz and rt with benzophenone as internal standard. The integral of benzophenone is set the way that the integral of each polysulfide represents its absolute yield.

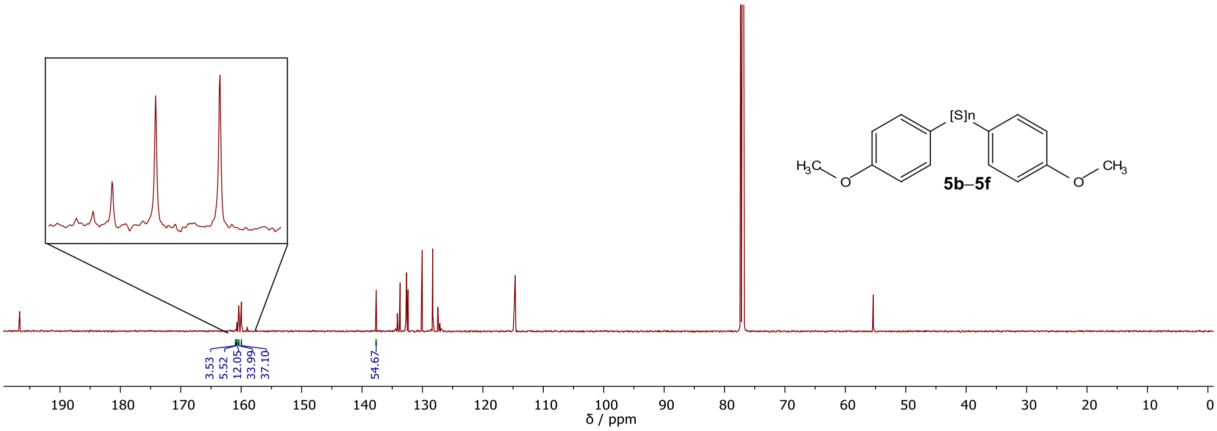

Figure S39: Quantitative ^{13}C NMR spectrum of th edi-*p*-methoxyphenyl polysulfides (**5b**–**5f**) from setup **B** (solvent DCM + 10% (vol.) CS_2) at 126 MHz and rt with benzophenone as internal standard. The integral of benzophenone is set the way that the integral of each polysulfide represents its absolute yield.

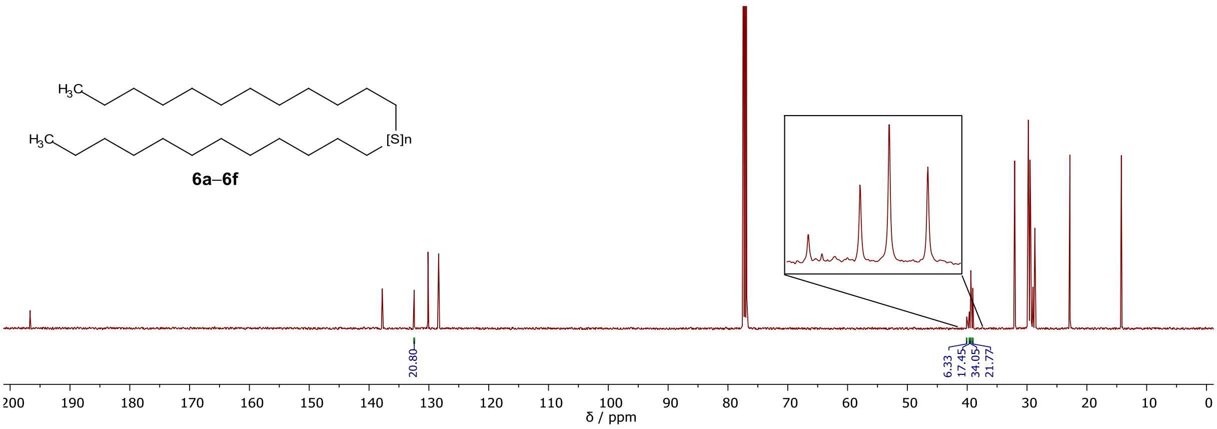

Figure S40: Quantitative ^{13}C NMR spectrum of the di-*n*-dodecyl polysulfides (**6a**–**6f**) from setup **A** (solvent DCM) at 126 MHz and rt with benzophenone as internal standard. The integral of benzophenone is set the way that the integral of each polysulfide represents its absolute yield.

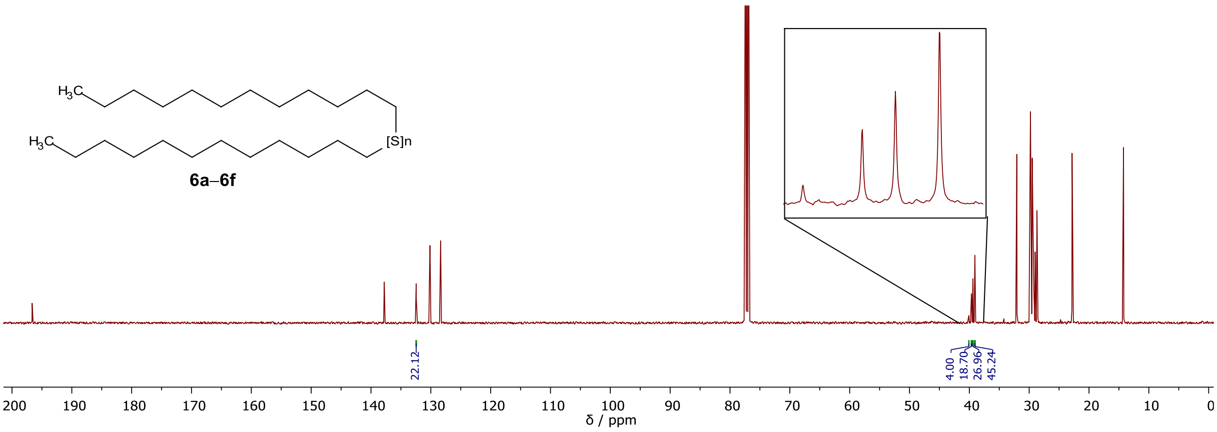

Figure S41: Quantitative ^{13}C NMR spectrum of the di-*n*-dodecyl polysulfides (**6a**–**6f**) from setup **B** (solvent DCM + 10% (vol.) CS_2) at 126 MHz and rt with benzophenone as internal standard. The integral of benzophenone is set the way that the integral of each polysulfide represents its absolute yield.

2.3 HPLC-UV Chromatogramms

2.3.1 Disulfides and starting materials

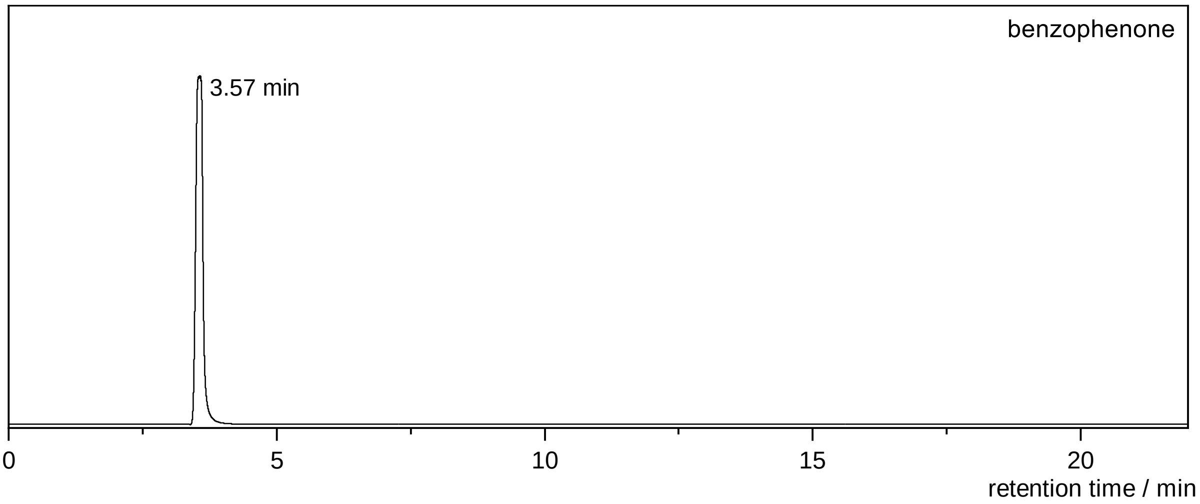

Figure S42: RP-HPLC-UV chromatogram of benzophenone at 248 nm (0.5 mL/min methanol).

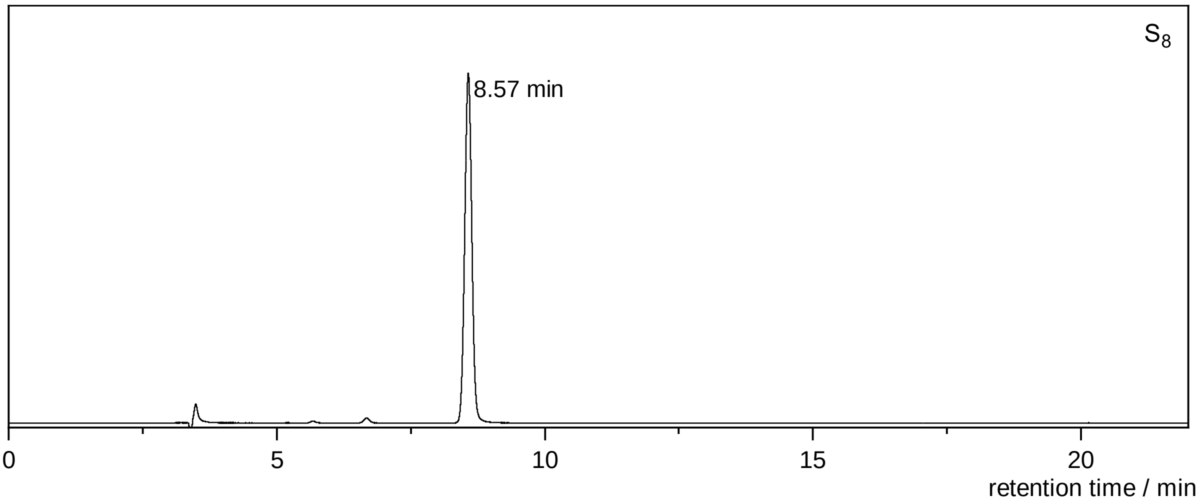

Figure S43: RP-HPLC-UV chromatogram of elemental sulfur at 248 nm (0.5 mL/min methanol).

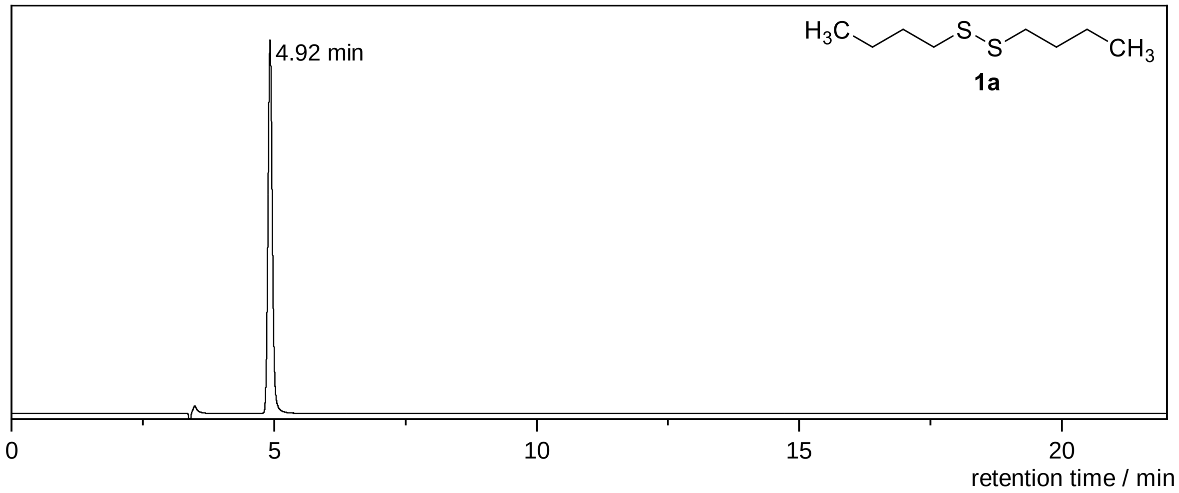

Figure S44: RP-HPLC-UV chromatogram of di-n-butyl disulfide (**1a**) at 248 nm (0.5 mL/min methanol).

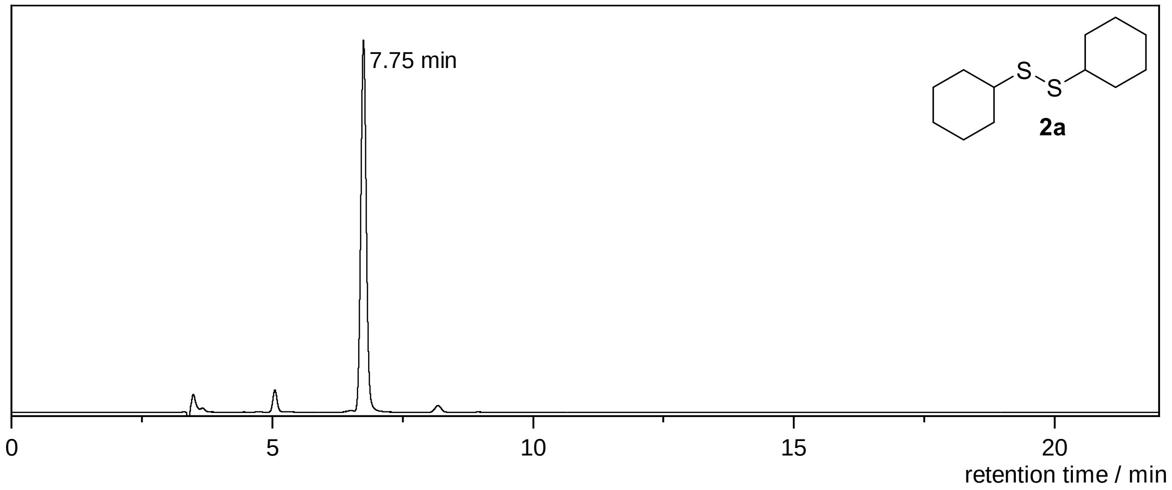

Figure S45: RP-HPLC-UV chromatogram of dicyclohexyl disulfide (**2a**) at 248 nm (0.5 mL/min methanol).

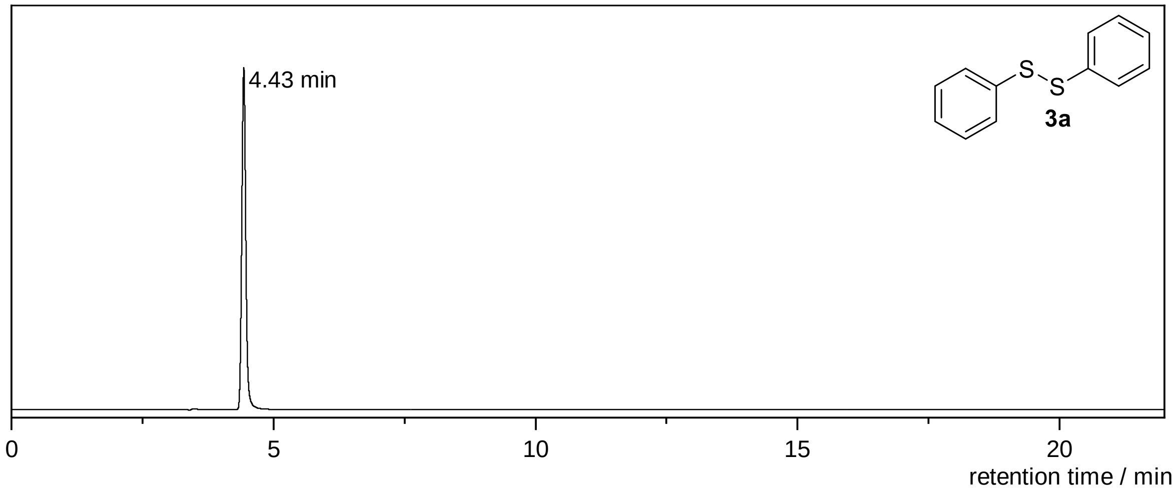

Figure S46: RP-HPLC-UV chromatogram of diphenyl disulfide (**3a**) at 248 nm (0.5 mL/min methanol).

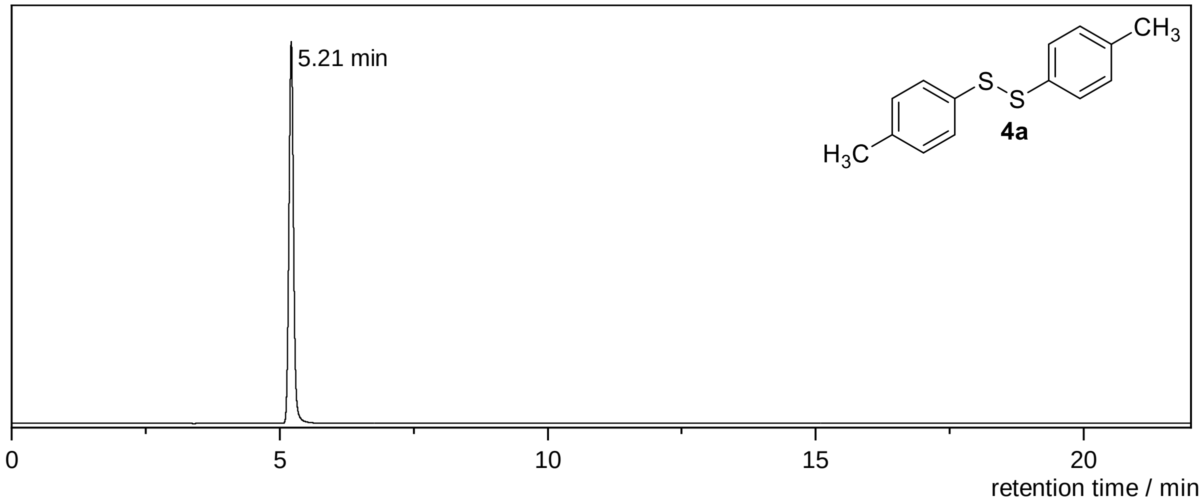

Figure S47: RP-HPLC-UV chromatogram of di-*p*-tolyl disulfide (**4a**) at 248 nm (0.5 mL/min methanol).

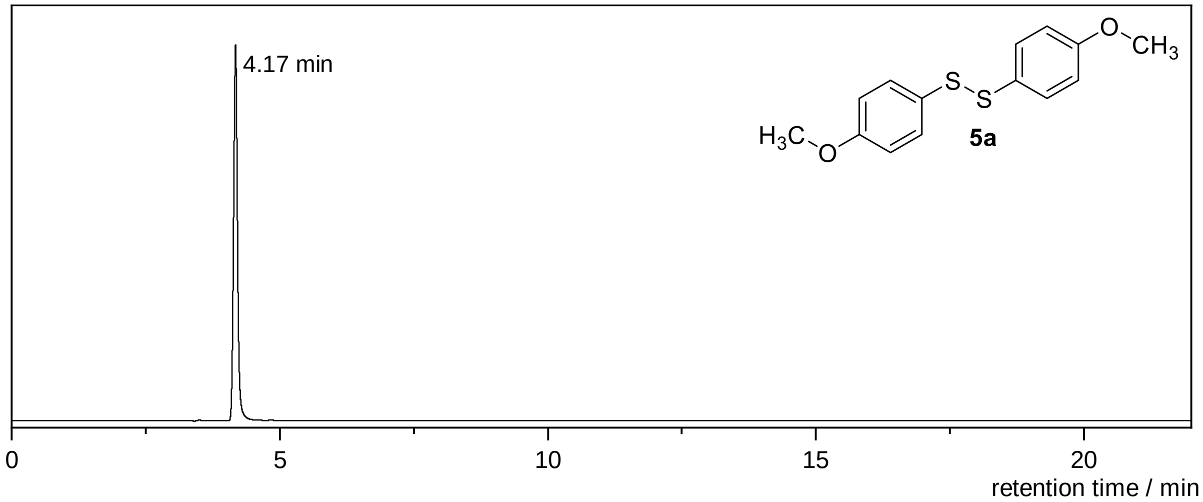

Figure S48: RP-HPLC-UV chromatogram of di-*p*-methoxyphenyl disulfide (**5a**) at 248 nm (0.5 mL/min methanol).

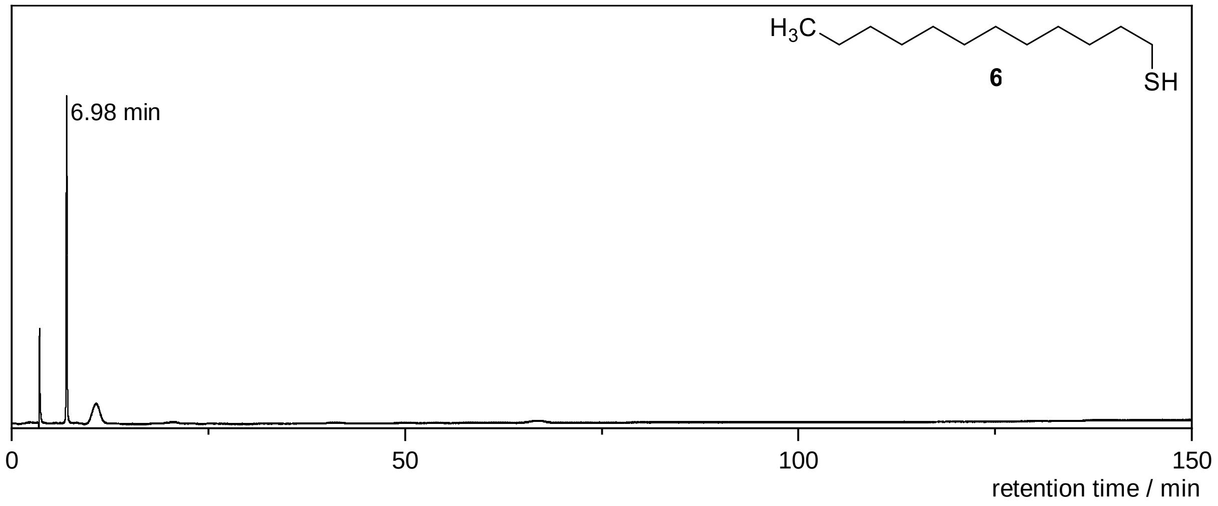

Figure S49: RP-HPLC-UV chromatogram of *n*-dodecylthiol (**6**) at 248 nm (0.5 mL/min methanol).

2.3.2 Polysulfides

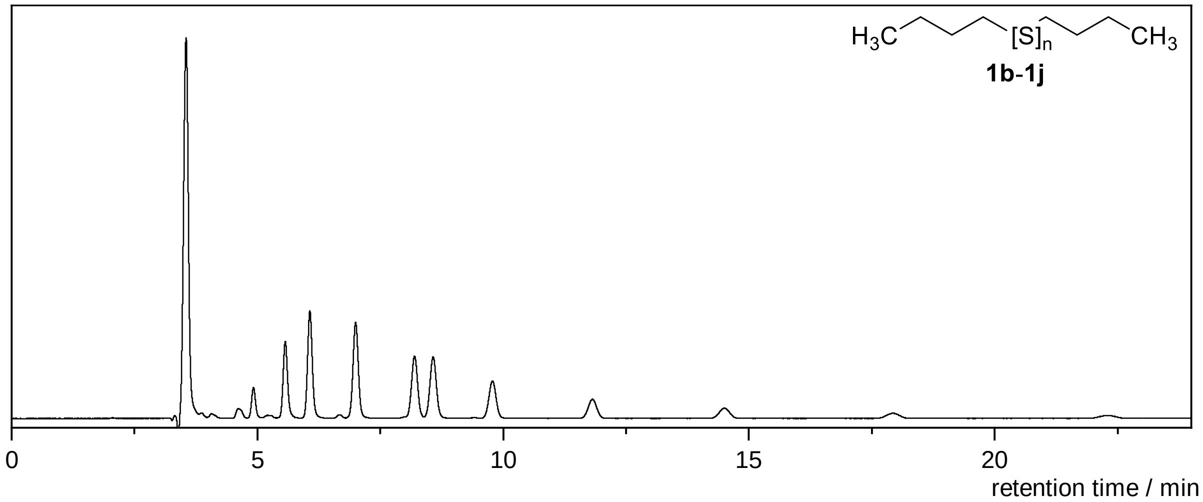

Figure S50: RP-HPLC-UV chromatogram of the di-*n*-butyl polysulfides (**1b**–**1j**) from reaction setup **A** (solvent DCM) at 248 nm (0.5 mL/min methanol).

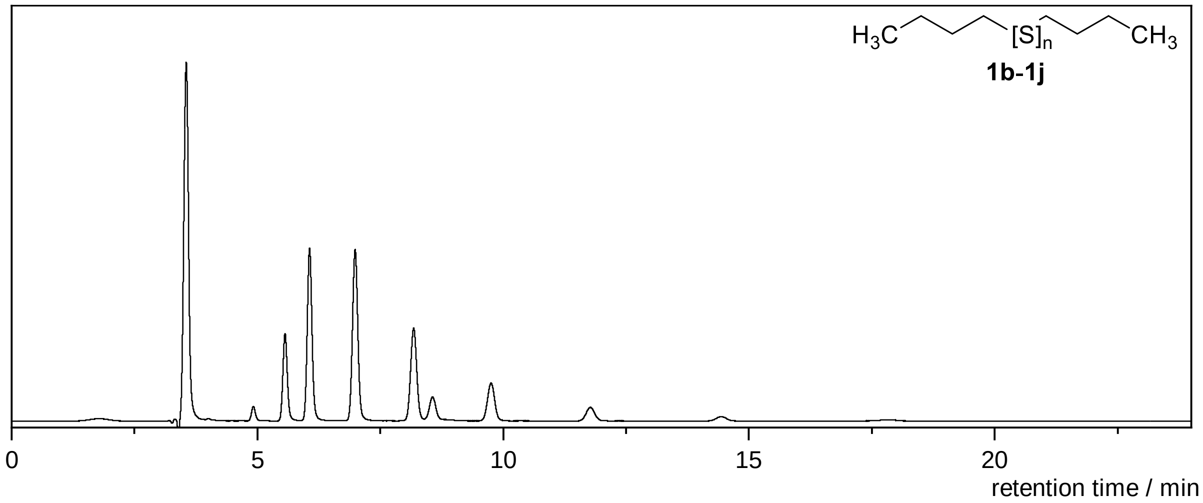

Figure S51: RP-HPLC-UV chromatogram of the di-*n*-butyl polysulfides (**1b**–**1j**) from reaction setup **B** (solvent DCM + 10% (vol.) CS_2) at 248 nm (0.5 mL/min methanol).

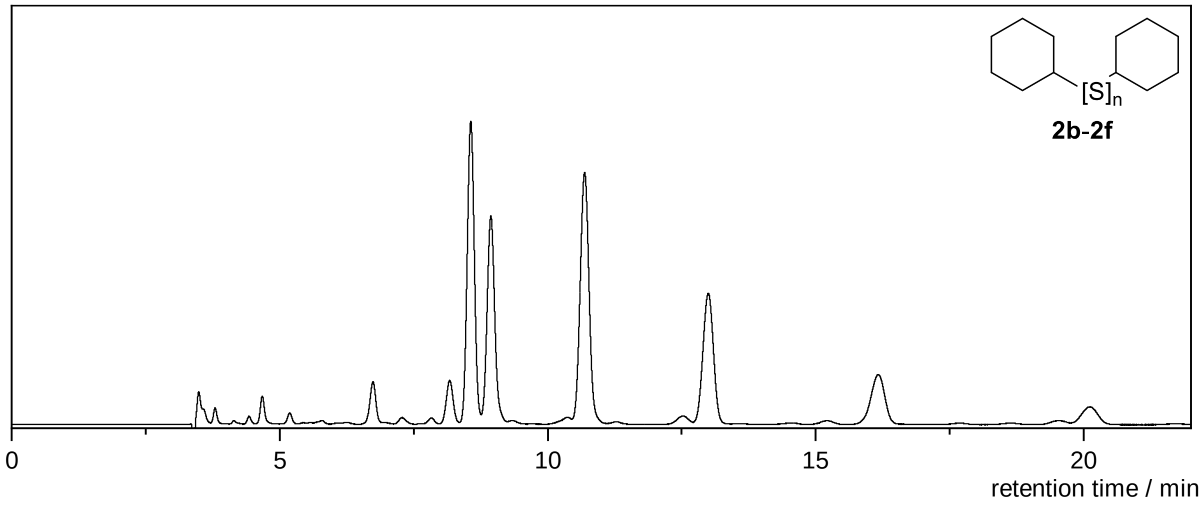

Figure S52: RP-HPLC-UV chromatogram of the dicyclohexyl polysulfides (**2b**–**2f**) from reaction setup **A** (solvent DCM) at 248 nm (0.5 mL/min methanol).

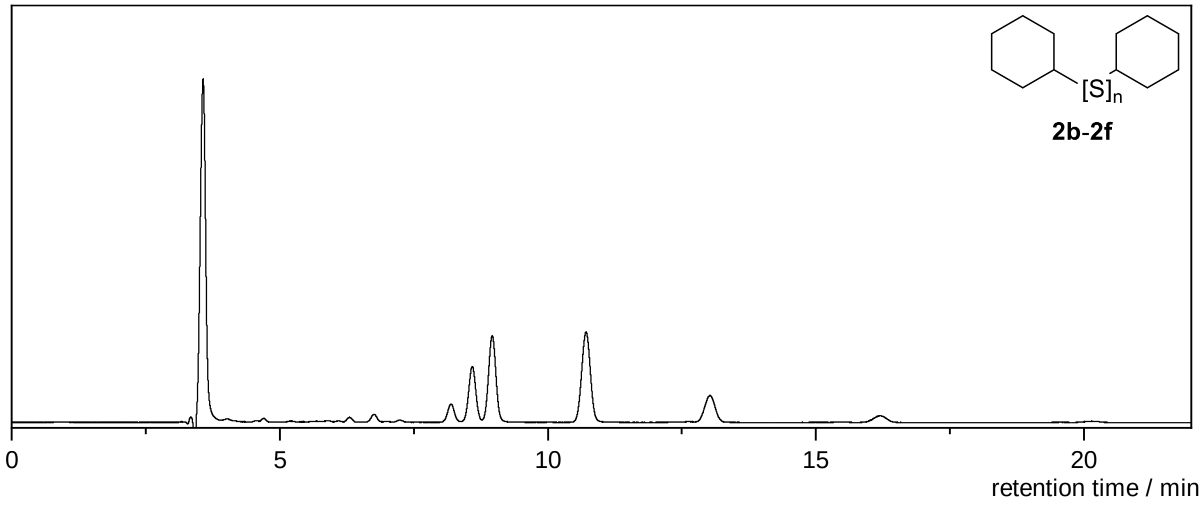

Figure S53: RP-HPLC-UV chromatogram of the dicyclohexyl polysulfides (**2b**–**2f**) from reaction setup **B** (solvent DCM + 10% (vol.) CS_2) at 248 nm (0.5 mL/min methanol).

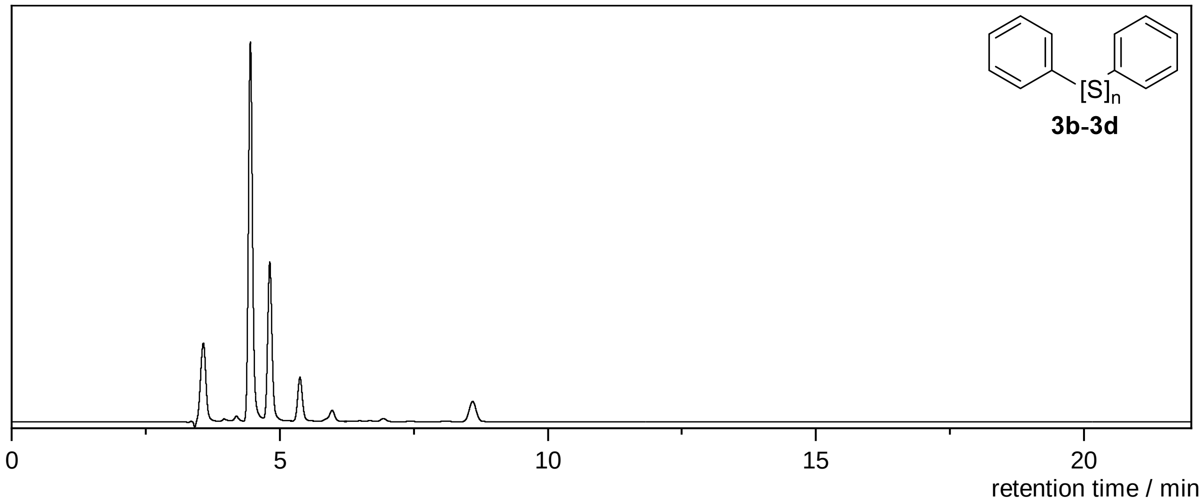

Figure S54: RP-HPLC-UV chromatogram of the diphenyl polysulfides (**3b**–**3d**) from reaction setup **A** (solvent DCM) at 248 nm (0.5 mL/min methanol).

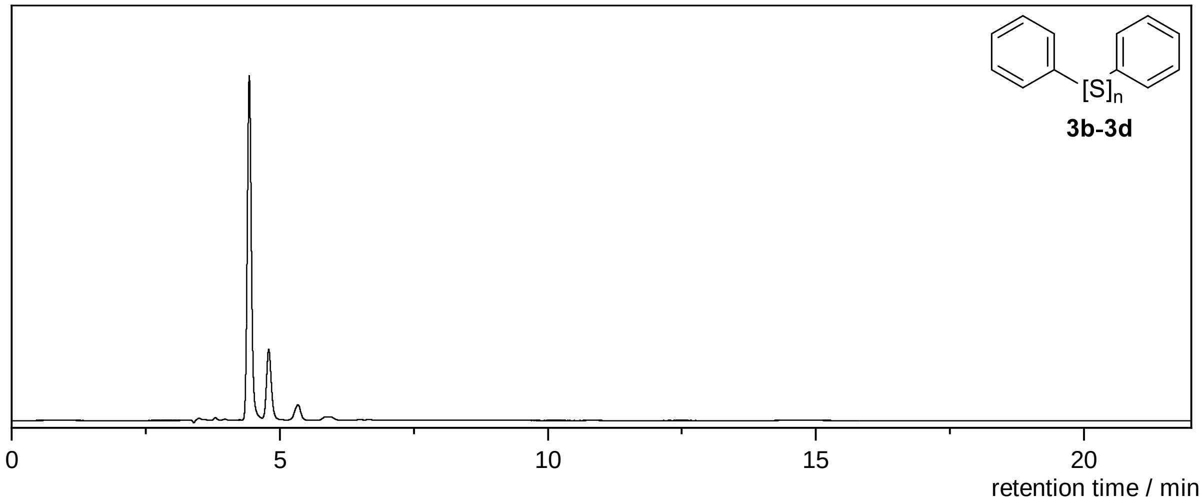

Figure S55: RP-HPLC-UV chromatogram of the diphenyl polysulfides (**3b**–**3d**) from reaction setup **B** (solvent DCM + 10% (vol.) CS_2) at 248 nm (0.5 mL/min methanol).

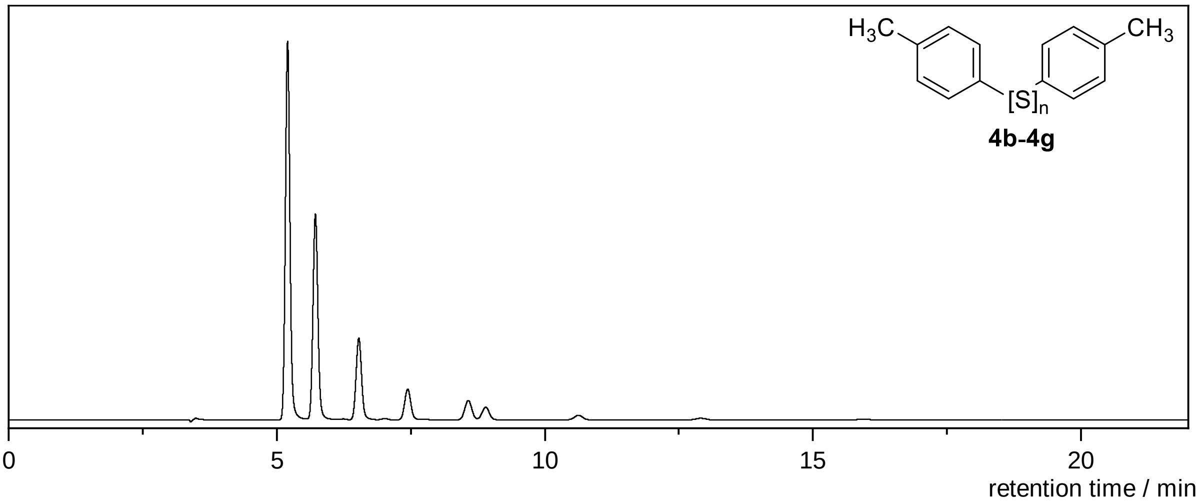

Figure S56: RP-HPLC-UV chromatogram of the di-*p*-tolyl polysulfides (**4b–4g**) from reaction setup **A** (solvent DCM) at 248 nm (0.5 mL/min methanol).

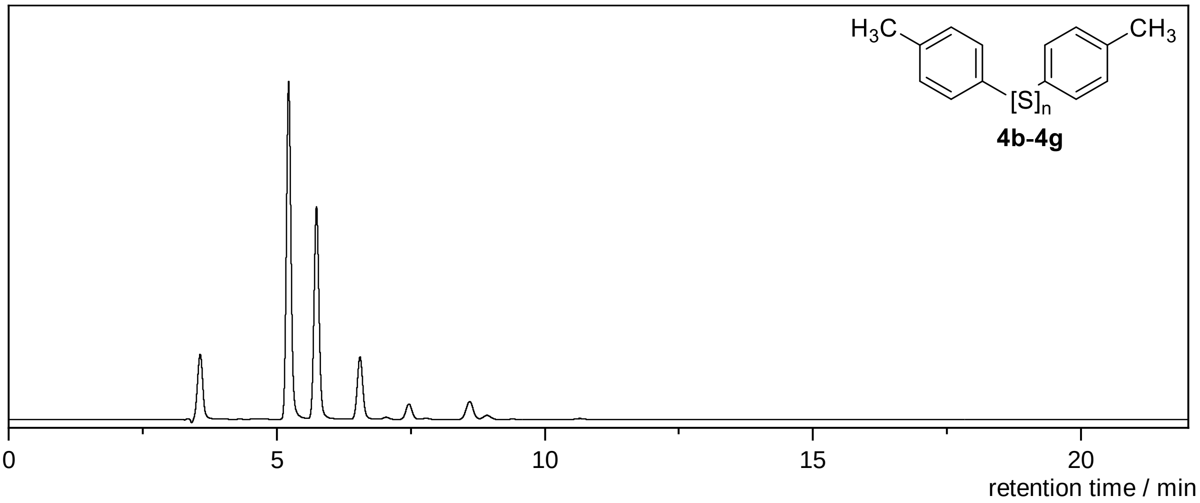

Figure S57: RP-HPLC-UV chromatogram of the di-*p*-tolyl polysulfides (**4b–4g**) from reaction setup **B** (solvent DCM + 10% (vol.) CS_2) at 248 nm (0.5 mL/min methanol).

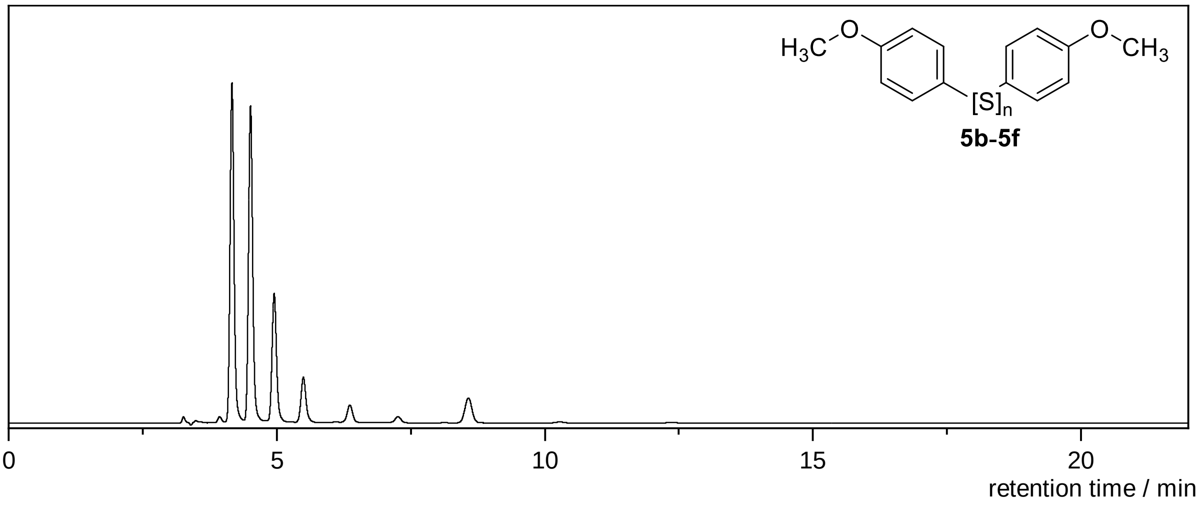

Figure S58: RP-HPLC-UV chromatogram of the di-*p*-methoxyphenyl polysulfides (**5b**–**5f**) from reaction setup **A** (solvent DCM) at 248 nm (0.5 mL/min methanol).

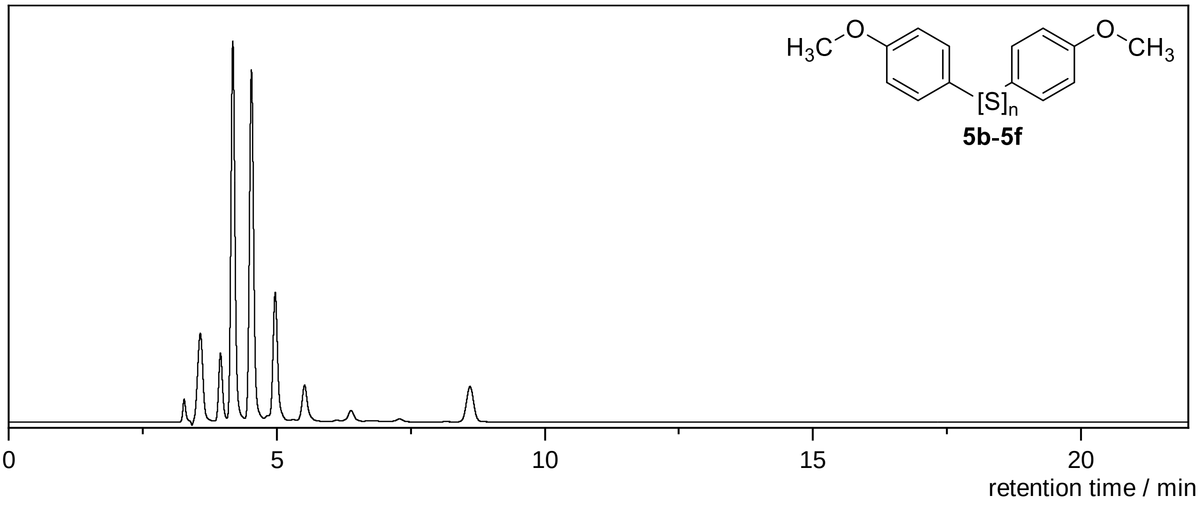

Figure S59: RP-HPLC-UV chromatogram of the di-*p*-methoxyphenyl polysulfides (**5b**–**5f**) from reaction setup **B** (solvent DCM + 10% (vol.) CS_2) at 248 nm (0.5 mL/min methanol).

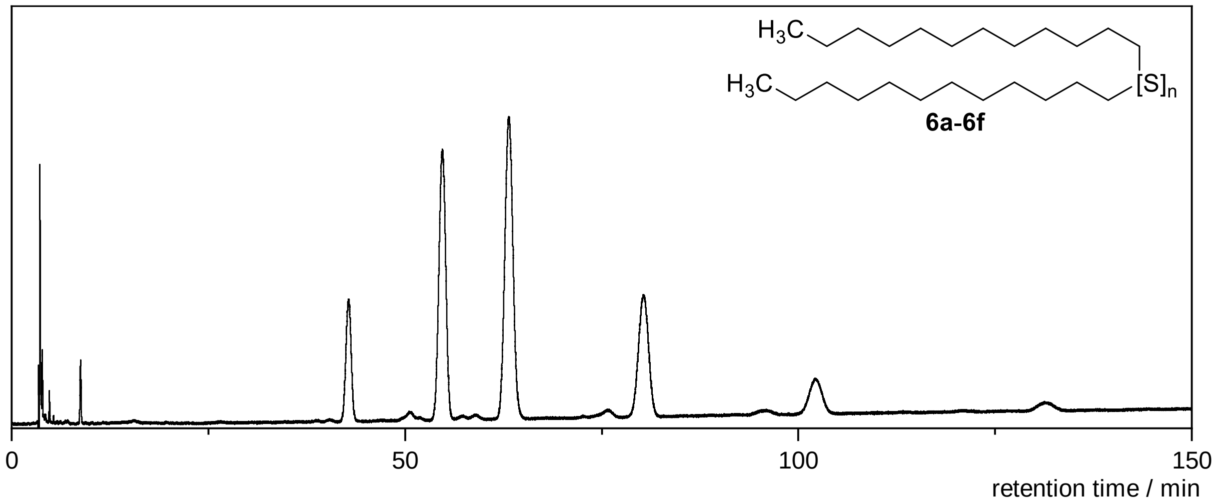

Figure S60: RP-HPLC-UV chromatogram of the di-*n*-dodecyl polysulfides (**6a**–**6f**) from reaction setup **A** (solvent DCM) at 248 nm (0.5 mL/min methanol).

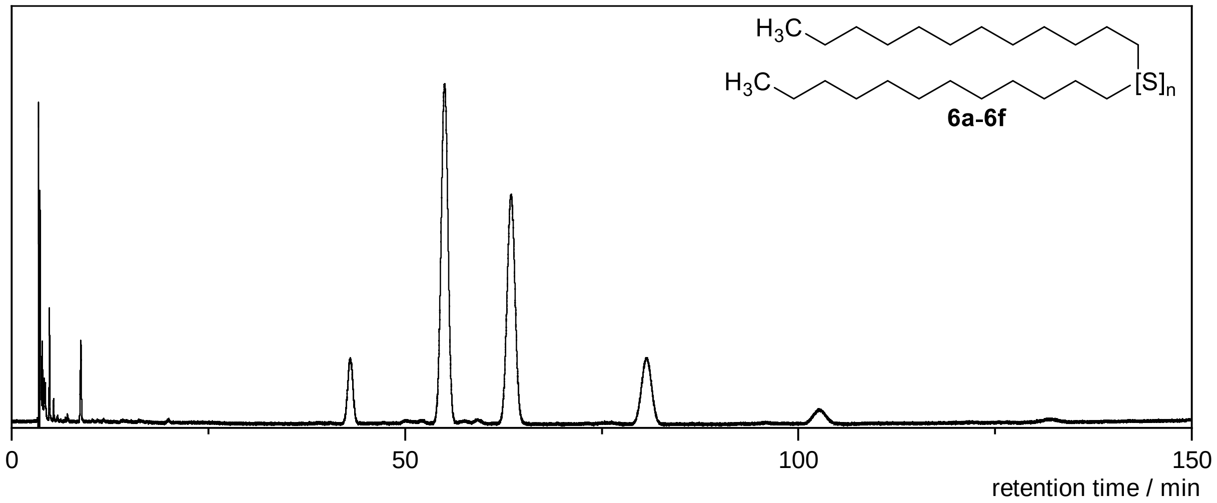

Figure S61: RP-HPLC-UV chromatogram of the di-*n*-dodecyl polysulfides (**6a**–**6f**) from reaction setup **B** (solvent DCM + 10% (vol.) CS_2) at 248 nm (0.5 mL/min methanol).

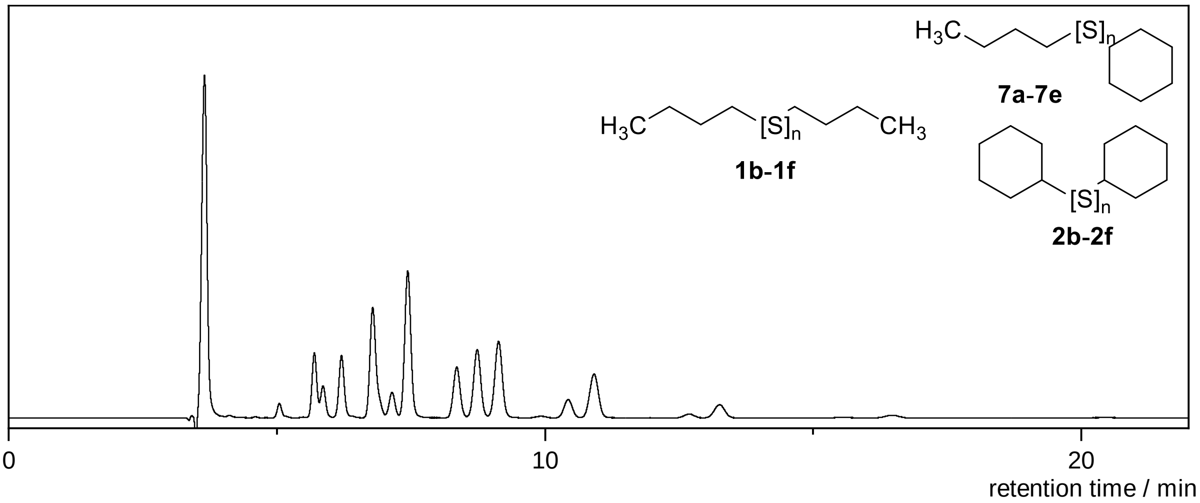

Figure S62: RP-HPLC-UV chromatogram of the di-*n*-butyl polysulfides (**1a**–**1f**), the dicyclohexyl polysulfides (**2b**–**2f**) and the *n*-butylcyclohexyl polysulfides (**7a**–**7e**) from reaction setup **B** (solvent DCM + 10% (vol.) CS_2) at 248 nm (0.5 mL/min methanol).

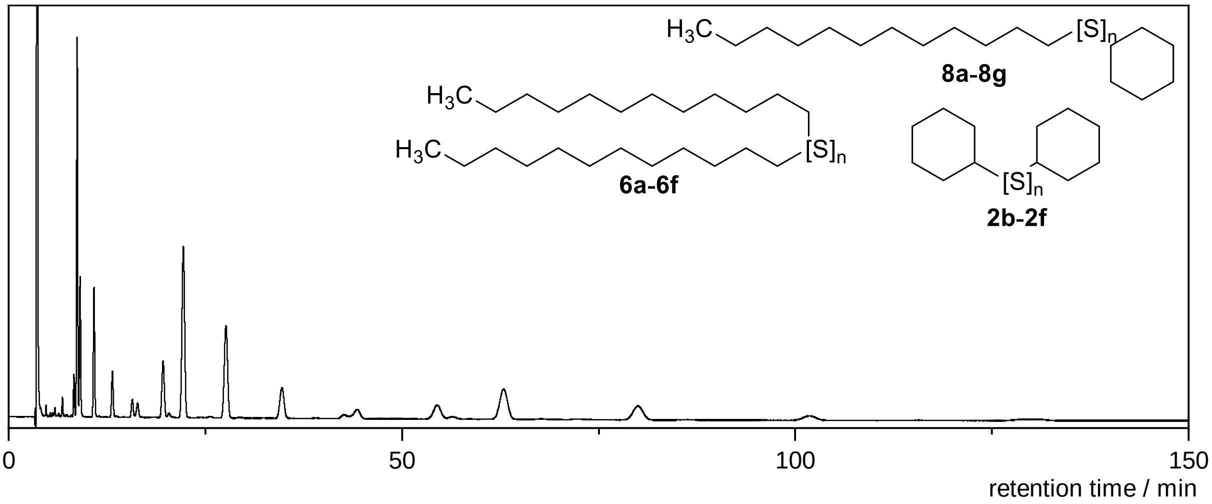

Figure S63: RP-HPLC-UV chromatogram of the di-*n*-dodecyl polysulfides (**6a**–**6f**), the dicyclohexyl polysulfides (**2b**–**2f**) and the cyclohexyl-*n*-dodecyl polysulfides (**8a**–**8f**) from reaction setup **B** (solvent DCM + 10% (vol.) CS_2) at 248 nm (0.5 mL/min methanol).

2.4 UV-Vis spectra

2.4.1 Disulfides and starting material

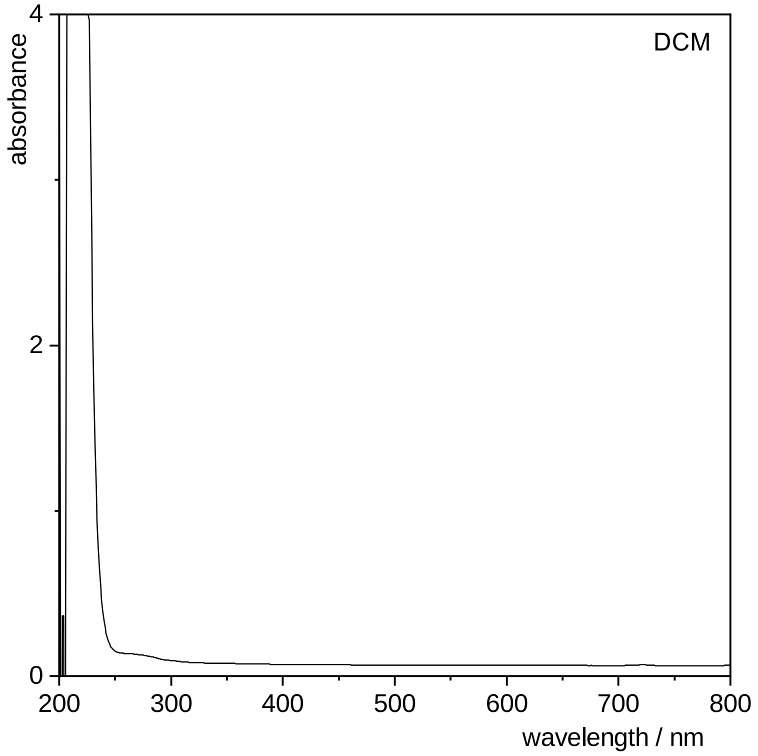

Figure S64: UV-Vis spectrum of DCM at rt.

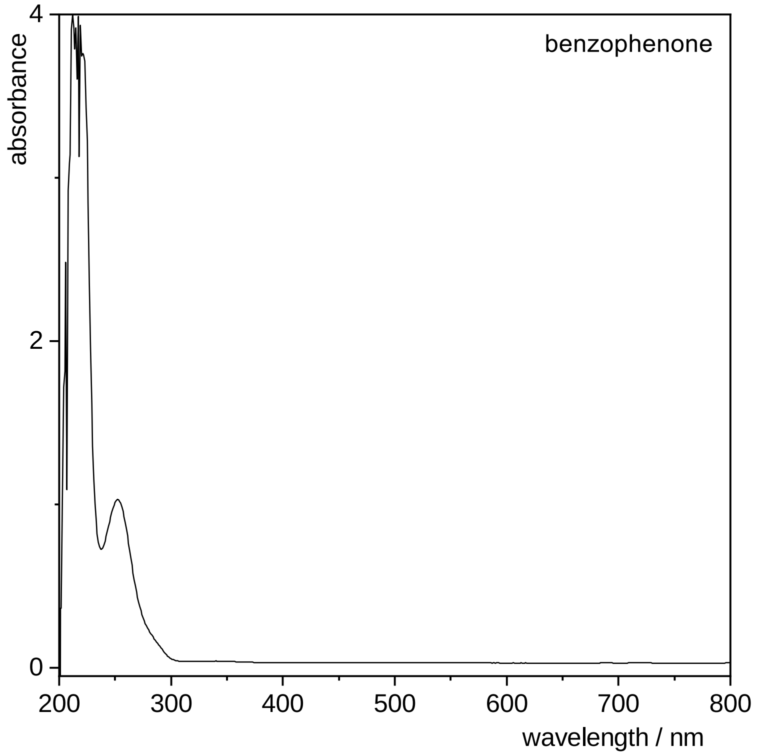

Figure S65: UV-Vis spectrum of benzophenone in DCM at rt.

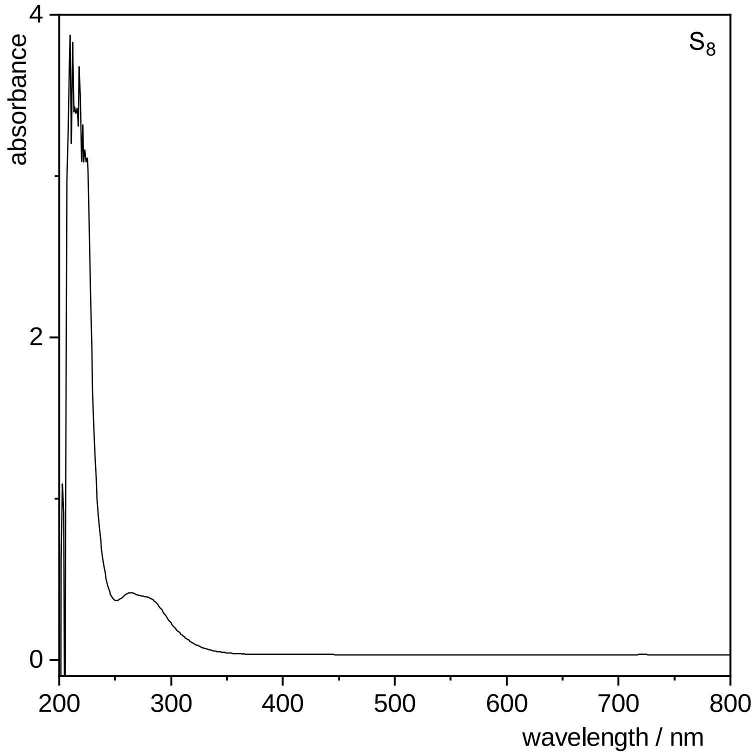

Figure S66: UV-Vis spectrum of elemental sulfur in DCM at rt.

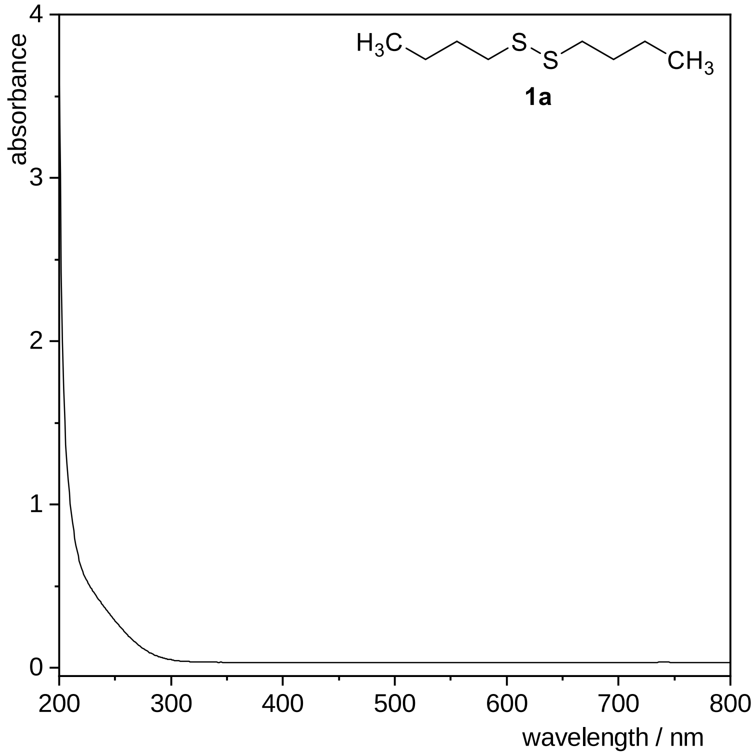

Figure S67: UV-Vis spectrum of di-*n*-butyl disulfide (**1a**) in DCM at rt.

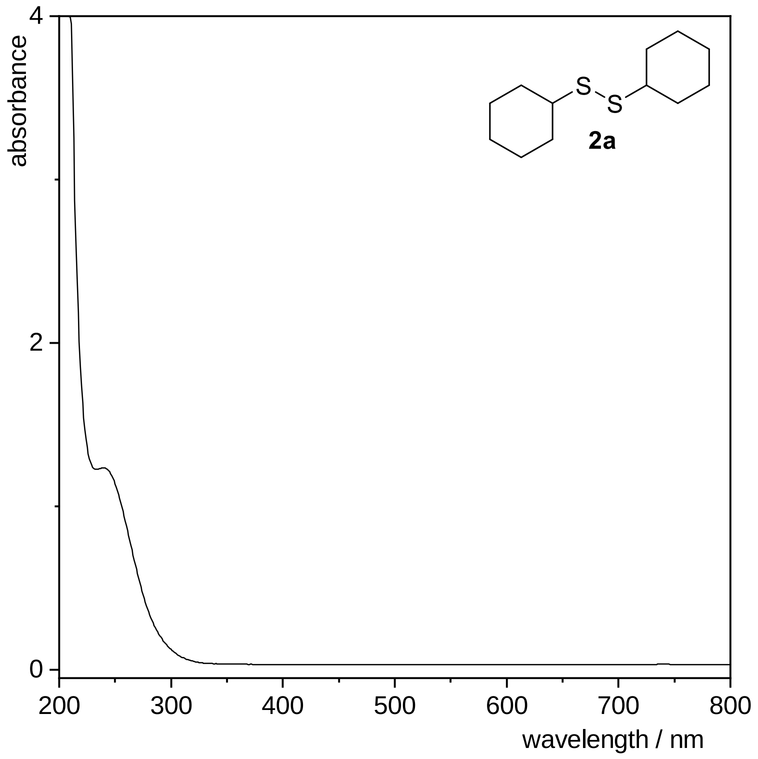

Figure S68: UV-Vis spectrum of dicyclohexyl disulfide (**2a**) in DCM at rt.

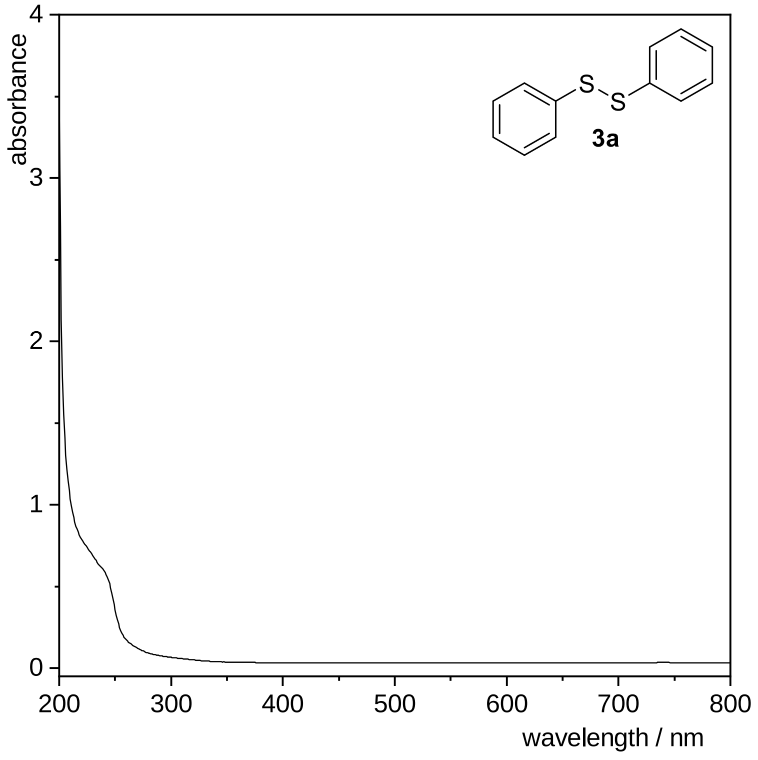

Figure S69: UV-Vis spectrum of diphenyl disulfide (**3a**) in DCM at rt.

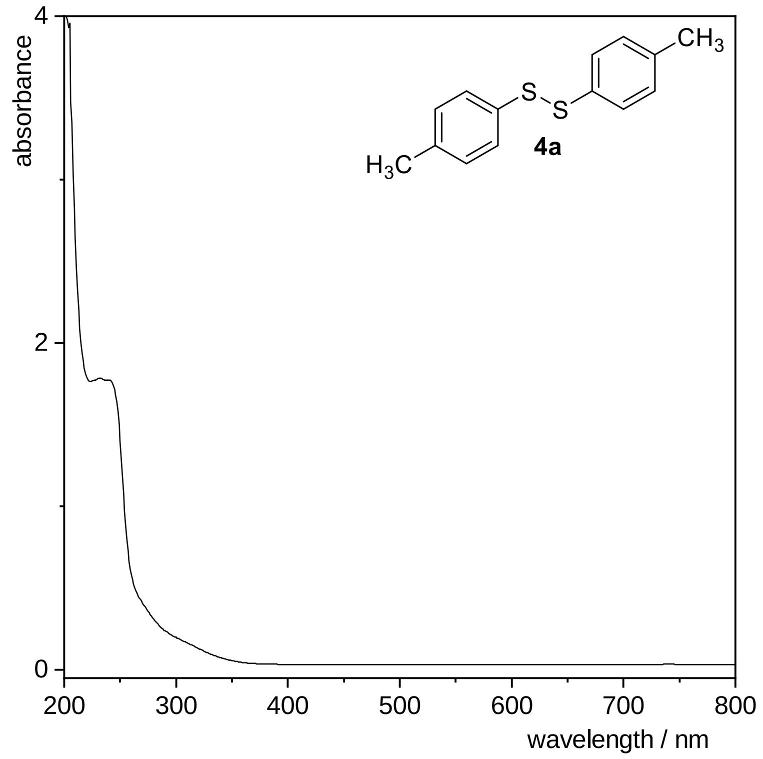

Figure S70: UV-Vis spectrum of di-*p*-tolyl disulfide (**4a**) in DCM at rt.

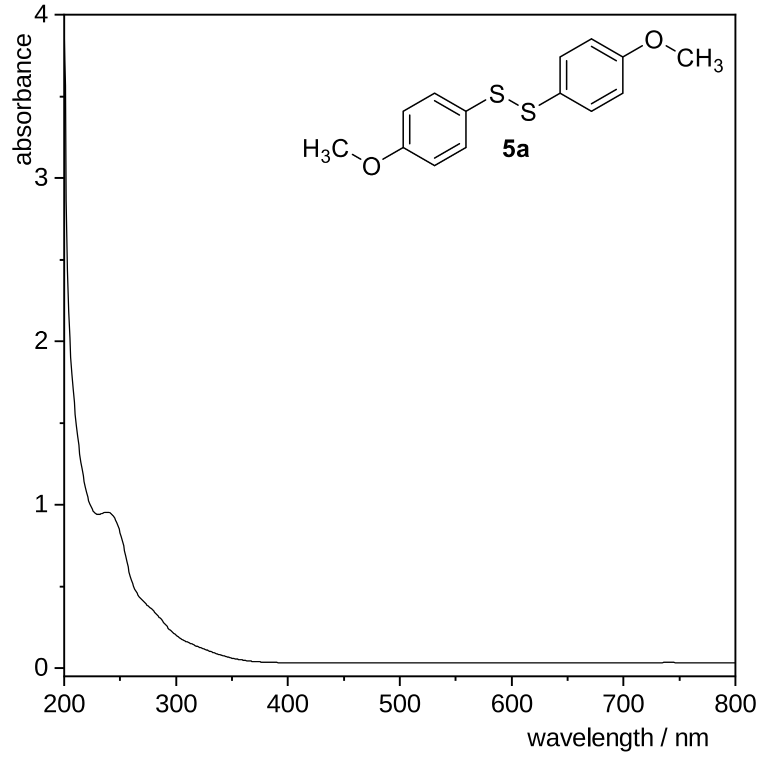

Figure S71: UV-Vis spectrum of di-*p*-methoxyphenyl disulfide (**5a**) in DCM at rt.

2.5 GC-MS chromatogramms of mixed polysulfides

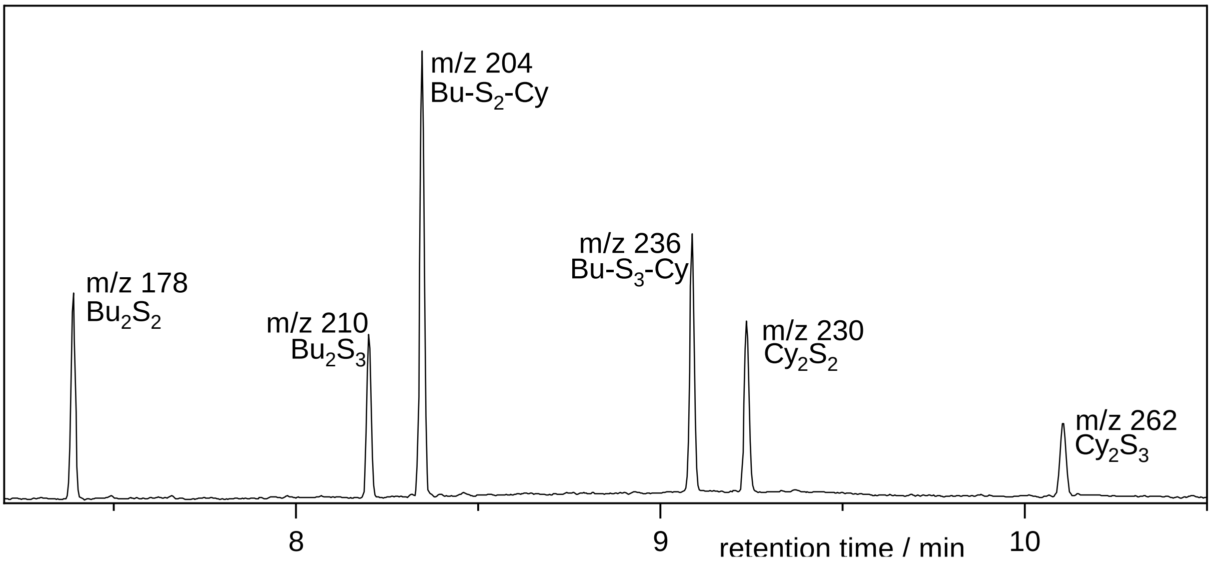

Figure S72: GC-MS chromatogram of the di-*n*-butyl polysulfides (**1a**–**1f**), the dicyclohexyl polysulfides (**2b**–**2f**) and the *n*-butylcyclohexyl polysulfides (**7a**–**7e**) from reaction setup **B** (solvent DCM + 10% (vol.) CS_2) indicating the formed disulfides and trisulfides. Higher sulfides could not be detected by GC-MS. Bu = *n*-butyl, Cy = cyclohexyl.

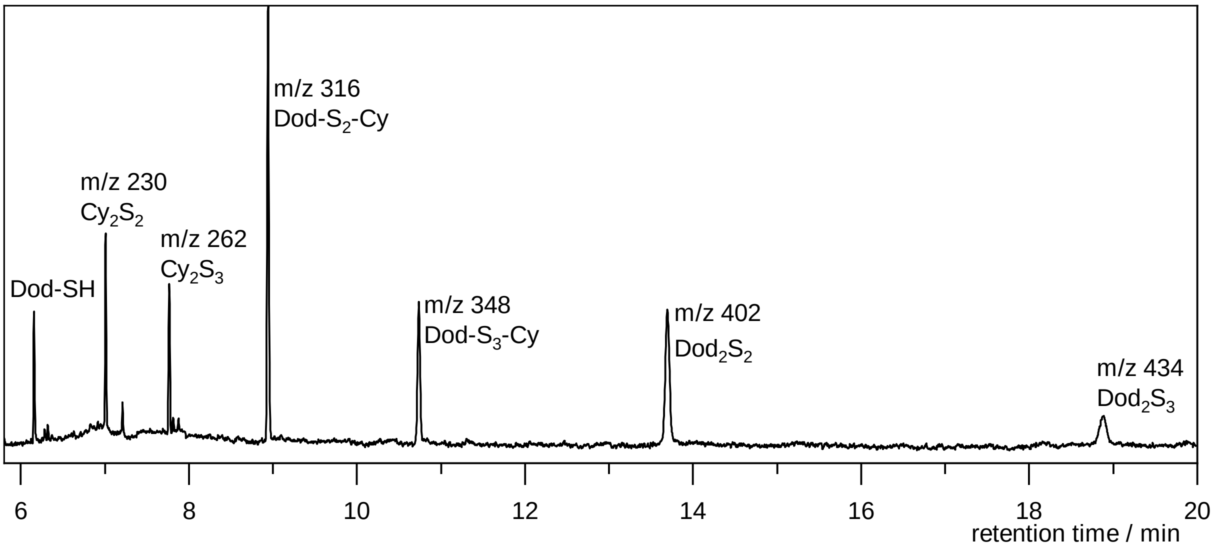

Figure S73: GC-MS chromatogram of the di-*n*-dodecyl polysulfides (**6a**–**6f**), the dicyclohexyl polysulfides (**2b**–**2f**) and the cyclohexyl-*n*-dodecyl polysulfides (**8a**–**8f**) from reaction setup **B** (solvent DCM + 10% (vol.) CS_2) indicating the formed disulfides and trisulfides. Higher sulfides could not be detected by GC-MS. Ded = *n*-dodecyl, Cy = cyclohexyl.

2.6 Cyclic Voltammetry

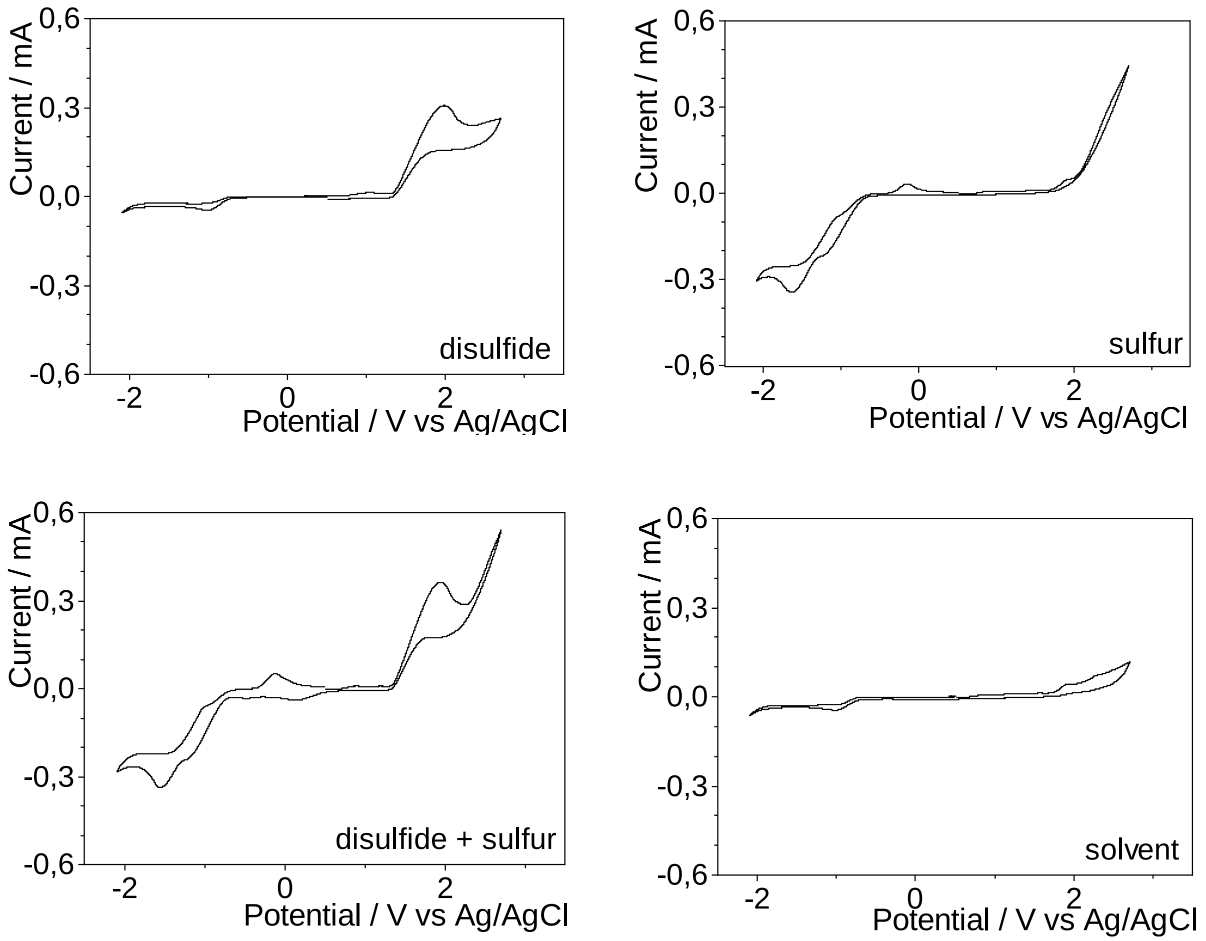

Figure S74: Cyclic voltammogram of (top left) 0.13 mmol di-*n*-butyl disulfide (**1a**), (top right) 0.031 mmol S_8 (= 2 eq. [S]), (bottom left) 0.13 mmol di-*n*-butyl disulfide (**1a**) and 0.031 mmol S_8 and (bottom right) the pure solvent with the supporting electrolyte. All measurements were performed in 5 mL DCM with 0.5 mmol $n$$Bu_4NBF_4$ as supporting electrolyte at a glassy carbon disk electrode (2.0 mm diameter at a scan rate of 100 mV·sec^{-1}.

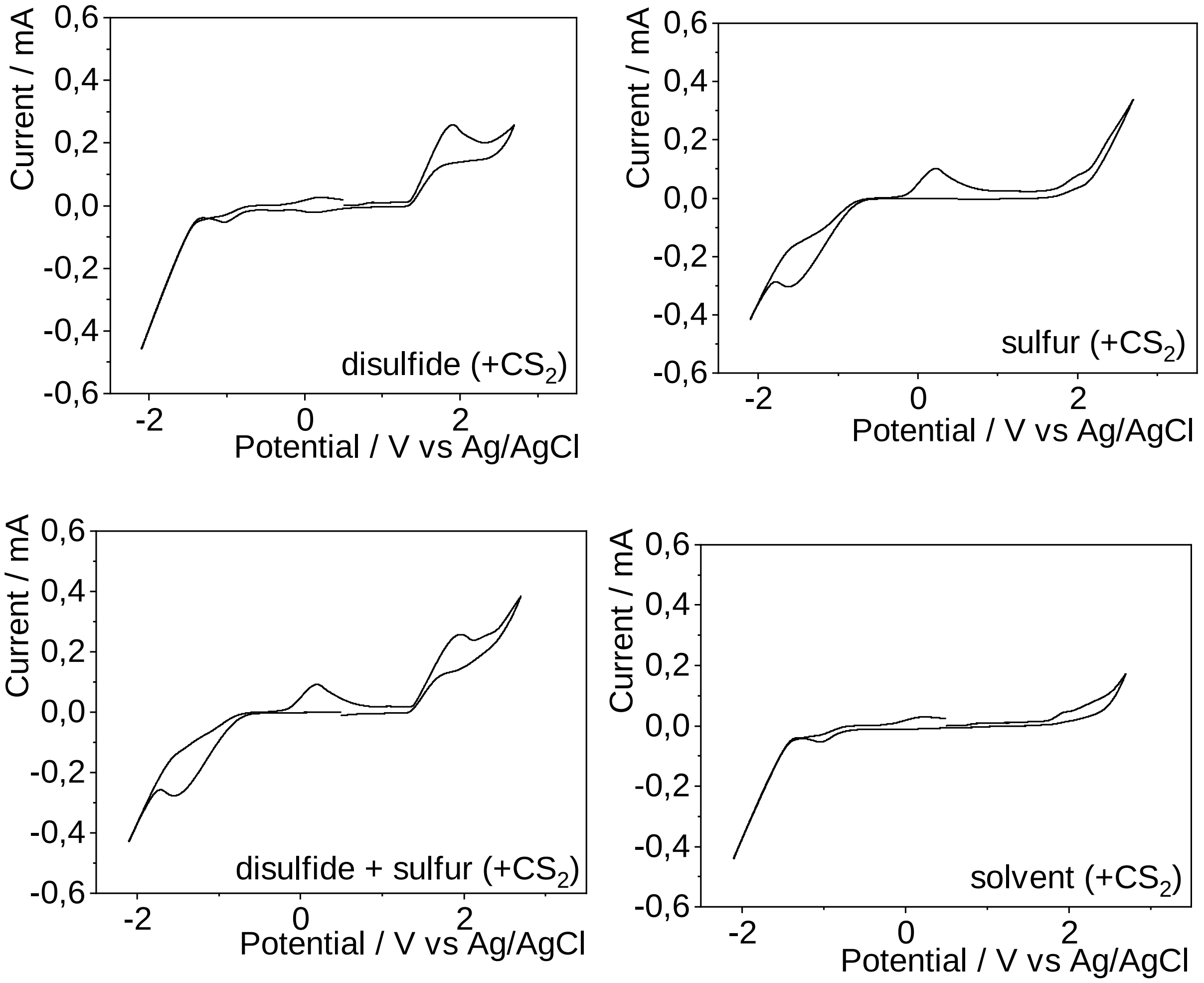

Figure S75: Cyclic voltammogram of (top left) 0.13 mmol di-*n*-butyl disulfide (**1a**), (top right) 0.031 mmol S_8 (= 2 eq. [S]), (bottom left) 0.13 mmol di-*n*-butyl disulfide (**1a**) and 0.031 mmol S_8 and (bottom right) the pure solvent with the supporting electrolyte. All measurements were performed in 4 mL DCM + 1 mL CS_2 with 0.5 mmol nBu$_4$NBF$_4$ as supporting electrolyte at a glassy carbon disk electrode (2.0 mm diameter at a scan rate of 100 mV·sec^{-1}.

3 Calculation of sulfur amount in polysulfides (SAP_{248}) and dispersity ($Đ$)

3.1 Definition of SAP248 and dispersity Đ

According to the number average molar mass in polymer chemistry, we define the "*absorbance average* **s**ulfur **a**mount in **p**olysulfides at 248 nm" (SAP_{248}) as

$$\mathrm{SAP}_{248} = \frac{\sum_{i=1}^{\infty} n_{i,248} \cdot N_i(\mathrm{S})}{\sum_{i=1}^{\infty} n_{i,248}} \quad (1)$$

$n_{i,248}$: referenced HPLC-integral of polysulfide i at 248 nm

$N_i(S)$: number of sulfur equivalents in polysulfide i

The SAP_{248} is based on the specific absorption-wavelength of organic polysulfides (248 nm). The SAP_{248} gives the average amount of sulfur atoms in polysulfides calculated on referenced HPLC-UV-spectra. The SAP therefore depends on the absorption coefficients and their ratios of a polysulfide. The SAP is suitable for comparison of a single polysulfide, not for comparison of different polysulfides nor for giving the absolute average sulfur amount.

Based on the mass average molar mass and following from (1) applies

$$(\mathrm{w})\mathrm{SAP}_{248} = \frac{\sum_{i=1}^{\infty} n_{i,248} \cdot N_i^2(\mathrm{S})}{\sum_{i=1}^{\infty} n_{i,248} \cdot N_i(\mathrm{S})} \quad (2)$$

$n_{i,248}$: referenced HPLC-integral of polysulfide i at 248 nm

$N_i(S)$: number of sulfur equivalents in polysulfide i

With (1) and (2) the dispersity is defined as

$$Đ = \frac{(2)}{(1)} = \frac{(\mathrm{w})\mathrm{SAP}_{248}}{\mathrm{SAP}_{248}} \quad (3)$$

3.2 Original Data for SAP and *Đ*-calculation

Table S 1: Integrals of di-*n*-butyl polysulfide (**1a**–**1j**) in HPLC-UV at 248 nm and their calculated SAP and *Đ*.

Integral of	2eq [S] 50 %vol. CS_2	2eq [S] 25 %vol. CS_2	2eq [S] 2 %vol. CS_2	8eq [S] 10 %vol. CS_2	4eq [S] 10 %vol. CS_2	2eq [S] 10 %vol. CS_2	1eq [S] 10 %vol. CS_2	0eq [S] 10 %vol. CS_2	8eq [S] DCM only	4eq [S] DCM only	3eq [S] DCM only	2eq [S] DCM only	1eq [S] DCM only	0.5eq [S] DCM only	N (S)
	0,00219	0,00249	0,00466	0,00476	0,00288	0,0028	0,00402	0,00428	0,00752	0,00775	0,0072	0,00787	0,01224	0,01461	2
	0,02014	0,02118	0,02778	0,02343	0,02374	0,02622	0,02719	0,02694	0,01738	0,02357	0,02273	0,02461	0,02132	0,00707	3
	0,05585	0,06754	0,06701	0,05736	0,07295	0,07404	0,06057	0,05107	0,0254	0,03271	0,03402	0,03215	0,01873	0,00686	4
	0,07508	0,05879	0,04742	0,03882	0,04333	0,0425	0,05008	0,03441	0,033	0,03307	0,03372	0,03244	0,01096	0,00291	5
	0,05004	0,03354	0,01698	0,01787	0,01772	0,01703	0,02029	0,01514	0,0363	0,02566	0,02587	0,02402	0,00548	0,00162	6
	0,02302	0,01487	0,00561	0,00735	0,00642	0,00672	0,00515	0,00491	0,027	0,01876	0,01902	0,01689	0,00241	7,77E-04	7
	0,00854	0,0052	0,00164	0,00268	0,00193	0,00186	0,00203	0,00277	0,01787	0,01287	0,012	0,01052	8,42E-04	4,29E-04	8
	0,003	0,0017	3,79E-04	8,43E-04	3,97E-04	3,97E-04	0	0,00154	0,01171	0,00861	0,00736	0,00705	1,85E-04	1,84E-04	9
	9,26E-04	4,63E-04	0	1,39E-04	0	0	0	0,0013	0,0076	0,00523	0,00433	0,00446	0	6,08E-05	10
	0	0	0	0	0	0	0	0,00116	0,0055	0,00326	0,00323	0,00331	0	3,46E-05	11
sum (n)	0,23879	0,20577	0,17148	0,15325	0,16937	0,17157	0,16933	0,14352	0,18928	0,17149	0,16948	0,16332	0,07217	0,03456	
sum (n *N)	1,22956	0,99949	0,75548	0,69243	0,75570	0,76059	0,75632	0,65270	1,12543	0,93629	0,91670	0,87134	0,27631	0,11366	
sum ($n*N^2$)	6,77222	5,20385	3,54814	3,37531	3,58381	3,58788	3,59462	3,29496	7,56539	5,87479	5,67198	5,36154	1,19878	0,45364	
SAP	5,15	4,86	4,41	4,52	4,46	4,43	4,47	4,55	5,95	5,46	5,41	5,34	3,83	3,29	
(w)SAP	5,51	5,21	4,70	4,87	4,74	4,72	4,75	5,05	6,72	6,27	6,19	6,15	4,34	3,99	
Đ	1,07	1,07	1,07	1,08	1,06	1,06	1,06	1,11	1,13	1,15	1,14	1,15	1,13	1,21	

3.3 Colorized graphs for the polysulfide distributions

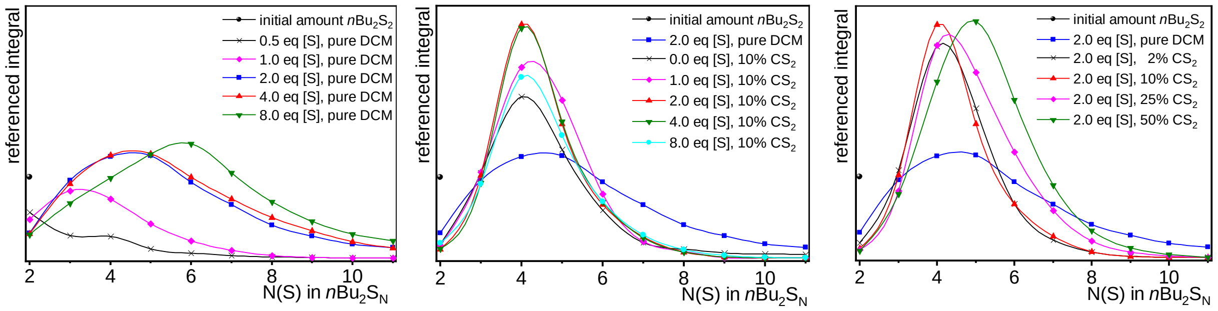

Figure S 76: Graphical presentation of the referenced HPLC-UV integrals in dependency of (left) sulfur amount added in DCM only, (centre) sulfur amount added in DCM with 10% (vol.) CS_2 and (right) varied amount of CS_2. Values taken from table S1.

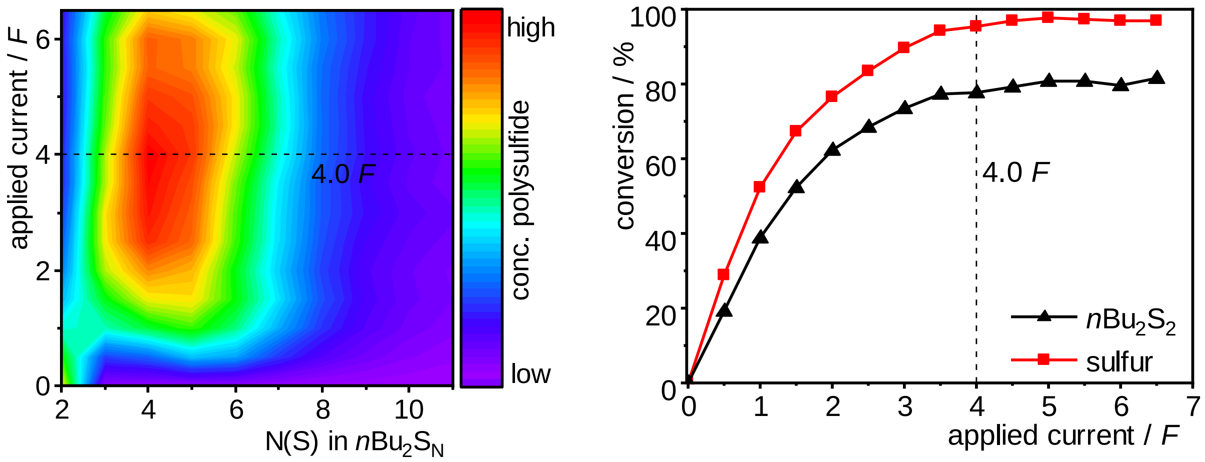

Figure S 77: Colorized presentation of (left) the relative amount of formed organic polysulfides determined by HPLC-UV and (right) conversion of the starting materials **1a** and elemental sulfur in dependence of the amplified current.
